# Cohort design and natural language processing to reduce bias in electronic health records research

**DOI:** 10.1038/s41746-022-00590-0

**Published:** 2022-04-08

**Authors:** Shaan Khurshid, Christopher Reeder, Lia X. Harrington, Pulkit Singh, Gopal Sarma, Samuel F. Friedman, Paolo Di Achille, Nathaniel Diamant, Jonathan W. Cunningham, Ashby C. Turner, Emily S. Lau, Julian S. Haimovich, Mostafa A. Al-Alusi, Xin Wang, Marcus D. R. Klarqvist, Jeffrey M. Ashburner, Christian Diedrich, Mercedeh Ghadessi, Johanna Mielke, Hanna M. Eilken, Alice McElhinney, Andrea Derix, Steven J. Atlas, Patrick T. Ellinor, Anthony A. Philippakis, Christopher D. Anderson, Jennifer E. Ho, Puneet Batra, Steven A. Lubitz

**Affiliations:** 1 Division of Cardiology, Massachusetts General Hospital, Boston, MA USA; 2 Cardiovascular Research Center, Massachusetts General Hospital, Boston, MA USA; 3 Cardiovascular Disease Initiative, Broad Institute of Harvard and the Massachusetts Institute of Technology, Cambridge, MA USA; 4 Data Sciences Platform, Broad Institute of Harvard and the Massachusetts Institute of Technology, Cambridge, MA USA; 5 Division of Cardiology, Brigham and Women’s Hospital, Boston, MA USA; 6 Department of Neurology, Massachusetts General Hospital, Boston, MA USA; 7 Henry and Allison McCance Center for Brain Health, Massachusetts General Hospital, Boston, MA USA; 8 Department of Medicine, Massachusetts General Hospital, Boston, MA USA; 9 Harvard Medical School, Boston, MA USA; 10 Division of General Internal Medicine, Massachusetts General Hospital, Boston, MA USA; 11 Bayer AG, Research and Development, Pharmaceuticals, Leverkusen, Germany; 12 Demoulas Center for Cardiac Arrhythmias, Massachusetts General Hospital, Boston, MA USA; 13 Eric and Wendy Schmidt Center, Broad Institute of Harvard and the Massachusetts Institute of Technology, Cambridge, MA USA; 14 Center for Genomic Medicine, Massachusetts General Hospital, Boston, MA USA; 15 Department of Neurology, Brigham and Women’s Hospital, Boston, MA USA

**Keywords:** Epidemiology, Outcomes research

## Abstract

Electronic health record (EHR) datasets are statistically powerful but are subject to ascertainment bias and missingness. Using the Mass General Brigham multi-institutional EHR, we approximated a community-based cohort by sampling patients receiving longitudinal primary care between 2001-2018 (Community Care Cohort Project [C3PO], *n* = 520,868). We utilized natural language processing (NLP) to recover vital signs from unstructured notes. We assessed the validity of C3PO by deploying established risk models for myocardial infarction/stroke and atrial fibrillation. We then compared C3PO to Convenience Samples including all individuals from the same EHR with complete data, but without a longitudinal primary care requirement. NLP reduced the missingness of vital signs by 31%. NLP-recovered vital signs were highly correlated with values derived from structured fields (Pearson *r* range 0.95–0.99). Atrial fibrillation and myocardial infarction/stroke incidence were lower and risk models were better calibrated in C3PO as opposed to the Convenience Samples (calibration error range for myocardial infarction/stroke: 0.012–0.030 in C3PO vs. 0.028–0.046 in Convenience Samples; calibration error for atrial fibrillation 0.028 in C3PO vs. 0.036 in Convenience Samples). Sampling patients receiving regular primary care and using NLP to recover missing data may reduce bias and maximize generalizability of EHR research.

## Introduction

Electronic health record (EHR) databases are increasingly recognized as powerful tools for biological discovery and clinical insight^1^. EHR databases provide favorable statistical power for large-scale association (e.g., epidemiological, genetic) analyses, rich and diverse feature sets including clinical risk factors, laboratory results, free-text notes, and raw imaging data^2–5^, and repeated measures to support modeling of disease progression and clinical trajectories^6^.

However, there is increasing recognition that EHR data may be subject to multiple biases related to patient selection, data acquisition, and misclassification or measurement error^7^. Two particularly important sources of bias include ascertainment bias resulting from the acquisition of data on the basis of clinical need^3,8,9^, as well as selection bias secondary to missingness^4,10,11^. Although pragmatic, the practice of sampling all individuals with relevant data for a particular modeling application may amplify ascertainment bias and missingness, leading to spurious associations and poor generalizability^2,7,9,11–13^. In contrast, intentional a priori sampling of individuals receiving regular primary care may reduce ascertainment bias by providing a mechanism for longitudinal data acquisition outside the context of illness (e.g., health maintenance visits). Furthermore, analysis of unstructured data, such as free text notes, may provide an opportunity to reduce bias related to missing data. Overall, both strategies attempt to reduce bias by constructing the EHR sample such that it more closely resembles a traditional research cohort, which may in turn increase the validity of applying established analysis methods typically utilized in the cohort study setting (e.g., survival analysis)^9^.

In the current study, we developed the Community Care Cohort Project (C3PO), a multi-institutional EHR-based cohort intended to empower discovery research in cardiovascular disease and designed to achieve two major goals: (1) to mitigate ascertainment bias, and (2) to minimize data missingness. We developed and implemented a deep natural language processing (NLP) model to recover four vital sign features using unstructured notes, and compared effective sample sizes before and after missing data recovery. We then deployed two established clinical risk scores, and compared model performance in C3PO to that observed in Convenience Samples constructed from the same parent EHR but including all individuals with sufficient data to calculate each score (i.e., with no requirement for regular in-network primary care). We hypothesized that such risk scores derived in prospective cohort settings would perform more favorably in C3PO, providing evidence of reduced bias.

## Results

### C3PO cohort

**Fig. 1:**
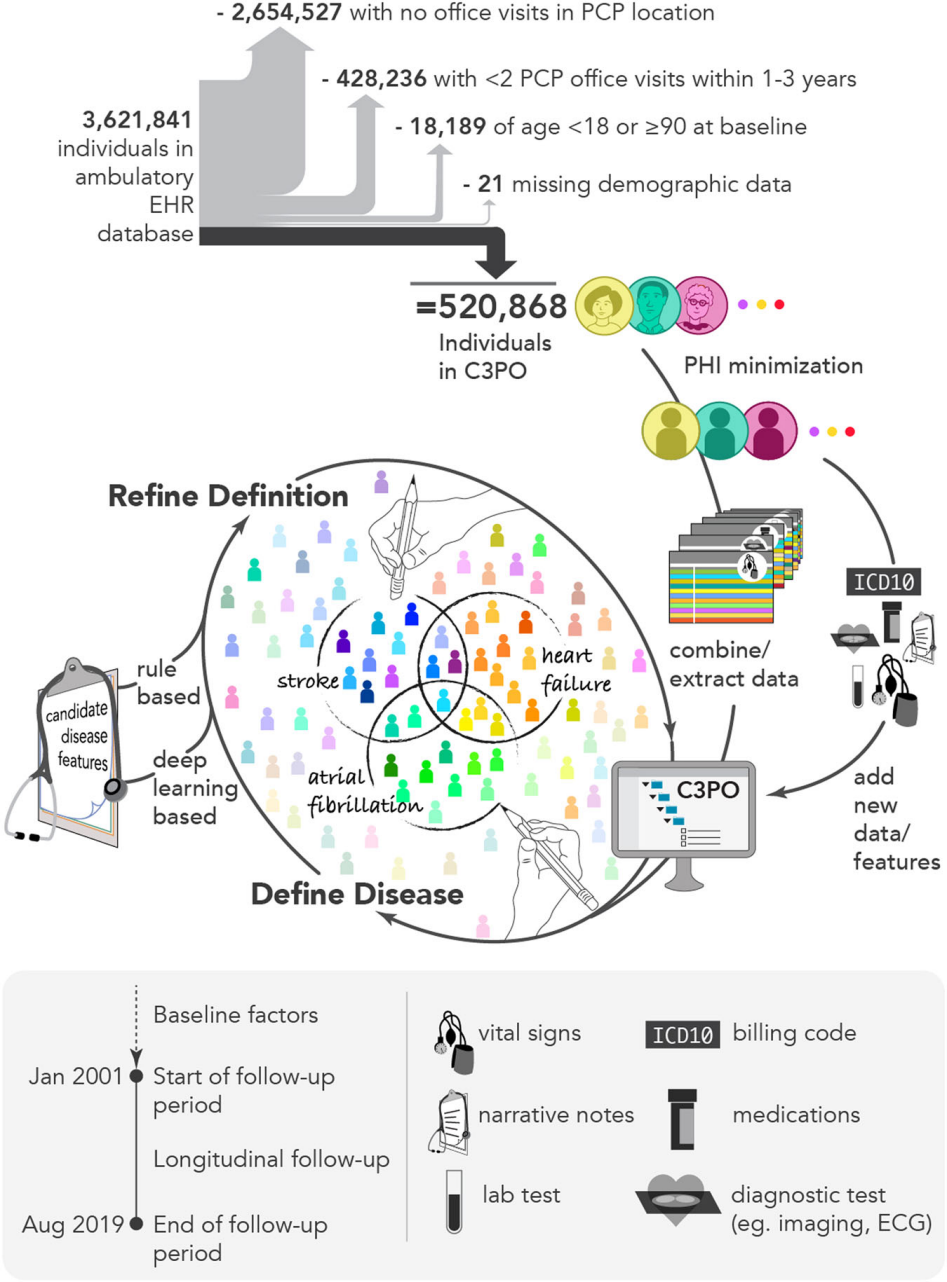
Overview of C3PO construction and data pipeline. Depicted is a graphical overview of the construction of the Community Care Cohort Project (C3PO). C3PO comprises the electronic health record (EHR) data of 520,868 individuals aged 18–90 at the start of sample follow-up, selected from an ambulatory EHR database on the basis of receiving periodic primary care (i.e., ≥2 visits within 1–3 consecutive years, see text). C3PO is structured as an indexed file system containing protected health information-minimized data of various types (bottom panel). The C3PO database can readily accommodate updating of existing data, integration of new data features, and construction of composite disease phenotypes based on multiple data features.

**Fig. 2:**
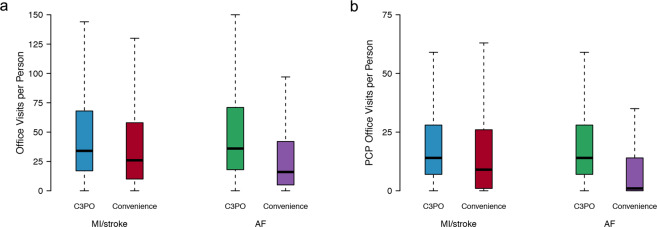
Distribution of office visits in C3PO versus Convenience Samples. Depicted are boxplots demonstrating the distribution of office visits (**a**) and primary care physician (PCP) office visits (**b**) in the C3PO analysis samples (AF [blue] and MI/stroke [green]) versus the respective Convenience Samples (AF [red] and MI/stroke [purple]). In each boxplot, the black bar denotes the median number of office visits per individual, the box represents the interquartile range, and the whiskers represent points beyond the interquartile range. Points greater than quartile 3 plus 1.5 times the interquartile range and points smaller than quartile 1 minus 1.5 times the interquartile range are not depicted.

In total, C3PO comprised 520,868 individuals (mean age 48 years, 61% women) with a median follow-up time of 7.2 years (quartile-1: 2.6, quartile-3: 12.9) (Fig. 1). Individuals in C3PO had a median of 30 office visits (14, 62), and 13 (6, 26) primary care office visits. By comparison, individuals in the Convenience Samples had shorter follow-ups and fewer office visits (Fig. 2 and Supplementary Fig 1). Characteristics of individuals in C3PO and each Convenience Sample are shown in Table 1. A summary of the diverse array of data types available for individuals in C3PO is shown in Supplementary Table 1.

**Table 1:** Baseline characteristics.

	C3PO^1^ (*N* = 520,868)	C3PO – MI/stroke (*N* = 198,184)^2^	MI/stroke Convenience Sample (*N* = 340,226)^2^	C3PO – AF (*N* = 174,644)^2^	AF Convenience Sample (*N* = 501,272)^2^
	Mean ± SD, Median (quartile 1, quartile 3), or *N* (%)				
Age (years)	48.4 ± 17.1	57.0 ± 10.3	56.2 ± 10.4	60.9 ± 10.0	61.4 ± 10.5
Women	315,577 (60.6%)	116,448 (58.8%)	195,039 (57.3%)	106,279 (60.9%)	288,334 (57.5%)
White	389,755 (74.8%)	154,712 (78.1%)	270,002 (79.4%)	140,746 (79.6%)	422,266 (84.2%)
Black	38,104 (7.3%)	13,805 (7.0%)	21,248 (6.2%)	11,103 (6.4%)	22,787 (4.5%)
Hispanic or Latino	33,762 (6.5%)	9401 (4.7%)	15,142 (4.5%)	6804 (3.9%)	14,115 (2.8%)
Asian or Pacific Islander	21,701 (4.2%)	7807 (3.9%)	13,219 (3.9%)	6003 (3.4%)	14,329 (2.9%)
Mixed	27 (0.05%)	11 (0.06%)	24 (0.07%)	7 (0.04%)	23 (0.04%)
Other	18,774 (3.6%)	5716 (2.9%)	8937 (2.6%)	4467 (2.6%)	9023 (1.8%)
Unknown	18,745 (3.6%)	6732 (3.4%)	11,654 (3.4%)	5514 (3.2%)	18,729 (3.7%)
Height (cm)	167.4 ± 10.4	–	–	166.6 ± 10.4	167.4 ± 10.3
Weight (kg)	78.3 ± 20.3	–	–	79.4 ± 19.5	79.8 ± 19.8
Systolic blood pressure (mmHg)	123 ± 17	126 ± 17	127 ± 18	128 ± 17	130 ± 19
Diastolic blood pressure (mmHg)	75 ± 10	–	–	76 ± 10	77 ± 11
Current smoker	27,202 (5.2%)	14,720 (7.4%)	12,652 (3.7%)	14,031 (8.0%)	22,020 (4.4%)
Anti-hypertensive use	147,898 (28.4%)	77,827 (39.3%)	119,954 (35.3%)	78,219 (44.8%)	173,235 (34.6%)
Diabetes	58,159 (11.2%)	29,307 (14.8%)	43,966 (12.9%)	27,953 (16.0%)	52,180 (10.4%)
Heart failure	12,555 (2.4%)	–	–	3334 (1.9%)	16,786 (3.3%)
Myocardial infarction	17,937 (3.4%)	–	–	6641 (3.8%)	18,260 (3.6%)
Total cholesterol (g/dL)	189 ± 39	195 ± 39	194 ± 40	–	–
HDL cholesterol (g/dL)	55 ± 18	57 ± 18	57 ± 18	–	–
Follow-up, years	7.2 (2.6, 12.9)	7.3 (2.8, 11.9)	7.4 (3.5, 11.8)	6.5 (2.5, 11.1)	5.4 (2.2, 9.8)

^1^Values shown exclude missing data.

^2^Only variables relevant for each risk score (CHARGE-AF for AF, PCE for MI/stroke) are depicted.

### NLP-based vital sign recovery

**Fig. 3:**
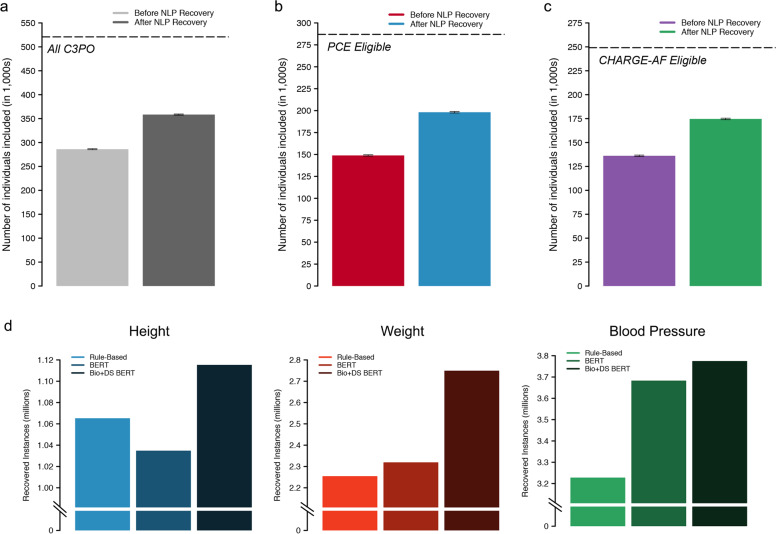
Yield of NLP-based missing data recovery. Depicted is a summary of the yield of our deep natural language processing (NLP) based model for missing data recovery in C3PO. **a**–**c** Compare effective sample sizes with versus without NLP recovery, where error bars depict 95% confidence intervals. **a** The *y*-axis depicts the total number of individuals with a baseline height, weight, and blood pressure, and the hashed line indicates the total sample size of C3PO. **b** The *y*-axis depicts the total number of individuals with a complete Pooled Cohort Equations (PCE) score at baseline and the hashed line indicates the total number of individuals eligible for PCE analysis (i.e., within age 40–79 years, with available follow-up data, and without prevalent MI/stroke). **c** The *y*-axis depicts the total number of individuals with a complete CHARGE-AF score at baseline and the hashed line indicates the total number of individuals eligible for CHARGE-AF analysis (i.e., within age 45–94 years, with available follow-up data, and without prevalent AF). **d** Depicts the total number of vital sign extractions obtained using the rule-based method (light shades), BERT (medium shades), and Bio + DischargeSummaryBERT (Bio + DS BERT, dark shades).

Using tabular data alone, 286,009 individuals (54.9%) had height, weight, systolic, and diastolic blood pressure available at baseline, which increased to 358,411 (68.8%) after deep learning-enabled NLP recovery (31% reduction in missingness, Fig. 3). NLP recovery rates stratified by vital signs are shown in Supplementary Table 2. An example clinical note with NLP-extracted vital sign values is shown in Supplementary Fig 2.

**Fig. 4:**
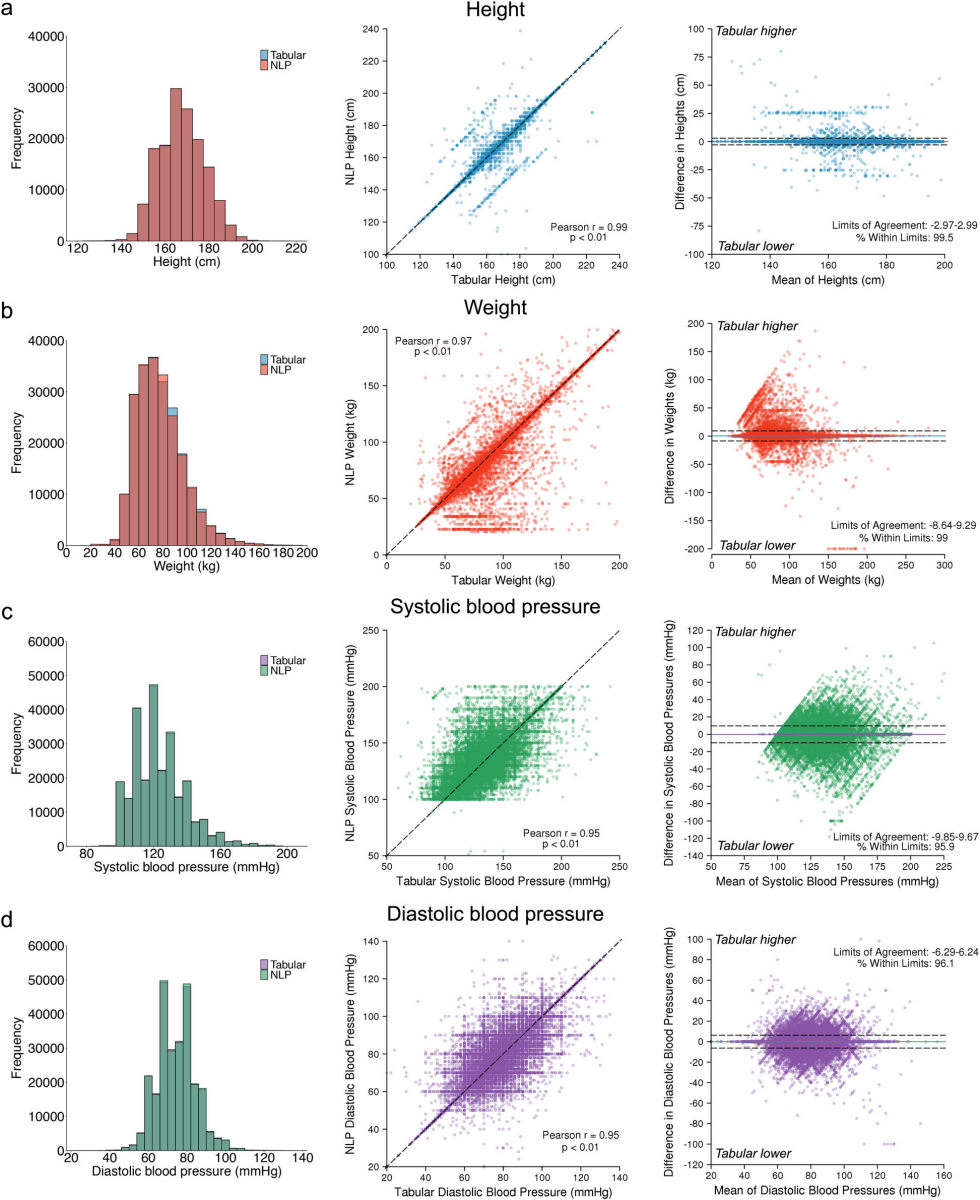
Agreement between tabular and natural language processing-extracted vital signs. Depicted is agreement between vital signs obtained from tabular data and those obtained from our NLP model among individuals with values obtained on the same day. **a** Depict height values, **b** depict weight values, **c** depict systolic blood pressures, and **d** depict diastolic blood pressures. For individuals with multiple eligible values, only the pair most closely preceding the start of follow-up was used. Left panels show the distribution of values obtained from tabular versus NLP sources. Middle panels show the correlation between tabular values (*x*-axis) and NLP values (*y*-axis). Right panels are Bland–Altman plots showing agreement between paired tabular and NLP values. The *x*-axis depicts the increasing mean of the paired values, and the *y*-axis depicts the difference between the paired values, where positive values denote tabular values greater than corresponding NLP values and negative values denote tabular values lower than corresponding NLP values. The colored horizontal lines depict the mean difference between sources, and the hashed horizontal lines depict 1.96 standard deviations above and below the mean. The values corresponding to the bounds and percentage of values contained within those bounds is printed on each plot.

When compared to a regular expression algorithm, the NLP approach resulted in a greater yield of each vital sign (Supplementary Table 3). Correlation between NLP-derived and tabular vital signs obtained on the same day was excellent (height *r* = 0.99, weight *r* = 0.97, systolic blood pressure *r* = 0.95, diastolic blood pressure *r* = 0.95, *p* < 0.01 for all, Fig. 4). Intra-individual agreement was good (95% limits of agreement for height: −2.97 cm–2.99 cm; weight: −8.64 kg–9.29 kg; systolic blood pressure: −9.85 mmHg–9.67 mmHg; diastolic blood pressure: −8.3 mmHg–8.2 mmHg). Bland–Altman plots did not suggest systematic bias (Fig. 4). High agreement was consistent by year of extraction (Supplementary Fig 3).

Where NLP-derived and tabular vital signs differed, specific failure modes included repeat measurements performed on the same day (e.g., repeat blood pressure measurement), transcription/typographical errors (e.g., 5'6'' entered as ‘56’ inches in the tabular data), and values referenced from prior encounters. Failure modes resulting in lack of vital sign detection by NLP included ambiguous labeling (e.g., “VS–142/92”) and atypical notation (e.g., “wgt 183”).

### Myocardial infarction (MI)/stroke analyses—Pooled Cohorts Equations (PCE)

A total of 198,184 individuals were included in incident MI/stroke analyses (Supplementary Fig 4). Of the 198,184 individuals, 49,289 (24.9%) would have been excluded in the absence of NLP-recovered data (Fig. 3). At 10 years, there were 10,201 MI/stroke events (cumulative risk 8.0%, 95% CI 7.8–8.1; incidence rate 8.4 per 1000 person-years, 95% CI 8.2–8.5). PCE model fit, discrimination, and calibration are summarized in Table 2. The sex- and race-specific PCE scores were each strongly associated with incident MI/stroke (hazard ratio [HR] per 1-standard deviation [SD] range 2.04–2.51 across the four scores), with moderate discrimination (c-index range 0.724–0.768) and some miscalibration (Greenwood-Nam-D’Agostino [GND] *χ*^2^ range 21–487; Integrated Calibration Index [ICI] range 0.012–0.030). Recalibration to the sample average MI/stroke risk did not improve calibration (GND χ^2^ range 18–1689; ICI range 0.010–0.034; calibration slope range 0.60–0.88). The model for White women had the highest discrimination (c-index 0.768, 95% CI 0.760–0.775) while the model for Black men was the best calibrated (GND *χ*^2^ 21, ICI 0.012, 95% CI 0–0.025, calibration slope 0.88, 95% CI 0.77–1.00). The distribution of predicted MI/stroke risk before and after recalibration is shown in Supplementary Fig 5. Cumulative risk of MI/stroke stratified by predicted risk using the original PCE models is shown in Supplementary Fig 6. Detailed assessments of PCE calibration before and after recalibration are shown in Supplementary Fig 7, 8. Results were similar in models deploying the White PCE algorithms only in White individuals (Supplementary Table 4). Model assessment excluding NLP-recovered values demonstrated similar performance metrics but with less precision (Supplementary Table 5).

**Table 2:** Risk score performance in C3PO versus Convenience Samples.

Model	Hazard ratio (per 1-SD increase)	C-index^3^ (95% CI)	GND $\chi^{2,4}$	Recalibrated GND $\chi^{2,4,5}$	ICI^6^ (95% CI)	Recalibrated ICI^5,6^ (95% CI)	Calibration slope^7^ (95% CI)
*C3PO*							
PCE (White women)^1^	2.51 (2.43–2.59)	0.768 (0.760–0.775)	487	1689	0.018 (0.017–0.020) *p* < 0.01	0.034 (0.031–0.037) *p* < 0.01	0.67 (0.65–0.70) *p* = 0.02
PCE (Black women)^1^	2.39 (2.17–2.64)	0.724 (0.702–0.746)	69	257	0.030 (0.023–0.036) *p* < 0.01	0.057 (0.050–0.064) *p* < 0.01	0.60 (0.53–0.67) *p* = 0.36
PCE (White men)^1^	2.17 (2.11–2.24)	0.738 (0.730–0.746)	361	618	0.024 (0.022–0.027) *p* < 0.01	0.032 (0.029–0.035) *p* < 0.01	0.70 (0.68–0.73) *p* = 0.59
PCE (Black men)^1^	2.04 (1.85–2.25)	0.725 (0.698–0.751)	21	183	0.012 (0–0.025) *p* = 0.06	0.010 (0–0.024) *p* = 0.83	0.88 (0.77–1.00) *p* = 0.84
CHARGE-AF^1^	2.56 (2.50–2.61)	0.782 (0.777–0.787)	1856	1367	0.028 (0.027–0.030) *p* < 0.01	0.019 (0.018–0.021) *p* < 0.01	0.77 (0.75–0.79) *p* < 0.01
*Convenience Samples*							
PCE (White women)^2^	2.44 (2.39–2.49)	0.770 (0.764–0.775)	1797	4923	0.032 (0.031–0.034)	0.047 (0.044–0.049)	0.64 (0.62–0.65)
PCE (Black women)^2^	2.29 (2.13–2.46)	0.732 (0.716–0.748)	213	562	0.046 (0.040–0.053)	0.074 (0.067–0.081)	0.56 (0.51–0.61)
PCE (White men)^2^	2.18 (2.13–2.22)	0.744 (0.739–0.749)	1291	1493	0.041 (0.038–0.043)	0.039 (0.037–0.042)	0.70 (0.68–0.71)
PCE (Black men)^2^	2.01 (1.88–2.15)	0.727 (0.705–0.749)	36	13	0.028 (0.018–0.037)	0.012 (0.0026–0.022)	0.87 (0.79–0.95)
CHARGE-AF^2^	2.40 (2.38–2.43)	0.781 (0.778–0.784)	7188	8322	0.036 (0.035–0.036)	0.028 (0.027–0.029)	0.69 (0.68–0.70)

^1^PCE (White women): 4231, 107,998, 7.1 (2.8, 10); PCE (Black women): 617, 8450, 7.7 (2.9, 10); PCE (White men): 4928, 76,304, 6.2 (2.3, 10); PCE (Black men): 425, 5432, 6.7 (2.5, 10); CHARGE-AF: *n* events = 7877, *N* total = 174,644, median follow-up, years (Q1,Q3): 5.0 (2.3,5.0).

^2^PCE (White women): 10,259, 182,349, 7.5 (3.6, 10); PCE (Black women): 1119, 12,690, 7.2 (3.2, 10); PCE (White men): 12,891, 136,629, 6.2 (2.6, 10); PCE (Black men): 843, 8558, 6.0 (2.6, 10); CHARGE-AF: *n* events = 26,907, *N* total = 501,272, median follow-up, years (Q1,Q3): 5.0 (2.0,5.0).

^3^C-index calculated using the inverse probability of censoring weighting method^28^.

^4^Greenwood-Nam-D’Agostino (GND) test, a test of calibration^30^. Lower chi-squared values suggest better calibration (across equally sized samples). Significant *p*-values indicate evidence of miscalibration. Corresponding *p*-values are all *p* < 0.01 except for C3PO PCE Black men (*p* = 0.02), C3PO PCE Black men recalibrated (*p* = 0.03), Convenience Sample PCE Black men recalibrated (*p* = 0.17).

^5^Values after recalibration to the baseline hazard of the sample (see text).

^6^Integrated calibration index, a quantitative measure of the average difference between predicted event risk and observed event incidence, weighted by the empirical distribution of event risk^29^. Smaller values indicate better calibration. *P*-values indicated pairwise comparison of ICI with the corresponding Convenience Sample.

^7^A measure of calibration applicable to models that are calibrated in the large^31,44^. A calibration slope equal to one is optimally calibrated. *P*-values indicated pairwise comparison of calibration slope with corresponding Convenience Sample.

*SD* standard deviation, *CI* confidence interval.

**Fig. 5:**
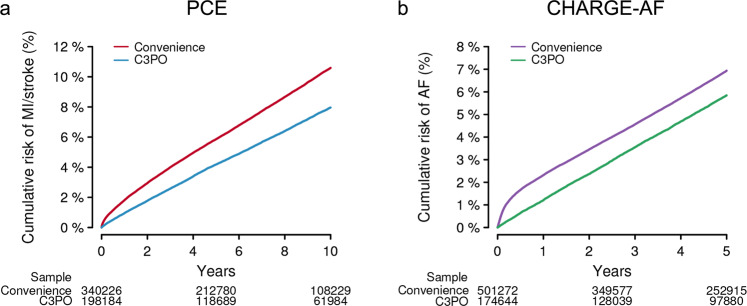
Cumulative event risk in C3PO versus Convenience Samples. Depicted is Kaplan–Meier cumulative risk of MI/stroke (**a**) and AF (**b**) observed in C3PO (blue [left] and green [right]) versus the Convenience Samples (red [left] and purple [right]). The number of individuals remaining at risk over time is labeled below each plot. Note an initial rapid inflection in MI/stroke and AF incidence observed in the Convenience Samples but not in C3PO.

**Fig. 6:**
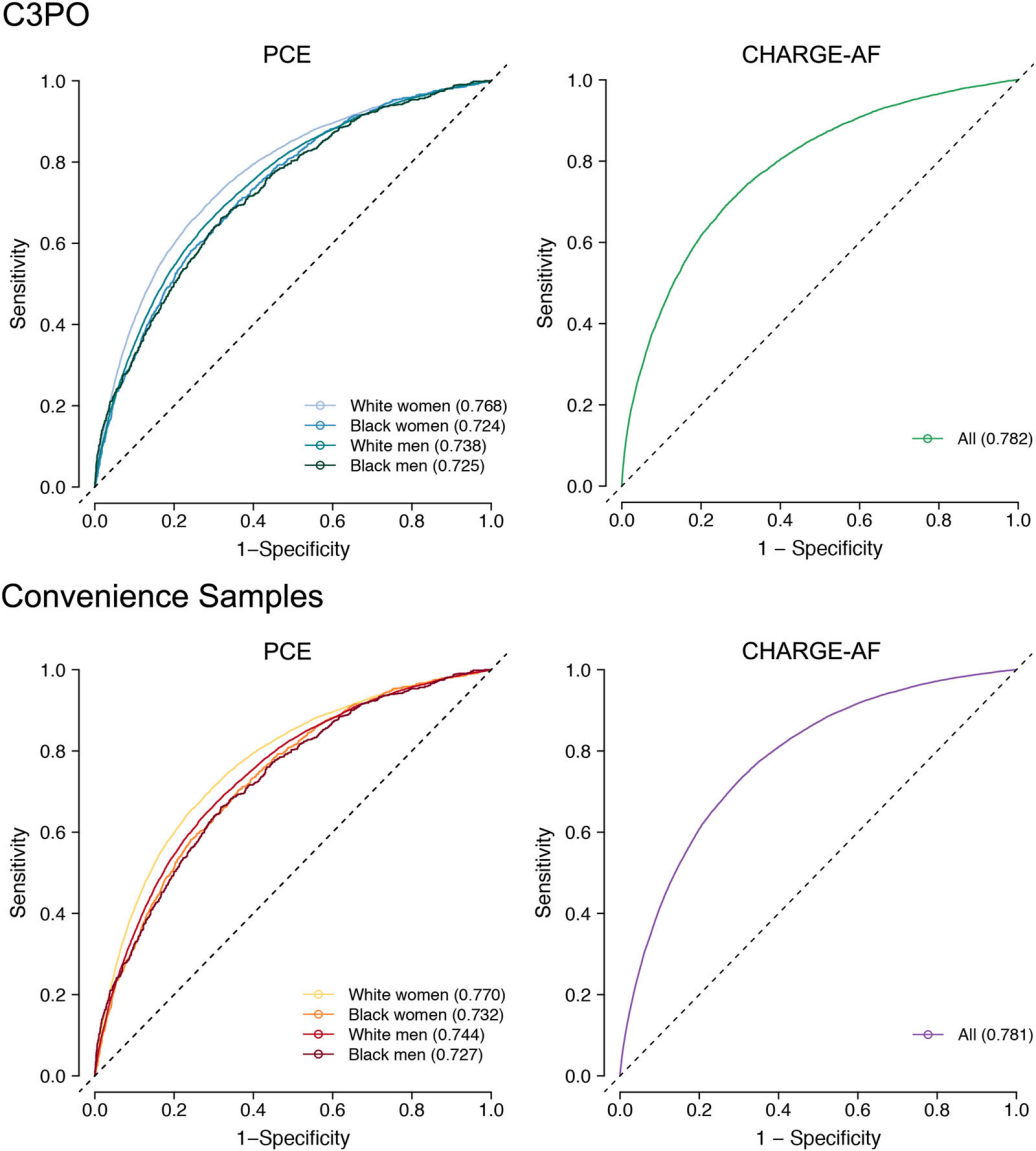
Model discrimination in C3PO and Convenience Samples. Depicted are time-dependent receiver operating characteristic curves for the Pooled Cohort Equations (PCE, left panels) and the CHARGE-AF score (right panels) in C3PO (top panels) versus the respective Convenience Samples (bottom panels). Each plot shows the discrimination performance of each risk score for its respective prediction target (i.e., 10-year MI/stroke for the PCE, 5-year incident AF for CHARGE-AF). Since the PCE score comprises four models stratified on the basis of sex and race, the curves for each score are represented separately (see legend). The c-index calculated using the inverse probability of censoring weighting method^28^ is depicted for each model.

**Fig. 7:**
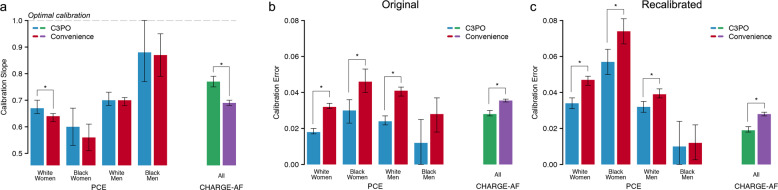
Model calibration in C3PO and Convenience Samples. Depicted is model calibration performance in C3PO versus the Convenience Samples. **a** Depicts the calibration slope for the PCE models (*x*-axis, left) and CHARGE-AF (*x*-axis, right) in C3PO (blue, green) versus the Convenience Samples (red, purple). The *y*-axis depicts the calibration slope, a measure of the relationship between predicted event risk and observed event incidence, where a slope of one indicates an optimal relationship (horizontal hashed line), with corresponding 95% confidence intervals. **b**, **c** Compare calibration error in C3PO versus the Convenience Samples. Calibration error is depicted on the *y*-axis using the Integrated Calibration Index (ICI, see text), where lower values indicate better absolute agreement between predicted risk and observed event incidence. **b** Depicts ICI values using the original models, while **c** depicts ICI values after recalibration to the baseline hazard of each sample. In all plots, statistically significant differences between values in C3PO versus the Convenience Sample (*p* < 0.05) are depicted with an asterisk.

We performed an analogous assessment of the PCE models within the MI/stroke Convenience Sample, which comprised 340,226 individuals. Compared to C3PO, the MI/stroke Convenience Sample had lower rates of cardiovascular comorbidity (Table 1). However, the observed 10-year MI/stroke risk was higher (cumulative risk 10.6%, 95% CI 10.5–10.7; incidence rate 11.7 per 1000 person-years, 95% CI 11.5–11.8). Cumulative risk curves demonstrated an abrupt rise in incident MI/stroke diagnoses shortly after the start of follow-up, which was not observed in C3PO (Fig. 5). Discrimination of MI/stroke risk was similar to that observed in C3PO (c-index range 0.727–0.770, Fig. 6). Calibration was worse than C3PO for all four models, although the difference was not statistically significant for Black men (GND *χ*^2^ range 36–1,797; ICI range 0.028–0.046; calibration slope range 0.56–0.87, Fig. 7 and Supplementary Figs 7, 8). Recalibration to the baseline hazard of the Convenience Sample did not correct miscalibration (GND *χ*^2^ range 13–4,923; ICI range 0.012–0.047, Fig. 7 and Supplementary Figs 7, 8).

### AF analyses—CHARGE-AF

A total of 174,644 individuals were included in incident AF analyses (Supplementary Fig 4). Of the 174,644 individuals, 38,528 (22.1%) would have been excluded in the absence of NLP-recovered data (Fig. 3). At 5 years, there were 7,877 AF events (cumulative risk 5.8%, 95% CI 5.7–6.0; incidence rate 12.1 per 1000 person-years, 95% CI 11.8–12.3). Details of CHARGE-AF model fit, discrimination, and calibration are shown in Table 2. The CHARGE-AF score was strongly associated with incident AF (HR per 1-SD 2.56, 95% CI 2.50–2.61), with moderate discrimination (c-index 0.782, 95% 0.777–0.787), although CHARGE-AF substantially underestimated AF risk (GND *χ*^2^ 1,856, ICI 0.028, 95% CI 0.027–0.030). After recalibration to the baseline AF hazard in C3PO, calibration was improved (GND *χ*^2^ 1,367; ICI 0.019, 95% CI 0.018–0.021; calibration slope 0.77, 95% CI 0.75–0.79). The distribution of predicted AF risk before and after recalibration is shown in Supplementary Fig 5. The cumulative risk of AF stratified by predicted AF risk is shown in Supplementary Fig 6. Detailed assessments of CHARGE-AF calibration before and after recalibration are shown in Supplementary Figs 7, 8. Model assessment excluding NLP-recovered values demonstrated similar performance metrics but with less precision (Supplementary Table 5).

We performed an analogous assessment of CHARGE-AF within the AF Convenience Sample, which comprised 501,272 individuals. Similar to observations with MI/stroke, individuals in the AF Convenience Sample had lower rates of cardiovascular comorbidity (Table 1), yet higher 5-year AF risk (cumulative risk 6.9%, 95% CI 6.9–7.0; AF incidence rate 15.1 per 1000 person-years, 95% CI 14.9–15.3). Cumulative risk curves again demonstrated an abrupt rise in incident AF diagnoses shortly after the start of follow-up, which was not observed in C3PO (Fig. 5). Discrimination of AF risk using CHARGE-AF was similar to that observed in C3PO (c-index 0.781, 95% CI 0.778–0.784, Fig. 6), but calibration was significantly worse (GND *χ*^2^ 7188; ICI 0.036, 95% CI 0.035–0.036; calibration slope 0.69, 95% CI 0.68–0.70, *p* < 0.01 for comparisons of ICI and calibration slope to C3PO, Fig. 7). Calibration remained less favorable in the Convenience Sample after recalibration to the baseline hazard (GND *χ*^2^ 8322; ICI 0.028, 95% CI 0.027–0.029; Fig. 7 and Supplementary Figs 7, 8).

## Discussion

In the present study, we demonstrate that intentional sampling of individuals from a large multi-institutional EHR on the basis of longitudinal primary care encounters, and recovery of missingness using deep learning, enable EHR-based prediction with validity exceeding a conventional EHR sampling approach^10,14,15^. C3PO comprises over a half-million individuals receiving longitudinal care over a decade of follow-up and, owing to the fact that it more closely mirrors the design of epidemiologic cohort studies, is likely to facilitate more generalizable insights^7,9^. When compared to Convenience Samples derived from the same parent EHR with no requirement for longitudinal primary care, C3PO appeared less biased and offered greater data density. Leveraging neural network-based NLP models using unstructured notes, we achieved a 31% reduction in missingness of baseline vital signs.

**Fig. 8:**
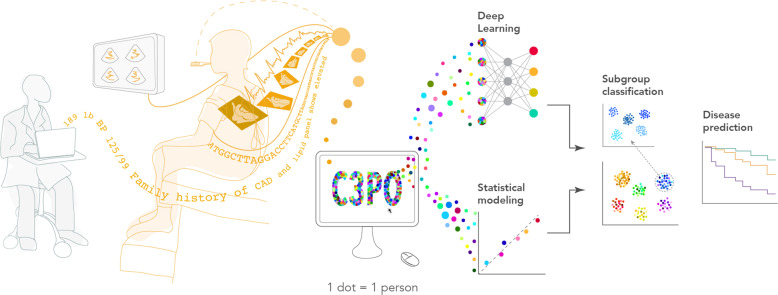
Conceptual overview of C3PO analysis methods. Depicted is a graphical overview of the potential analyses enabled by the Community Care Cohort Project (C3PO). By integrating diverse data types (e.g., diagnoses, imaging, vital signs, diagnostic test data, genetics), C3PO may enable methods such as traditional statistical modeling and deep learning to facilitate more accurate disease risk prediction models and enable deep phenotyping including disease subgroup identification.

The JEDI Extractive Data Infrastructure (JEDI) pipeline underlying C3PO, which we have made publicly available, provides a modular framework for processing and updating diverse EHR data in a manner conducive to multiple modeling approaches. We submit that JEDI, along with the principles underlying the development of C3PO, may enable future discovery by facilitating novel statistical and machine learning-based prediction and classification models utilizing diverse EHR data types available at scale and in a manner that reduces bias (Fig. 8). The principles guiding the development of C3PO and the coding infrastructure for our analyses are widely extendable to external EHR datasets.

There is increasing recognition that EHR datasets represent a potentially powerful resource for the development of traditional^10^ and machine learning-based^14,16^ prognostic models, yet at the same time may be particularly susceptible to biases which may lead to systematic error in effect estimates or poor generalizability^7,9,17^. To that end, our observations provide important evidence that EHR samples enriched for individuals receiving longitudinal primary care may offer a particularly efficient method for developing novel disease-related models in a manner that reduces bias. In the current study, we performed incident disease modeling using C3PO versus Convenience Samples including all individuals with complete data but with no requirement for longitudinal primary care. Despite paradoxically lower rates of documented cardiovascular comorbidity, MI/stroke and AF incidence rates were higher in the Convenience Samples as opposed to C3PO. We suspect that this asymmetry in disease incidence may be related to ascertainment bias such that individuals in the Convenience Samples are more likely to have complete data acquired because of higher disease risk. We acknowledge that alternative methods for assessing and mitigating bias exist, such as identification of specific missingness mechanisms and application of inverse probability weighting^9^. Future work is warranted to assess how such methods compare or add to bias mitigation strategies taken at the level of sample construction. Nevertheless, we submit that selecting for individuals receiving longitudinal primary care appears to reduce ascertainment bias by providing a mechanism for data acquisition outside the context of a specific illness (e.g., health maintenance visits)^9^.

Similarly, we observed abrupt increases in incident diagnoses shortly after the start of follow-up in the Convenience Samples. It is possible that such a pattern may represent misclassification of prevalent disease as an incident. By defining the start of follow-up as the second of the two qualifying PCP visits required for inclusion in C3PO, we submit there is a greater likelihood for prevalent conditions to be appropriately recorded within the EHR prior to the onset of time-to-event analyses, reducing misclassification of prevalent disease. Taken together, the performance of established risk models, each derived in traditional prospective cohorts, was more consistent with expectations when deployed within C3PO as opposed to the Convenience Samples. Specifically, the discrimination performance of the PCE and CHARGE-AF scores in C3PO was comparable to metrics reported in each score’s original validation study^18,19^. Furthermore, when compared to the Convenience Samples, model calibration was favorable in C3PO, demonstrating a relationship between known risk factors and outcomes more consistent with prior evidence^18,19^. Nevertheless, we note model calibration in C3PO was still not optimal. Future work is needed to better understand whether differences in performance may be related to residual bias versus differences in baseline comorbidity profiles^20^, and whether more advanced recalibration or reweighting techniques utilizing EHR data may provide an opportunity to optimize the performance of traditional risk models^21^.

We acknowledge that selecting a primary care population may introduce alternative biases (e.g., more likely to have insurance), which requires further study. Of note, EHR sample construction predicated on the needs of a specific analysis may also produce datasets that are less adaptable to other analytic frameworks^22–24^. In contrast, the C3PO sampling design is readily amenable to an array of epidemiologic analyses (e.g., cross-sectional, retrospective cohort, case-control).

Our findings also imply that deep learning models applied to unstructured data have the potential to substantially reduce missingness, another potential source of bias in EHR-based analyses^7^. We leveraged neural network-based NLP methods to accurately extract vital signs for an additional 80,000 individuals using unstructured text, reducing missingness by roughly one-third. Use of NLP resulted in substantially more vital sign extractions at high accuracy when compared to regular expressions alone, with consistent performance over time. Importantly, vital signs obtained from tabular and NLP sources were consistently very highly correlated, with good agreement. We anticipate that analogous NLP models may be able to extract additional clinical parameters, such as laboratory values, which continue to exhibit substantial missingness in C3PO. Importantly, such future models may require the ability to harmonize values across a wider range of potential units of measure and varying assays. We note that although risk model performance metrics in C3PO did not change substantively with NLP recovery, metric estimates had less uncertainty, suggesting that the primary effect of NLP recovery in our sample was an improvement in statistical power. The ability to provide more precise risk estimates (i.e., less uncertainty) may be clinically important. Given that missingness mechanisms in EHR samples are frequently complex and non-random^9^, we submit that recovery of actual data where possible is preferable to other methods of accounting for missingness, many of which rely on missingness at random. Nevertheless, future work is needed to better understand how NLP-based recovery compares to substitution methods such as multiple imputation^25^.

We submit that large and comprehensive EHR samples like C3PO have the potential to facilitate broad-ranging discovery leveraging diverse data types, provided that sufficient infrastructure exists to efficiently process, store, and analyze data within a unified framework. To that end, we have developed the JEDI pipeline, which automates the processing and unification of diverse EHR data types within a harmonized, indexed file system amenable to a variety of statistical and machine learning-based approaches. Specifically, C3PO includes over 2.95 million ECGs, 450,000 echocardiograms, and millions of free-text notes. Through linkage to the MGB Biobank biorepository, we anticipate that biological samples will be available within over 40,000 individuals. Facilitated by the JEDI pipeline, we expect that future models built within C3PO leveraging some or all of these data types will result in more accurate and generalizable disease prediction and classification models. Importantly, although the EHR data comprising C3PO is not sharable owing to concerns about data identifiability, the principles governing C3PO are widely applicable to EHR datasets and the JEDI pipeline is publicly available to catalyze future research efforts related to the development of clinical models using rich and diverse EHR data.

Our study should be interpreted in the context of design. First, despite our intent to reduce bias by selecting individuals receiving regular in-network primary care, residual indication bias is inevitable using EHR data. Nevertheless, by applying clinical risk scores and assessing disease incidence rates, we demonstrate that the approach taken to developing C3PO appears to reduce bias. We acknowledge that alternative methods for quantifying bias (e.g., assessment of phenome-wide associations, genetic association testing) exist, but opted to focus on clinical risk scores given their clinical utility. Second, although we successfully employed NLP to reduce missingness rates for vital signs by roughly one-third, missingness of other features (e.g., cholesterol) remains considerable. We anticipate that similar NLP approaches will have utility in reducing missing data further, although we acknowledge that certain features (e.g., imaging characteristics, laboratory values) may be more challenging to extract. Third, the performance of our NLP model in other datasets remains unknown, although we anticipate that our overall approach of utilizing pre-trained language models with fine-tuning in the same or similar samples as those in which implementation is intended is likely to result in good performance across datasets. Fourth, although we utilized previously validated algorithms to define the presence of disease, some degree of misclassification of exposures and outcomes remains likely. Fifth, we identified individuals for inclusion in C3PO using EHR-based codes to identify office visits and a manually curated list of in-network primary care practice locations. Although two forms of validation support the accuracy of our selection methods, we acknowledge that the process is imperfect and would not easily extend to other EHRs. Sixth, most individuals included in C3PO are White, and therefore generalizability to populations with varying racial composition may be limited. However, we note that the absolute number of individuals of color within C3PO compares favorably to several other cohorts and EHR-based studies^26–28^. Seventh, current results are observational and should not be used to infer causality.

In conclusion, we have developed C3PO, an EHR-based resource comprising over a half-million individuals within a large networked healthcare system. By sampling the full range of EHR data for individuals receiving regular primary care and providing a mechanism for the use of NLP to recover data from unstructured notes, EHR samples such as C3PO offer the potential to substantially reduce biases related to patient selection and missing data. By providing a broad array of data types, longitudinal measurements, and a flexible data structure conducive to multiple modeling frameworks, we anticipate that C3PO—and similarly constructed EHR datasets—will facilitate impactful discovery research.

## Methods

### Cohort construction

Mass General Brigham (MGB) is a multi-institutional healthcare network with a linked EHR spanning seven tertiary care and community hospitals with associated outpatient practices in the New England region of the United States. Participants were identified using an MGB-based data mart containing tabular EHR data for >3.6 million individuals with ≥1 ambulatory visit between 2000–2018. Given our intent to identify individuals receiving primary care within MGB, we developed, validated, and applied rule-based heuristics to identify primary care office visits using Current Procedural Terminology (CPT) codes (Supplementary Table 6) and a manually curated list of 431 primary care clinic locations. To select individuals receiving longitudinal primary care within MGB, we restricted the cohort to individuals with at least one pair of primary care visits occurring between 1–3 years apart. To facilitate ascertainment of baseline clinical factors, we defined the start of follow-up for each individual as the second primary care visit of that individual’s earliest qualifying pair (Supplementary Fig 9)^4^. Study protocols complied with the tenets of the Declaration of Helsinki and were approved by the MGB Institutional Review Board.

### Cohort validation

We validated the construction of C3PO using two methods. First, we assessed overlap between individuals selected for C3PO and an existing sample from a curated Massachusetts General Hospital (MGH) primary care practice registry, to which we applied analogous selection methods. Specifically, we analyzed individuals who were represented in the MGH registry in ≥2 consecutive years between 2005-2017.

Of 280,815 individuals in the MGH registry meeting the specified temporal selection criteria, the substantial majority (*n* = 206,868; 73.7%) were represented in the candidate C3PO cohort (Supplementary Fig 10). The remaining discrepancy was attributed to differences in the application of temporal selection criteria (1–3 year windows based on exact dates in C3PO, versus only calendar year data available in MGH registry), as well as the exclusion of individuals aged <18 years at the start of follow-up in C3PO. Without the application of temporal or age selection criteria, 277,780 out of 297,718 (93.3%) of the MGH registry was represented in C3PO (Supplementary Fig 10).

Second, we performed manual validation of the EHR for C3PO candidates. Two clinical adjudicators blinded to C3PO selection algorithm status performed a manual chart review of 200 randomly selected algorithm-positive individuals (“algorithm-positive”) and 200 randomly selected individuals with ≥1 office visit but none in a primary care location (“algorithm-negative”). A total of 60 algorithm-positive and 60 algorithm-negative records were overlapping between the adjudicated sets in order to assess inter-rater reliability. In certain locations within MGB, the clinically accessible EHR lagged behind the availability of data sources from the data mart. As a result, the ability to adjudicate the presence of primary care office visits in the earliest years of the C3PO cohort was limited. Frequently, there was indirect evidence of longitudinal primary care (e.g., notes making explicit mention of previous visits not available in the clinical EHR). As a result, we specified a priori two levels of adjudication for algorithm-positive individuals. In Tier 1, algorithm-positive individuals would be adjudicated as positive only if there was a narrative note confirming a primary care office visit (within ±7 days) on each of the two visit dates of interest. In Tier 2, algorithm-positive individuals would be adjudicated as positive if there was at least one pair of narrative notes confirming two primary care office visits 1–3 years apart (i.e., the inclusion criteria for C3PO) at any time in the individual’s EHR history. In all cases, algorithm-negative individuals were adjudicated as correct if there was not a primary care office visit on both the index date (within ±7 days) and within 1–3 years following the index date (i.e., the inclusion criteria for C3PO). The results of the adjudication process are summarized in Supplementary Table 7. Inter-rater agreement was excellent (kappa range 0.78–1). Both case and non-case algorithms met pre-specified criteria for sufficient algorithm accuracy (PPV ≥ 85%) to proceed with C3PO construction.

### Data ingestion pipeline

After identifying a candidate set of 523,445 individuals in C3PO, we obtained a comprehensive range of EHR data including demographics, anthropometrics and vital signs, narrative notes, laboratory results, medication lists, and radiology/cardiology diagnostic test reports using the Research Patient Data Registry (Boston, Massachusetts), a data repository containing the complete EHR data of all individuals receiving care within MGB^29^. We then developed a standardized data ingestion pipeline (the JEDI Extractive Data Infrastructure [JEDI]), which integrates a series of distinct files containing an array of different EHR data types into a unified, indexed file system (Hierarchical Data Format [HDF] 5^30^). To facilitate interactive data exploration and epidemiologic modeling, we also developed egress pipelines capable of producing customized long-format files (i.e., each row is a distinct observation within the EHR) and wide-format files (i.e., each row is a unique individual and columns represent data summarized from multiple observations).

The JEDI pipeline is implemented in Python with minimal dependencies allowing it to be run on most common platforms. Since MGB source data is restricted, all data processing for this study utilized JEDI run on a secure MGB Linux cluster. A one-time ingestion process to convert plain text data to HDF5 (mean size 34 MB) takes about 1 min per individual’s full EHR record. Once in this format, long-file and wide-file processing scales with the number and complexity of the features under consideration. For example, to extract a broad range of features relevant for cardiovascular disease, we are able to produce ~5 long-format files (mean size 260 KB) per minute and 300 wide-format file rows per minute (500 MB for full file). To maximize runtime efficiency, JEDI is designed to schedule an arbitrary number of jobs through the IBM (Armonk, NY) Spectrum LSF workload management platform.

For C3PO, we removed individuals aged <18 or ≥90 years at the start of follow-up, as well as an additional 21 individuals with missing demographic data, resulting in 520,868 individuals in the final cohort (Fig. 1).

### Implementation of clinical scores to assess bias

We then sought to assess the validity of C3PO by implementing two well-validated cardiovascular risk prediction models, each derived in traditional community-based cohorts: (1) the Pooled Cohort Equations (PCE)^17^, and (2) the Cohorts for Aging and Genomic Epidemiology Atrial Fibrillation (CHARGE-AF) score^19^. Since both scores have exhibited relatively consistent discrimination and calibration (i.e., an agreement between predicted risk and observed disease incidence) in multiple community cohorts^18,19,31,32^, we considered systematic error in score performance (e.g., miscalibration, or disagreement between predicted risk and observed disease incidence) as probable evidence of bias present within the underlying sample^9^.

Relevant exposures were derived from the EHR. Demographics including age, sex, and race were extracted from dedicated demographic fields. Height, weight, blood pressure, and smoking status were derived from tabular EHR data extracted from clinical encounters, where the value most closely preceding the start of follow-up (within 3 years) was used, with the exception of height for which any value in the EHR was accepted. The PCE score includes a term for systolic blood pressure, and the CHARGE-AF score includes terms for height, weight, systolic blood pressure, and diastolic blood pressure (full components of each score shown in Supplementary Tables 8, 9). Prevalent diseases were defined using previously published groupings of International Classification of Diseases, 9th and 10th revision (ICD-9 and 10) diagnosis codes and CPT codes^4,33^. All exposure definitions are shown in Supplementary Table 10.

Primary outcomes included the prediction targets for each risk score (i.e., MI/stroke for PCE and AF for CHARGE-AF). AF was defined using a previously validated EHR-based AF classification scheme (PPV 92%)^34^. MI and stroke were defined using the presence of ≥2 ICD-9 or ICD-10 codes using previously validated code sets (PPV ≥ 85%)^33^.

### Construction of MI/stroke and AF Convenience Samples

We then assessed whether C3PO may exhibit less bias as compared to an alternative EHR sampling approach. Specifically, we compared the performance of the PCE and CHARGE-AF scores in C3PO to that observed within samples derived from the same parent EHR but constructed solely on the basis of available score components (“PCE Convenience Sample” and “CHARGE-AF Convenience Sample”). We utilized convenience sampling as a comparator because convenience sampling may maximize statistical power^17^, and has been utilized in several recent EHR-based studies^2,9–11^.

From the source mart (*N* = 3.6 million, Fig. 1), all individuals with available data for each component of the PCE (MI/stroke Convenience Sample) and each component of the CHARGE-AF score (AF Convenience Sample) were identified, with no requirement for primary care office visits. To maximize the available sample size, we defined the start of follow-up for each individual as the earliest time at which all score components were available for that individual. We then excluded individuals who did not have all score components available within 3 years prior to each individuals’ start follow-up date. We also excluded individuals with no follow-up data of any kind, as well as those having the relevant outcome at the start of follow-up. Disease-related exposure and outcome ascertainment was performed on the basis of at least one ICD, CPT, and/or EHR-specific diagnosis code present in the EHR mart corresponding to the relevant disease. In each Convenience Sample, the start of follow-up began at the earliest time all necessary data became available, and the end of follow-up was defined as the last encounter of any type in the EHR. Individuals with zero follow-up time were not included in incident analyses. Flow diagrams summarizing the construction of the Convenience Samples are shown in Supplementary Fig 11.

### Natural language processing to reduce vital sign missingness

NLP methods have recently shown promising results for extracting information from unstructured text-based data^35^. Advanced neural network models such as Bidirectional Encoder Representations from Transformers (BERT)^36^ have immense expressive power as their representations are derived from training on large corpora of text. These models can also be further pre-trained on domain-specific languages, such as biomedical and clinical text. This has been shown to improve performance on a number of clinical NLP tasks when compared to general language embeddings, including tasks such as named entity recognition and diagnostic inference^37,38^. This bio-clinical-specific pre-training allows such models to be fine-tuned using relatively small amounts of “weakly” labeled data generated by a rule-based approach and perform exceptionally well on downstream tasks such as feature extraction from free-text notes. In the current study, we utilized Bio + Discharge Summary BERT, a deep contextual word embedding model that has been pre-trained consecutively on a large corpus of general English text (e.g., Wikipedia), biomedical text (PubMed abstracts and PubMed Central full-text articles)^38^, and physician-written Discharge Summaries (from the MIMIC-III v1.4 database)^37,39^.

Given high missingness rates for baseline vital signs (>40%), we employed Bio + Discharge Summary BERT to recover height, weight, and systolic and diastolic blood pressures from unstructured notes. To label potential vital signs, we created a regular expression rule-based approach to automatically label the position of vital sign values in several different types of clinical notes. Table 3 demonstrates the context words, unit tokens, and text patterns we considered for each feature, in addition to an example of labeled text in each case. To build the dataset we selected 900 individuals and labeled all 34,310 eligible notes in the 3 years prior to the start of follow-up. The distribution of note types in the training set was as follows: inpatient or outpatient history and physical (*n* = 32,186, 93.8%), discharge summary (*n* = 1,316, 3.8%), cardiology note (*n* = 796, 2.3%), and endoscopy report (*n* = 12, 0.03%). This resulted in a total of 116,644 instances of labeled vitals—37,679 instances of blood pressure, 58,910 instances of weight, and 20,055 instances of height. We also created two additional independent sets for evaluation and testing respectively. We sampled 2038 notes from 50 individuals to use as the evaluation set, which contained 7230 vital identifications—2167 instances of blood pressure, 3979 instances of weight, and 1084 instances of height. Similarly, we labeled 1852 notes from another 50 individuals to produce an independent test set, which comprised 6178 vital identifications—2025 instances of blood pressure, 3032 instances of weight, and 1121 instances of height. The training set consisted of 80-word spans surrounding these labeled tokens in order for the model to learn the context in which vital signs tend to appear. We also included some spans of the same length with no labeled values, so that the model could learn the overall structure of the notes.

**Table 3:** Regular expression rule-based approach for vital sign labeling.

Vital Sign	Context Words	Units Considered	Text Patterns	Labeled Example In format: word {LABEL}
Height	“height”, “height:”, “ht”, “ht:”	‘inches’, ‘in’, ‘feet’, ‘ft’, ‘m’, ‘meters’, ‘cm’, ‘centimeters’, ''' (for feet) '''' (for inches)	[number]	Ht: 63.5 {HEIGHT}
			[number] [unit]	Patient height is 63.5 {HEIGHT} inches {HEIGHT_UNIT}
			[number] [unit] [number] [unit]	Height: 5 {HEIGHT} feet {HEIGHT_UNIT} 11 {HEIGHT} inches {HEIGHT_UNIT}
Weight	“weight”, “weight:”, “wt”, “wt:”	‘pounds’, ‘lbs’, ‘lb’, ‘ounces’, ‘oz’, ‘kilograms’, ‘kg’, ‘grams’, ‘g’	[number]	Wt: 180 {WEIGHT}
			[number] [unit]	Current weight is 65.9 {WEIGHT} kg {WEIGHT_UNIT}
			[number] [unit] [number] [unit]	Patient’s weight is 170 {WEIGHT} lbs {WEIGHT_UNIT} 9 {WEIGHT} oz {WEIGHT_UNIT}
Blood Pressure	“pressure”, “bp”, “bp:”	–	[number]/[number]	Blood pressure is 128/70 {BP}

Bio+Discharge Summary BERT was fine-tuned for 5 epochs with the task of labeling the values identified by the rule-based approach. Categorical cross-entropy was used as the training metric. We did not employ any additional regularization methods, and early stopping was not used. Training and evaluation curves for model fine-tuning are illustrated in Supplementary Fig 12. Given some suggestions of potential overfitting with 5 epochs of training, we assessed a version of Bio + Discharge Summary BERT trained for 2 epochs, which had a similar post-processed performance as the original model but had substantially fewer successful blood pressure extractions (Supplementary Table 11). We also compared our model utilizing Bio + Discharge Summary BERT to an analogous model trained using the original BERT^36^, which demonstrated similar high accuracy (96% for weight, 100% for height, and 100% for blood pressure), but lower yield for each vital sign (Supplementary Table 3).

To estimate the amount of training data that would be needed to recreate our approach, we also created four additional training sets that corresponded to subsets of our current training data. We held all other parameters constant, and fine-tuned four additional Bio+Discharge Summary BERT models for 5 epochs each. Supplementary Fig 13 illustrates the final training and evaluation loss for models trained on different sizes of training data. We see that both the training and evaluation loss continue to decrease as training set size increases and that a larger number of labeled vital signs boosts model performance.

We performed inference using our Bio+Discharge Summary BERT-based model on 9,522,262 notes for the 401,826 patients who had ≥1 eligible note within the 3 years prior to the start of follow-up. The distribution of notes used for inference was: inpatient or outpatient history and physical (*n* = 9,074,155, 95.3%) and discharge summary (*n* = 448,107, 4.7%). We then performed the following post-processing on NLP-extracted values:

1. Checked model identifications and extended identified tokens to include additional significant figures or unit tokens.
2. Harmonized model identifications into a single unit for each vital sign (i.e., kg for weight, cm for height, and mmHg for blood pressure) using a rule-based system to convert text patterns to numeric values.
3. Imposed physiological constraints for each feature, to ensure that each extraction was biologically plausible. The following constraints were used:
  - 91–305 cm for height
  - 20–450 kg for weight
  - 50–300 mmHg for systolic, and 20–200 mmHg for diastolic blood pressure
4. Filtered out “optimal weights” that appeared in notes in addition to patient weights (a common model failure mode) using a regular expression that discarded weight identifications that were followed by variations of the phrase “for BMI of 25”.

To assess the effect of each post-processing step on accuracy and yield, we performed an ablation study. A study cardiologist (SK) reviewed predicted vital sign identifications—weight, height, systolic, and diastolic blood pressure—after each post-processing step in an independent holdout set, producing an additional 400 validated vital sign values. The results from this analysis are presented in Supplementary Table 12. Supplementary Table 2 depicts the final NLP model yield and recovery rate for each vital sign. SK then performed a manual review of 50 randomly selected values for each vital sign (along with surrounding context from the note) obtained from an independent holdout set. For validation, references to prior values of the target vital sign (e.g., weight at last encounter) were adjudicated as correct. All 200 values reviewed accurately represented the true vital sign of interest. Since our primary goal was to ensure that the vital signs we extracted were accurate (rather than ensuring that every potential vital sign instance was detected), we did not validate negative examples. However, to qualitatively assess failure modes resulting in false negatives, SK reviewed 50 randomly sampled notes with no vital signs detected by NLP.

An analogous validation process was performed for the regular expression rule-based approach. We also compared the number of predicted values across models as a surrogate for overall yield. We ultimately ran inference with the NLP model on 9,522,262 notes for the 401,826 patients who had eligible notes in the 3 years prior to the start of cohort follow-up and utilized NLP values in our prediction models for individuals in whom baseline values were missing in the tabular data.

In addition to the primary agreement analyses between NLP and tabular vital sign values, we also assessed for potential temporal effects by assessing agreement stratified by year of extraction (Supplementary Fig 3). Furthermore, to better understand potential causes of discrepancies between tabular and NLP-based values, SK manually reviewed 80 pairs of tabular and NLP-extracted vital signs (20 each for height, weight, systolic blood pressure, and diastolic blood pressure), where each pair differed by a clinically meaningful amount (i.e., 6 cm for height, 5 kg for weight, 20 mmHg for systolic blood pressure, and 10 mmHg for diastolic blood pressure). We utilized NLP recovery in C3PO but not in the Convenience Samples.

### Statistical analysis

We tabulated the number of cardiac imaging studies, cardiac diagnostic tests, and unstructured text notes available within C3PO. We also cross-referenced the number of individuals in C3PO in whom genetic data are available for analysis through participation in the MGB Biobank biorepository. We assessed agreement between vital signs obtained using tabular data versus NLP by comparing sets of values obtained from each respective source on the same day within 3 years of the start of follow-up. We plotted paired values, calculated Pearson correlations, and assessed agreement using Bland–Altman plots. To estimate the magnitude of missing data reduction, we tabulated sample sizes for the PCE and CHARGE-AF analyses before and after NLP recovery.

We calculated the cumulative incidence of events at their respective time horizons (i.e., 5-year AF for CHARGE-AF, 10-year MI/stroke for PCE) using the Kaplan–Meier method. We also calculated incidence rates per 1000 person-years and corresponding Wald confidence intervals using the normal approximation. For longitudinal analyses, person-time ended at the earliest of an outcome event, death, last encounter of any type in the EHR, age 90, or the administrative censoring date for C3PO (August 31, 2019, Supplementary Fig 9).

The linear predictors of the CHARGE-AF^19^ and PCE scores^18^ were calculated using their published coefficients. The analysis set for each score was restricted to individuals without the disease of interest at baseline and within the published age range for each score (i.e., CHARGE-AF: 46–90 years, PCE: 40–79 years). For CHARGE-AF, the coefficient associated with the White race was attributed to White individuals, but not to individuals of other races^4,10,40^. Since dedicated PCE models are available only for White and Black individuals, as performed previously^41^ the models developed for Black individuals were utilized for individuals identifying as Black, while the models developed for White individuals were utilized for individuals of all other races. We performed a secondary analysis in which the White equations were deployed only among White individuals. Scores were converted into predicted event probabilities at their respective time horizons using their published equations.

We assessed model performance by fitting Cox proportional hazards models with the linear predictor of each model as the covariate of interest and tabulated the HR per 1-SD increase in score. Model discrimination was assessed using the inverse probability of censoring weighted c-index^42^. Model calibration was assessed in four ways: (1) visual inspection of predicted versus observed event rates within each decile of predicted risk (with corresponding fitted curves^43^), (2) the GND test, in which a greater chi-squared value and smaller *p*-value suggest miscalibration^44^, (3) calibration slope, where a value of one indicates optimal calibration^45^, and (4) the ICI, a measure of the average prediction error weighted by the empirical risk distribution^43^. We assessed calibration for the original models as well as after recalibration to the sample-level baseline hazard^20,45^. Confidence intervals for the ICI were obtained using bootstrapping (500–1000 iterations based on stratum sample size).

We plotted the cumulative risk of AF and MI/stroke according to level of predicted risk using CHARGE-AF and PCE, respectively. For these analyses, we used a threshold of <7.5% vs ≥7.5% for MI/stroke risk (the threshold used to determine candidacy for statin therapy in current American Heart Association/American College of Cardiology primary prevention guidelines^46^) and <2.5%, ≥2.5–5%, and ≥5% for AF risk (the thresholds used in the CHARGE-AF validation study^19^).

We repeated the analyses described above within the AF and MI/stroke Convenience Samples to compare the results of contrasting EHR sampling approaches. We assessed for differences in model calibration by comparing calibration slopes and ICI values using bootstrapping (500-1,000 iterations based on stratum sample size).

Analyses were performed using Python v3.8^47^ and R v4.0^48^. Two-sided *p*-values < 0.05 were considered statistically significant.

### Reporting Summary

Further information on research design is available in the Nature Research Reporting Summary linked to this article.

## Supplementary information

Supplemental Material

Reporting Summary Checklist

## Data Availability

MGB source data contain potentially identifying information, cannot be shared publicly, and is not available by application. Reasonable requests for collaboration will be considered on a case-by-case basis by direct correspondence with the authors.

## Code availability

The JEDI data processing pipeline underlying C3PO, as well as data processing scripts underlying the current analyses, are publicly available under a BSD-3 Clause license at https://github.com/broadinstitute/jedi-public.
