# Supplementary material for: Cohort design and natural language processing to reduce bias in electronic health records research
Source: NPJ Digit Med. 2022 Apr 8;5:47. doi: 10.1038/s41746-022-00590-0 (PMC8993873; doi:10.1038/s41746-022-00590-0)
Supplement: Supplementary file 1 — Supplemental Material [file 41746_2022_590_MOESM1_ESM.pdf]

Cohort Design and Natural Language Processing to Reduce Bias in Electronic Health Records Research

Supplementary Material

Index

Supplementary Table 1. Summary of available data types in C3PO 1

Supplementary Table 2. Vital sign-specific yield of NLP recovery 2

Supplementary Table 3. Comparative yield of regular expression rule-based approach vs deep natural language processing models for vital sign recovery 3

Supplementary Table 4. Performance of White PCE models with and without restriction to White individuals 4

Supplementary Table 5. Risk model performance in samples with and without NLP recovery 5

Supplementary Table 6. Current procedural terminology codes corresponding to office visits 7

Supplementary Table 7. Primary care algorithm validation results 8

Supplementary Table 8. PCE score components and weights 9

Supplementary Table 9. CHARGE-AF score components and weights 11

Supplementary Table 10. Clinical factor definitions 12

Supplementary Table 11. Yield of vital sign extractions using Bio+DischargeSummaryBERT trained for two versus five epochs 30

Supplementary Table 12. Ablation study of post-processing steps for NLP vital sign identifications 31

Supplementary Figure 1. Distribution of office visits and primary care office visits per person in C3PO versus Convenience Samples 32

Supplementary Figure 2. Example clinical note with NLP extracted vital signs 33

Supplementary Figure 3. Agreement between tabular and natural language processing-extracted vital signs by year of extraction 34

Supplementary Figure 4. Flow diagrams for C3PO MI/stroke and AF analysis samples 35

Supplementary Figure 5. Predicted event risk in C3PO PCE and CHARGE-AF analysis sets 36

Supplementary Figure 6. Cumulative risk of events stratified by predicted risk 37

Supplementary Figure 7. Calibration of original models in C3PO and Convenience Samples 38

Supplementary Figure 8. Calibration of recalibrated models in C3PO and Convenience Samples 40

Supplementary Figure 9. Longitudinal structure of C3PO 42

Supplementary Figure 10. Overlap between C3PO and MGH primary care registry 43

Supplementary Figure 11. MI/stroke and AF Convenience Sample construction 44

Supplementary Figure 12. Training and evaluation curves for fine-tuning Bio+Discharge Summary BERT for 5 epochs 45

Supplementary Figure 13. Training and evaluation curves for Bio+Discharge Summary BERT models across different sizes of training data 46

Supplementary References 47

Supplementary Table 1. Summary of available data types in C3PO

Modality	Prior to start of follow-up		Within 3 years prior to start of follow-up		During follow-up		Total (N, % of individuals)	
	**Total N**	**% of individuals with ≥ 1 study**	**Total N**	**% of individuals with ≥ 1 study**	**Total N**	**% of individuals with ≥ 1 study**	**Total N**	**% of individuals with ≥ 1 study**
Electrocardiograms	954,413	40.3	471,695	28.1	1,998,242	46.1	2,952,655	59.1
Echocardiograms	159,102	17.0	87,188	11.3	290,960	23.9	450,062	32.7
Cardiac magnetic resonance imaging	3,784	0.5	2,569	0.4	8,928	1.4	12,712	1.9
Narrative notes	17,285,956	93.9	11,766,067	92.4	59,360,809	97.0	76,646,765	99.5
Head computed tomography	110,387	11.9	56,704	6.7	212,508	17.3	322,895	24.8
Brain magnetic resonance imaging	86,776	8.7	47,452	5.6	170,176	15.0	256,952	20.8
Genetic data (through linkage to MGB Biobank)[1,2]							42,759	8.2

[1]Reported as number of individuals. For all other modalities, reported as number of tests.
[2]Includes all individuals enrolled in MGB Biobank. Although genotyping is planned in all individuals, it is available in 14,401 (2.8%) as of 03/2021.

Supplementary Table 2. Vital sign-specific yield of NLP recovery

Vital sign feature	Total NLP values extracted	# with NLP values	# with feature missing in tabular data*	# with ≥ 1 eligible note for feature recovery†	# with recovered feature	Eligible recovery rate (%)
Height	1,115,337	217,867	299,905	219,873	59,682	27.1%
Weight	2,749,471	310,687	204,049	143,655	76,298	53.1%
Systolic blood pressure	3,774,943	379,713	191,041	133,130	108,704	81.7%
Diastolic blood pressure	3,774,943	379,713	191,041	133,130	108,704	81.7%

[1]Missingness defined as no value included in the tabular data within 3 years prior to start of follow-up
[2]Eligible notes included discharge summaries, admission notes, and inpatient or outpatient progress notes, within 3 years prior to start of follow-up

Supplementary Table 3. Comparative yield of regular expression rule-based approach vs deep natural language processing models for vital sign recovery

Model	Blood Pressure		Weight		Height	
	Identifications	Individuals	Identifications	Individuals	Identifications	Individuals
Rule-Based Approach	3,227,971	373,378	2,254,571	301,049	1,065,252	215,797
BERT	3,683,182	378,044	2,319,347	301,145	1,034,839	208,307
Bio+Discharge Summary BERT	3,774,943	379,713	2,749,471	310,687	1,115,337	217,867
Positive predictive value of rule-based approach: Blood pressure 100%, weight 98%, height 100%<br>Positive predictive value of BERT: Blood pressure 100%, weight 96%, height 100%<br>Positive predictive value of Bio+Discharge Summary BERT: Blood pressure 100%, weight 100%, height 100%						

Supplementary Table 4. Performance of White PCE models with and without restriction to White individuals

Model	Hazard ratio (per 1-SD increase)	C-index	GND χ^2 [2]	Recalibrated GND χ^2 [3,4]	ICI[5]	Recalibrated ICI[4,5]	Calibration slope[6]
C3PO							
PCE (White women)[1]	2.50 (2.41-2.59)	0.768 (0.759-0.777)	412, p<0.01	1336, p<0.01	0.018 (0.016-0.020)	0.034 (0.032-0.036)	0.67 (0.65-0.70)
PCE (White and non-Black women)[1]	2.51 (2.43-2.59)	0.768 (0.760-0.775)	487, p<0.01	1689, p<0.01	0.018 (0.017-0.020)	0.034 (0.031-0.037)	0.67 (0.65-0.70)
PCE (White men)[1]	2.16 (2.09-2.24)	0.736 (0.727-0.746)	297, p<0.01	504, p<0.01	0.024 (0.021-0.027)	0.032 (0.029-0.035)	0.71 (0.68-0.74)
PCE (White and non-Black men)[1]	2.17 (2.11-2.24)	0.738 (0.730-0.746)	361, p<0.01	618, p<0.01	0.024 (0.022-0.027)	0.032 (0.029-0.035)	0.70 (0.68-0.73)

[1]PCE (White women): 3,442, 90,767, 7.2 (2.8, 10); PCE (White and non-Black women): 4,231, 107,998, 7.1 (2.8, 10); PCE (White men): 4,216, 63,945, 6.4 (2.3, 10); PCE (White and non-Black men): 4,928, 76,304, 6.2 (2.3, 10)
[2]C-index calculated using the inverse probability of censoring weighting method[1]
[3]Greenwood-Nam-D'Agostino (GND) test, a test of calibration.[2] Lower chi-squared values suggest better calibration (across equally-sized samples). Significant p-values indicate evidence of miscalibration.
[4]Values after recalibration to the baseline hazard of the sample (see text)
[5]Integrated calibration index, a quantitative measure of the average difference between predicted event risk and observed event incidence, weighted by the empirical distribution of event risk.[3] Smaller values indicate better calibration.
[6]A measure of calibration applicable to models that are calibrated-in-the-large.[4,5] A calibration slope equal to one is optimally calibrated.

Supplementary Table 5. Risk model performance in samples with and without NLP recovery

Model	N events	N total	Hazard ratio (per 1-SD increase)	C-index[3]	GND χ^2 [4]	Recalibrated GND χ^2 [4,5]	ICI[6]	Recalibrated ICI[5,6]	Calibration slope[7]
C3PO (without NLP recovery)									
PCE (White women)[1]	3,110	81,870	2.51 (2.41-2.60)	0.768 (0.759-0.776)	382, p<0.01	1319, p<0.01	0.019 (0.017-0.021)	0.036 (0.033-0.039)	0.66 (0.64-0.69)
PCE (Black women)[1]	447	6,631	2.42 (2.15-2.72)	0.729 (0.703-0.755)	47, p<0.01	185, p<0.01	0.029 (0.021-0.037)	0.056 (0.047-0.065)	0.61 (0.52-0.69)
PCE (White men)[1]	3,496	56,195	2.17 (2.09-2.25)	0.735 (0.726-0.745)	280, p<0.01	453, p<0.01	0.025 (0.022-0.028)	0.033 (0.030-0.036)	0.70 (0.66-0.73)
PCE (Black men)[1]	299	4,199	2.17 (1.92-2.44)	0.740 (0.709-0.771)	23, p<0.01	12, p=0.25	0.020 (0.0064-0.033)	0.0041 (0-0.019)	0.95 (0.81-1.09)
CHARGE-AF[1]	6,105	136,116	2.56 (2.50-2.62)	0.782 (0.776-0.788)	1366, p<0.01	1085, p<0.01	0.028 (0.027-0.030)	0.020 (0.019-0.021)	0.77 (0.75-0.79)
C3PO (with NLP recovery)									
PCE (White women)[2]	4,231	107,998	2.51 (2.43-2.59)	0.768 (0.760-0.775)	487, p<0.01	1689, p<0.01	0.018 (0.017-0.020)	0.034 (0.031-0.037)	0.67 (0.65-0.70)
PCE (Black women)[2]	617	8,450	2.39 (2.17-2.64)	0.724 (0.702-0.746)	69, p<0.01	257, p<0.01	0.030 (0.023-0.036)	0.057 (0.050-0.064)	0.60 (0.53-0.67)
PCE (White men)[2]	4,928	76,304	2.17 (2.11-2.24)	0.738 (0.730-0.746)	361, p<0.01	618, p<0.01	0.024 (0.022-0.027)	0.032 (0.029-0.035)	0.70 (0.68-0.73)
PCE (Black men)[2]	425	5,432	2.04 (1.85-2.25)	0.725 (0.698-0.751)	21, p=0.02	18, p=0.03	0.012 (0-0.025)	0.010 (0-0.024)	0.88 (0.77-1.00)
CHARGE-AF[2]	7,877	174,644	2.56 (2.50-2.61)	0.782 (0.777-0.787)	1856, p<0.01	1367, p<0.01	0.028 (0.027-0.030)	0.019 (0.018-0.021)	0.77 (0.75-0.79)

[1]PCE (White women): 6.7 (2.6, 10); PCE (Black women): 6.9 (2.6, 10); PCE (White men): 5.8 (2.2, 10); PCE (Black men): 6.1 (2.2, 10); CHARGE-AF: median follow-up, years (Q1,Q3): 5.0 (2.4,5.0)
[2]PCE (White women): 7.1 (2.8, 10); PCE (Black women): 7.7 (2.9, 10); PCE (White men): 6.2 (2.3, 10); PCE (Black men): 6.7 (2.5, 10); CHARGE-AF: median follow-up, years (Q1,Q3): 5.0 (2.3,5.0)
[3]C-index calculated using the inverse probability of censoring weighting method[1]
[4]Greenwood-Nam-D'Agostino (GND) test, a test of calibration.[2] Lower chi-squared values suggest better calibration (across equally-sized samples). Significant p-values indicate evidence of miscalibration.
[5]Values after recalibration to the baseline hazard of the sample (see text)
[6]Integrated calibration index, a quantitative measure of the average difference between predicted event risk and observed event incidence, weighted by the empirical distribution of event risk.[3] Smaller values indicate better calibration.
[7]A measure of calibration applicable to models that are calibrated-in-the-large.[4,5] A calibration slope equal to one is optimally calibrated.

Supplementary Table 6. Current procedural terminology codes corresponding to office visits

Code[1]	Description
C99058	Office services provided on an emergency basis
C99201	New patient visit, level 1
C99202	New patient visit, level 2
C99203	New patient visit, level 3
C99204	New patient visit, level 4
C99205	New patient visit, level 5
C99211	Established patient visit, level 1
C99212	Established patient visit, level 2
C99213	Established patient visit, level 3
C99214	Established patient visit, level 4
C99215	Established patient visit, level 5
CG0344	Initial preventive physical examination
CG0438	Annual wellness visit; initial
CG0439	Annual wellness visit; subsequent
CG0463	Hospital outpatient clinic visit

[1]Analogous institution-specific billing codes also included in the office visit definition

Supplementary Table 7. Primary care algorithm validation results

Sample	PPV (95% CI)	Raw Agreement	Kappa (95% CI)
Cases Tier 1 (n=200)[1]	69.5 (62.6-75.8)	59/60 (98.3%)	0.96 (0.9-1)
Cases Tier 2 (n=200)[2]	94.0 (89.8-96.7)	58/60 (96.7%)	0.78 (0.49-1)
Controls (n=200)	91.5 (86.7-95.0)	60/60 (100%)	1 (1-1)

[1]Defined as presence of directly verifiable primary care office visit on both qualifying dates (±7 days)
[2]Defined as presence of ≥2 directly verifiable primary care office visits within 1-3 consecutive years (C3PO temporal criteria), with allowance for misclassification of date

Supplementary Table 8. PCE score components and weights

Covariate	Coefficient (White)	Coefficient (Black)
Women		
Ln age (y)	-29.799	17.114
Ln age, squared	4.884	-
Ln total cholesterol (mg/dL)	13.540	0.940
Ln age x Ln total cholesterol	-3.114	-
Ln HDL-C (mg/dL)	-13.578	-18.920
Ln age x Ln HDL-C	3.149	4.475
Ln treated SBP (mmHg)	2.019	29.291
Ln age x Ln treated SBP	-	-6.432
Ln untreated SBP (mmHg)	1.957	27.820
Ln age x Ln untreated SBP	-	-6.087
Current smoker	7.574	0.691
Ln age x Current Smoker	-1.665	-
Diabetes	0.661	0.874
Men		
Ln age (y)	12.344	2.469
Ln total cholesterol (mg/dL)	11.853	0.302
Ln age x Ln total cholesterol	-2.664	-
Ln HDL-C (mg/dL)	-7.990	-0.307
Ln age x Ln HDL-C	1.769	-
Ln treated SBP (mmHg)	1.797	1.916
Ln untreated SBP (mmHg)	1.764	1.809
Current smoker	7.837	0.549

Ln age x Current smoker	-1.795	-
Diabetes	0.658	0.645
HDL-C = high density lipoprotein cholesterol; Ln = natural logarithm; SBP = systolic blood pressure		

Supplementary Table 9. CHARGE-AF score components and weights

Covariate	Coefficient
Age (per 5-year increase), y	0.508
Race (white)	0.465
Height (per 10 cm increase), cm	0.248
Weight (per 15 kg increase), kg	0.115
Systolic blood pressure (per 20 mmHg increase), mmHg	0.197
Diastolic blood pressure (per 10 mmHg increase), mmHg	-0.101
Current smoking	0.359
Anti-hypertensive medication use	0.349
Diabetes	0.237
Heart failure	0.701
Myocardial infarction	0.496

Supplementary Table 10. Clinical factor definitions

Phenotype	Code type	Data codes	Data code definitions
Atrial Fibrillation	ICD9	427.3, 427.31, 427.32, 99.61	Atrial fibrillation and flutter, atrial fibrillation, atrial flutter, atrial cardioversion
Atrial Fibrillation	ICD10	I48.0, I48.1, I48.2, I48.3, I48.4, I48.91, I48.92	Paroxysmal atrial fibrillation, Persistent atrial fibrillation, Chronic atrial fibrillation, Typical atrial flutter, Atypical atrial flutter, Unspecified atrial fibrillation, Unspecified atrial flutter
Atrial Fibrillation	CPT	33253, 33257, 33259, 92960, 92961, 93650, 93656, 93657	Operative incisions and reconstruction of atria for treatment of atrial fibrillation or atrial flutter (eg, maze procedure), Operative tissue ablation and reconstruction of atria, performed at the time of other cardiac procedure(s), limited (eg, modified maze procedure), Operative tissue ablation and reconstruction of atria, performed at the time of other cardiac procedure(s), extensive (eg, maze procedure), with cardiopulmonary bypass (List separately in addition to code for primary procedure), Cardioversion, elective, electrical conversion of arrhythmia; external, Cardioversion, elective, electrical conversion of arrhythmia; internal (separate procedure), Intracardiac catheter ablation of atrioventricular node function, atrioventricular conduction for creation of complete heart block, with or without temporary pacemaker placement, Comprehensive electrophysiologic evaluation, including transseptal catheterizations, insertion and repositioning of multiple electrode catheters with induction or attempted induction of an arrhythmia with atrial recording and pacing, when possible, right ventricular pacing and recording, His bundle recording with intracardiac catheter ablation of arrhythmogenic focus, with treatment of atrial fibrillation by ablation by pulmonary vein isolation, Additional linear or focal intracardiac catheter ablation of the left or right atrium for treatment of atrial fibrillation remaining after completion of pulmonary vein isolation
Diabetes	ICD9	249, 249.01, 249.1, 249.11, 249.2, 249.21, 249.3, 249.31, 249.4, 249.41, 249.5, 249.51, 249.6, 249.61, 249.7, 249.71, 249.8, 249.81, 249.9, 249.91, 250, 250.01, 250.02, 250.03, 250.1, 250.11, 250.12, 250.13, 250.2, 250.21, 250.22, 250.23, 250.3, 250.31, 250.32, 250.33, 250.4, 250.41, 250.42,	Secondary diabetes mellitus without mention of complication, not stated as uncontrolled, or unspecified, Secondary diabetes mellitus without mention of complication, uncontrolled, Secondary diabetes mellitus with ketoacidosis, not stated as uncontrolled, or unspecified, Secondary diabetes mellitus with ketoacidosis, uncontrolled, Secondary diabetes mellitus with hyperosmolarity, not stated as uncontrolled, or unspecified, Secondary diabetes mellitus with hyperosmolarity, uncontrolled, Secondary diabetes mellitus with other coma, not stated as uncontrolled, or unspecified, Secondary diabetes mellitus with other coma, uncontrolled, Secondary diabetes mellitus with renal manifestations, not stated as uncontrolled, or unspecified, Secondary diabetes mellitus with renal manifestations, uncontrolled, Secondary diabetes mellitus with ophthalmic manifestations, not stated as uncontrolled, or unspecified, Secondary diabetes mellitus with ophthalmic manifestations, uncontrolled, Secondary diabetes mellitus with neurological manifestations, not stated as uncontrolled, or unspecified, Secondary diabetes mellitus with neurological manifestations, uncontrolled, Secondary diabetes mellitus with peripheral circulatory disorders, not stated as uncontrolled, or unspecified, Secondary diabetes mellitus with peripheral circulatory disorders, uncontrolled,

		250.43, 250.5, 250.51, 250.52, 250.53, 250.6, 250.61, 250.62, 250.63, 250.7, 250.71, 250.72, 250.73, 250.8, 250.81, 250.82, 250.83, 250.9, 250.91, 250.92, 250.93, 357.2, 362.01, 362.02, 362.03, 362.04, 362.05, 362.06, 362.07, 366.41, 791.6	Secondary diabetes mellitus with other specified manifestations, not stated as uncontrolled, or unspecified, Secondary diabetes mellitus with other specified manifestations, uncontrolled, Secondary diabetes mellitus with unspecified complication, not stated as uncontrolled, or unspecified, Secondary diabetes mellitus with unspecified complication, uncontrolled, Diabetes mellitus without mention of complication, type II or unspecified type, not stated as uncontrolled, Diabetes mellitus without mention of complication, type I [juvenile type], not stated as uncontrolled, Diabetes mellitus without mention of complication, type II or unspecified type, uncontrolled, Diabetes mellitus without mention of complication, type I [juvenile type], uncontrolled, Diabetes with ketoacidosis, type II or unspecified type, not stated as uncontrolled, Diabetes with ketoacidosis, type I [juvenile type], not stated as uncontrolled, Diabetes with ketoacidosis, type II or unspecified type, uncontrolled, Diabetes with ketoacidosis, type I [juvenile type], uncontrolled, Diabetes with hyperosmolarity, type II or unspecified type, not stated as uncontrolled, Diabetes with hyperosmolarity, type I [juvenile type], not stated as uncontrolled, Diabetes with hyperosmolarity, type II or unspecified type, uncontrolled, Diabetes with hyperosmolarity, type I [juvenile type], uncontrolled, Diabetes with other coma, type II or unspecified type, not stated as uncontrolled, Diabetes with other coma, type I [juvenile type], not stated as uncontrolled, Diabetes with other coma, type II or unspecified type, uncontrolled, Diabetes with other coma, type I [juvenile type], uncontrolled, Diabetes with renal manifestations, type II or unspecified type, not stated as uncontrolled, Diabetes with renal manifestations, type I [juvenile type], not stated as uncontrolled, Diabetes with renal manifestations, type II or unspecified type, uncontrolled, Diabetes with renal manifestations, type I [juvenile type], uncontrolled, Diabetes with ophthalmic manifestations, type II or unspecified type, not stated as uncontrolled, Diabetes with ophthalmic manifestations, type I [juvenile type], not stated as uncontrolled, Diabetes with ophthalmic manifestations, type II or unspecified type, uncontrolled, Diabetes with ophthalmic manifestations, type I [juvenile type], uncontrolled, Diabetes with neurological manifestations, type II or unspecified type, not stated as uncontrolled, Diabetes with neurological manifestations, type I [juvenile type], not stated as uncontrolled, Diabetes with neurological manifestations, type II or unspecified type, uncontrolled, Diabetes with neurological manifestations, type I [juvenile type], uncontrolled, Diabetes with peripheral circulatory disorders, type II or unspecified type, not stated as uncontrolled, Diabetes with peripheral circulatory disorders, type I [juvenile type], not stated as uncontrolled, Diabetes with peripheral circulatory disorders, type II or unspecified type, uncontrolled, Diabetes with peripheral circulatory disorders, type I [juvenile type], uncontrolled, Diabetes with other specified manifestations, type II or unspecified type, not stated as uncontrolled, Diabetes with other specified manifestations, type I [juvenile type], not stated as uncontrolled, Diabetes with other specified manifestations, type II or unspecified type, uncontrolled, Diabetes with other specified manifestations, type I [juvenile type], uncontrolled, Diabetes with unspecified complication, type II or unspecified type, not stated as uncontrolled, Diabetes with unspecified complication, type I [juvenile type], not stated as uncontrolled, Diabetes with unspecified complication, type II or unspecified type, uncontrolled, Diabetes with unspecified complication, type I [juvenile type], uncontrolled, Polyneuropathy in diabetes, Background diabetic retinopathy, Proliferative diabetic retinopathy, Nonproliferative diabetic retinopathy NOS, Mild nonproliferative diabetic retinopathy, Moderate nonproliferative diabetic retinopathy, Severe nonproliferative diabetic retinopathy, Diabetic macular edema, Diabetic cataract, Acetonuria

Diabetes	ICD10	E08.00, E08.01, E08.10, E08.11, E08.21, E08.22, E08.29, E08.311, E08.319, E08.321, E08.329, E08.331, E08.339, E08.341, E08.349, E08.351, E08.359, E08.36, E08.39, E08.40, E08.41, E08.42, E08.43, E08.44, E08.49, E08.51, E08.52, E08.610, E08.618, E08.620, E08.621, E08.622, E08.628, E08.630, E08.638, E08.641, E08.649, E08.65, E08.69, E08.8, E08.9, E08.90, E09.00, E09.01, E09.10, E09.11, E09.21, E09.22, E09.29, E09.311, E09.319, E09.321, E09.329, E09.331, E09.339, E09.341, E09.349, E09.351, E09.359, E09.36, E09.39, E09.40, E09.41, E09.42, E09.43, E09.44, E09.49, E09.51, E09.52, E09.59, E09.610, E09.618, E09.620, E09.621, E09.622, E09.628, E09.630, E09.638, E09.641, E09.649, E09.65, E09.69, E09.8, E09.9, E10.10, E10.11, E10.21, E10.22, E10.29, E10.311, E10.319, E10.321, E10.329,	Diabetes Mellitus Due To Underlying Condition With Hyperosmolarity without nonketotic hyperglycemic-hyperosmolar coma (NKHHC), Diabetes Mellitus Due To Underlying Condition With Hyperosmolarity With Coma, Diabetes Mellitus Due To Underlying Condition With Ketoacidosis Without Coma, Diabetes Mellitus Due To Underlying Condition With Ketoacidosis With Coma, Diabetes Mellitus Due To Underlying Condition With Diabetic Nephropathy, Diabetes Mellitus Due To Underlying Condition With Diabetic chronic kidney disease, Diabetes Mellitus Due to underlying condition with other diabetic kidney complication, Diabetes Mellitus Due To Underlying Condition With Unspecified Diabetic Retinopathy With Macular Edema, Diabetes Mellitus Due To Underlying Condition With Unspecified Diabetic Retinopathy Without Macular Edema, Diabetes Mellitus due to underlying condition with mild nonproliferative diabetic retinopathy with macular edema, Diabetes mellitus due to underlying condition with mild nonproliferative diabetic retinopathy without macular edema, Diabetes mellitus due to underlying condition with moderate nonproliferative diabetic retinopathy with macular edema, Diabetes mellitus due to underlying condition with moderate nonproliferative diabetic retinopathy without macular edema, Diabetes mellitus due to underlying condition with severe nonproliferative diabetic retinopathy with macular edema, Diabetes mellitus due to underlying condition with severe nonproliferative diabetic retinopathy without macular edema, Diabetes mellitus due to underlying condition with proliferative diabetic retinopathy with macular edema, Diabetes mellitus due to underlying condition with proliferative diabetic retinopathy without macular edema, Diabetes Mellitus Due To Underlying Condition With Diabetic Cataract, Diabetes Mellitus Due To Underlying Condition With Other Diabetic Ophthalmic Complication, Diabetes Mellitus Due To Underlying Condition With Diabetic Neuropathy, Unspecified, Diabetes Mellitus Due To Underlying Condition With Diabetic Mononeuropathy, Diabetes Mellitus Due To Underlying Condition With Diabetic Polyneuropathy, Diabetes Mellitus Due To Underlying Condition With Diabetic Autonomic (Poly)Neuropathy, Diabetes Mellitus Due To Underlying Condition With Diabetic Amyotrophy, Diabetes Mellitus Due To Underlying Condition With Other Diabetic Neurological Complication, Diabetes Mellitus Due To Underlying Condition With Diabetic Peripheral Angiopathy Without Gangrene, Diabetes Mellitus Due To Underlying Condition With Diabetic Neuropathic Arthropathy, Diabetes Mellitus Due To Underlying Condition with diabetic neuropathic arthropathy, Diabetes Mellitus Due To Underlying Condition With Diabetic arthropathy, Diabetes Mellitus Due To Underlying Condition With diabetic dermatitis, Diabetes Mellitus Due To Underlying Condition With Foot Ulcer, Diabetes Mellitus Due To Underlying Condition With Other Skin ulcer, Diabetes Mellitus Due To Underlying Condition With other skin complications, Diabetes Mellitus Due To Underlying Condition With periodontal disease, Diabetes Mellitus Due To Underlying Condition With other oral complications, Diabetes Mellitus Due To Underlying Condition With hypoglycemia with coma, Diabetes mellitus due to underlying condition with hypoglycemia without coma, Diabetes Mellitus Due To Underlying Condition With Hyperglycemia, Diabetes Mellitus Due To Underlying Condition With Other Specified Complication, Diabetes Mellitus Due To Underlying Condition With Unspecified Complications, Diabetes mellitus due to underlying condition without Complications, Diabetes Mellitus Due To Underlying Condition Without Complications, Drug or Chemical induced diabetes mellitus with hyperosmolarity without nonketotic hyperglycemic-hyperosmolar coma (NKHHC), Drug Or Chemical Induced Diabetes Mellitus With Hyperosmolarity With Coma, Drug Or Chemical Induced Diabetes Mellitus With Ketoacidosis Without Coma, Drug Or Chemical Induced Diabetes

		E10.331, E10.339,<br>E10.341, E10.349,<br>E10.351, E10.359,<br>E10.36, E10.39, E10.40,<br>E10.41, E10.42, E10.43,<br>E10.44, E10.49, E10.51,<br>E10.52, E10.59,<br>E10.610, E10.618,<br>E10.620, E10.621,<br>E10.622, E10.628,<br>E10.630, E10.638,<br>E10.641, E10.649,<br>E10.65, E10.69, E10.8,<br>E10.9, E11.00, E11.01,<br>E11.21, E11.22, E11.29,<br>E11.311, E11.319,<br>E11.321, E11.329,<br>E11.331, E11.339,<br>E11.341, E11.349,<br>E11.351, E11.359,<br>E11.36, E11.39, E11.40,<br>E11.41, E11.42, E11.51,<br>E11.52, E11.59,<br>E11.610, E11.618,<br>E11.620, E11.621,<br>E11.622, E11.628,<br>E11.630, E11.638,<br>E11.641, E11.649,<br>E11.65, E11.69, E11.8,<br>E11.9, E13.00, E13.01,<br>E13.10, E13.11, E13.21,<br>E13.22, E13.29,<br>E13.311, E13.319,<br>E13.321, E13.329,<br>E13.331, E13.339,<br>E13.341, E13.349,<br>E13.351, E13.359,<br>E13.36, E13.39, E13.40,<br>E13.41, E13.42, E13.43,	Mellitus With Ketoacidosis With Coma, Drug Or Chemical Induced Diabetes Mellitus With Diabetic Nephropathy, Drug Or Chemical Induced Diabetes Mellitus With diabetic chronic kidney disease, Drug or chemical induced diabetes mellitus with other diabetic kidney complication, Drug Or Chemical Induced Diabetes Mellitus With Unspecified Diabetic Retinopathy With Macular Edema, Drug Or Chemical Induced Diabetes Mellitus With Unspecified Diabetic Retinopathy Without Macular Edema, Drug or chemical induced diabetes mellitus with mild nonproliferative diabetic retinopathy with macular edema, Drug or chemical induced diabetes mellitus with mild nonproliferative diabetic retinopathy without macular edema, Drug or chemical induced diabetes mellitus with moderate nonproliferative diabetic retinopathy with macular edema, Drug or chemical induced diabetes mellitus with moderate nonproliferative diabetic retinopathy without macular edema, Drug or chemical induced diabetes mellitus with severe nonproliferative diabetic retinopathy with macular edema, Drug or chemical induced diabetes mellitus with severe nonproliferative diabetic retinopathy without macular edema, Drug or chemical induced diabetes mellitus with proliferative diabetic retinopathy with macular edema, Drug or chemical induced diabetes mellitus with proliferative diabetic retinopathy without macular edema, Drug Or Chemical Induced Diabetes Mellitus With Diabetic Cataract, Drug Or Chemical Induced Diabetes Mellitus With Other Diabetic Ophthalmic Complication, Drug Or Chemical Induced Diabetes Mellitus With Neurological Complications With Diabetic Neuropathy, Unspecified, Drug Or Chemical Induced Diabetes Mellitus With Neurological Complications With Diabetic Mononeuropathy, Drug Or Chemical Induced Diabetes Mellitus With Neurological Complications With Diabetic Polyneuropathy, Drug Or Chemical Induced Diabetes Mellitus With Neurological Complications With Diabetic Autonomic (Poly)Neuropathy, Drug Or Chemical Induced Diabetes Mellitus With Neurological Complications With Diabetic Amyotrophy, Drug Or Chemical Induced Diabetes Mellitus With Neurological Complications With Other Diabetic Neurological Complication, Drug Or Chemical Induced Diabetes Mellitus With Diabetic Peripheral Angiopathy Without Gangrene, Drug Or Chemical Induced Diabetes Mellitus With Diabetic peripheral angiopathy with gangrene, Drug or chemical induced diabetes mellitus with other circulatory complications, Drug Or Chemical Induced Diabetes Mellitus With Diabetic neuropathic Arthropathy, Drug or chemical induced diabetes mellitus with other diabetic arthropathy, Drug Or Chemical Induced Diabetes Mellitus With diabetic dermatitis, Drug Or Chemical Induced Diabetes Mellitus With foot ulcer, Drug Or Chemical Induced Diabetes Mellitus With Other Skin ulcer, Drug Or Chemical Induced Diabetes Mellitus With other skin complications, Drug Or Chemical Induced Diabetes Mellitus With periodontal disease, Drug Or Chemical Induced Diabetes Mellitus With other oral complications, Drug Or Chemical Induced Diabetes Mellitus With Hypoglycemia with coma, Drug Or Chemical Induced Diabetes Mellitus With hypoglycemia without coma, Drug Or Chemical Induced Diabetes Mellitus With Hyperglycemia, Drug Or Chemical Induced Diabetes Mellitus With Other Specified Complication, Drug Or Chemical Induced Diabetes Mellitus With Unspecified Complications, Drug Or Chemical Induced Diabetes Mellitus without complications, Type 1 Diabetes Mellitus With Ketoacidosis Without Coma, Type 1 Diabetes Mellitus With Ketoacidosis With Coma, Type 1 Diabetes Mellitus With Diabetic Nephropathy, Type 1 Diabetes Mellitus With diabetic chronic kidney disease, Type 1 Diabetes Mellitus With other diabetic kidney complication, Type 1 Diabetes Mellitus With Unspecified Diabetic Retinopathy With Macular Edema, Type 1 Diabetes Mellitus With Unspecified Diabetic Retinopathy Without Macular Edema, Type 1 Diabetes mellitus

		E13.44, E13.49, E13.51, E13.52, E13.59, E13.610, E13.618, E13.620, E13.621, E13.622, E13.628, E13.630, E13.638, E13.641, E13.649, E13.65, E13.69, E13.8, E13.9, R82.4	with mild nonproliferative diabetic retinopathy with macular edema, Type 1 Diabetes mellitus with mild nonproliferative diabetic retinopathy without macular edema, Type 1 diabetes mellitus with moderate nonproliferative diabetic retinopathy with macular edema, Type 1 diabetes mellitus with moderate nonproliferative diabetic retinopathy without macular edema, Type 1 diabetes mellitus with severe nonproliferative diabetic retinopathy with macular edema, Type 1 diabetes mellitus with severe nonproliferative diabetic retinopathy without macular edema, Type 1 diabetes mellitus with proliferative diabetic retinopathy with macular edema, Type 1 diabetes mellitus with proliferative diabetic retinopathy without macular edema, Type 1 Diabetes Mellitus With Diabetic Cataract, Type 1 Diabetes Mellitus With Other Diabetic Ophthalmic Complication, Type 1 Diabetes Mellitus With Diabetic Neuropathy, Unspecified, Type 1 Diabetes Mellitus With diabetic mononeuropathy, Type 1 Diabetes Mellitus With Diabetic polyneuropathy, Type 1 Diabetes mellitus with diabetic autonomic (poly)neuropathy, Type 1 diabetes mellitus with diabetic amyotrophy, Type 1 diabetes mellitus with other diabetic neurological complication, Type 1 Diabetes Mellitus With Diabetic Peripheral Angiopathy Without Gangrene, Type 1 diabetes mellitus with diabetic peripheral angiopathy with gangrene, Type 1 diabetes mellitus with other circulatory complications, Type 1 diabetes mellitus with diabetic neuropathic arthropathy, Type 1 Diabetes Mellitus With Diabetic arthropathy, Type 1 Diabetes Mellitus With diabetic dermatitis, Type 1 Diabetes Mellitus With Other foot ulcer, Type 1 Diabetes Mellitus With Other Skin ulcer, Type 1 Diabetes Mellitus With other skin complications, Type 1 Diabetes Mellitus With periodontal disease, Type 1 Diabetes Mellitus With other oral complications, Type 1 Diabetes Mellitus With Hypoglycemia With Coma, Type 1 Diabetes Mellitus With Hypoglycemia without coma, Type 1 Diabetes Mellitus With Hyperglycemia, Type 1 Diabetes Mellitus With Other Specified Complication, Type 1 Diabetes Mellitus With Unspecified Complications, Type 1 Diabetes Mellitus Without Complications, Type 2 Diabetes Mellitus With Hyperosmolarity Without Nonketotic Hyperglycemic-Hyperosmolar Coma (Nkhhc), Type 2 Diabetes Mellitus With Hyperosmolarity With Coma, Type 2 diabetes mellitus with diabetic nephropathy, Type 2 diabetes mellitus with diabetic chronic kidney disease, Type 2 diabetes mellitus with other diabetic kidney complication, Type 2 Diabetes Mellitus With Unspecified Diabetic Retinopathy With Macular Edema, Type 2 Diabetes Mellitus With Unspecified Diabetic Retinopathy Without Macular Edema, Type 2 Diabetes Mellitus With Mild Nonproliferative Diabetic Retinopathy With Macular Edema, Type 2 Diabetes Mellitus With Mild Nonproliferative Diabetic Retinopathy Without Macular Edema, Type 2 Diabetes Mellitus With Moderate Nonproliferative Diabetic Retinopathy With Macular Edema, Type 2 Diabetes Mellitus with moderate nonproliferative diabetic retinopathy Without Macular Edema, Type 2 Diabetes Mellitus With Severe Nonproliferative Diabetic Retinopathy With Macular Edema, Type 2 Diabetes Mellitus With severe nonproliferative Diabetic Retinopathy Without Macular Edema, Type 2 Diabetes Mellitus with proliferative diabetic retinopathy with macular edema, Type 2 Diabetes Mellitus With proliferative diabetic retinopathy without macular edema, Type 2 Diabetes Mellitus With Diabetic Cataract, Type 2 Diabetes Mellitus With Other Diabetic Ophthalmic Complication, Type 2 Diabetes Mellitus With Diabetic Neuropathy, Unspecified, Type 2 Diabetes Mellitus With Diabetic mononeuropathy, Type 2 Diabetes Mellitus with diabetic polyneuropathy, Type 2 Diabetes Mellitus With Diabetic Peripheral Angiopathy Without Gangrene, Type 2 Diabetes Mellitus With diabetic peripheral Angiopathy With Gangrene, Type 2 Diabetes Mellitus with other circulatory complications, Type 2 diabetes

			mellitus with diabetic neuropathic arthropathy, Type 2 diabetes mellitus with other diabetic arthropathy, Type 2 Diabetes Mellitus with diabetic dermatitis, Type 2 Diabetes Mellitus With foot ulcer, Type 2 Diabetes Mellitus With Other Skin ulcer, Type 2 Diabetes Mellitus With other skin complications, Type 2 Diabetes Mellitus With periodontal disease, Type 2 Diabetes Mellitus With other oral complications, Type 2 Diabetes Mellitus With Hypoglycemia With Coma, Type 2 Diabetes Mellitus With Hypoglycemia without coma, Type 2 Diabetes Mellitus With Hyperglycemia, Type 2 Diabetes Mellitus With Other Specified Complication, Type 2 Diabetes Mellitus With Unspecified Complications, Type 2 Diabetes Mellitus Without Complications, Other specified diabetes mellitus with hyperosmolarity without nonketotic hyperglycemic-hyperosmolar coma (NKHHC), Other specified diabetes mellitus with hyperosmolarity with coma, Other specified diabetes mellitus with ketoacidosis without coma, Other specified diabetes mellitus with ketoacidosis with coma, Other specified diabetes mellitus with diabetic nephropathy, Other specified diabetes mellitus with diabetic chronic kidney disease, Other specified diabetes mellitus with other diabetic kidney complication, Other specified diabetes mellitus with unspecified diabetic retinopathy with macular edema, Other specified diabetes mellitus with unspecified diabetic retinopathy without macular edema, Other specified diabetes mellitus with mild nonproliferative diabetic retinopathy with macular edema, Other specified diabetes mellitus with mild nonproliferative diabetic retinopathy without macular edema, Other specified diabetes mellitus with moderate nonproliferative diabetic retinopathy with macular edema, Other specified diabetes mellitus with moderate nonproliferative diabetic retinopathy without macular edema, Other specified diabetes mellitus with severe nonproliferative diabetic retinopathy with macular edema, Other specified diabetes mellitus with severe nonproliferative diabetic retinopathy without macular edema, Other specified diabetes mellitus with proliferative diabetic retinopathy with macular edema, Other specified diabetes mellitus with proliferative diabetic retinopathy without macular edema, Other Specified Diabetes Mellitus With diabetic cataract, Other Specified Diabetes Mellitus With other diabetic ophthalmic complication, Other Specified Diabetes Mellitus With Diabetic neuropathy, unspecified, Other Specified Diabetes Mellitus With Diabetic mononeuropathy, Other Specified Diabetes Mellitus With Diabetic Polyneuropathy, Other Specified Diabetes Mellitus With Diabetic Autonomic (Poly)Neuropathy, Other Specified Diabetes Mellitus With Diabetic Amyotrophy, Other Specified Diabetes Mellitus With Other Diabetic Neurological Complication, Other specified diabetes mellitus with diabetic peripheral angiopathy without gangrene, Other specified diabetes mellitus with diabetic peripheral angiopathy with gangrene, Other Specified Diabetes Mellitus With Other Circulatory Complications, Other specified diabetes mellitus with diabetic neuropathic arthropathy, Other specified diabetes mellitus with other diabetic arthropathy, Other Specified Diabetes Mellitus With Diabetic Dermatitis, Other Specified Diabetes Mellitus With Foot Ulcer, Other Specified Diabetes Mellitus With Other Skin Ulcer, Other specified diabetes mellitus with other skin complications, Other specified diabetes mellitus with periodontal disease, Other specified diabetes mellitus with other oral complications, Other Specified Diabetes Mellitus With Hypoglycemia With Coma, Other Specified Diabetes Mellitus With Hypoglycemia Without Coma, Other Specified Diabetes Mellitus With Hyperglycemia, Other Specified Diabetes Mellitus With Other Specified Complication, Other Specified Diabetes Mellitus With Unspecified Complications, Other Specified Diabetes Mellitus Without Complications, Acetonuria

Diabetes	Med	metformin, glucophage, riomet, fortamet, glumetzia, chlorpropamide, glimepiride, glyburide, glipizide, tolazamide, tolbutamide, diabinese, amaryl, diabeta, micronase, glucotrol, glynase, tolinase, orinase, tolbutamide, repaglinide, nateglinide, prandin, starlix, pioglitazone, rosiglitazone, actos, avandia, sitagliptin, saxagliptin, linagliptin, alogliptin, januvia, onglyza, tradjenta, nesina, acarbose, miglitol, precose, glyset, pramlintide, symlin, liraglutide, exenatide, albiglutide, dulaglutide, victoza, bydureon, byetta, tanzeum, trulicity, canagliflozin, dapagliflozin, empagliflozin, invokana, farxiga, jardiance, actoplus, glucovance, metaglip, janumet, kombiglyze, prandimet, duetact, kazano, invokamet, xigduo, synjardy, jentadueto, avandamet, oseni, glyxambi, avandaryl, juvisync, glargine,	Biguanides, Sulfonylureas, Meglitinides, Thiazolidinediones, DPP-4 Inhibitors, Alpha-glucosidase inhibitors, Amylin analogue, GLP-1 Agonist, SGLT2 Inhibitor, Combination pills, long-actin insulin

		basaglar, lantus, toujeo, detemir, degludec, levemir, tresiba	
Hyperlipidemia	ICD9	272, 272.1, 272.2, 272.3, 272.4, 272.5, 272.6, 272.7, 272.8, 272.9, 759.9	Pure hypercholesterolemia, Pure hyperglyceridemia, Mixed hyperlipidemia, Hyperchylomicronemia, Other hyperlipidemia, Hyperlipidemia, unspecified, Lipoprotein deficiency, Lipidoses, Other specified metabolic disorders, Disorder of lipoprotein metabolism, unspecified, Disorders of bile acid and cholesterol metabolism
Hyperlipidemia	ICD10	E71.30, E75.21, E75.22, E75.5, E75.6, E77.0, E77.1, E78.0, E78.1, E78.2, E78.3, E78.4, E78.5, E78.6, E78.7, E78.70, E78.79, E78.81, E78.89, E78.9, E88.1, E88.89	Disorder of fatty-acid metabolism, unspecified, Lipidoses, Lipidoses, Other lipid storage disorders, Lipid storage disorder, unspecified, Lipidoses, Lipidoses, Pure hypercholesterolemia, Pure hyperglyceridemia, Mixed hyperlipidemia, Hyperchylomicronemia, Other hyperlipidemia, Hyperlipidemia, unspecified, Lipoprotein deficiency, Disorders of bile acid and cholesterol metabolism, Disorder of bile acid and cholesterol metabolism, unspecified, Other disorders of bile acid and cholesterol metabolism, Lipid dermatoarthritis, Other lipoprotein metabolism disorders, Disorder of lipoprotein metabolism, unspecified, Lipodystrophy, Other specified metabolic disorders
Hyperlipidemia	Med	advicor, alirocumab, altoprev, antara, atorvastatin, cholestyramine, colesevelam, colestid, colestipol, crestor, evolocumab, ezetimibe, fenofibrate, fenofibric acid, fluvastatin, gemfibrozil, juxtapid, kynamro, lescol, lipitor, livalo, lomitapide, lopid, lovastatin, mipomersen, niacin, niacor, niaspan, nicotinic acid, pitavastatin, praluent, pravastatin, prevachol, prevalite, repatha, rosuvastatin, simvastatin, tricor,	

		triglide, trilipix, vytorin, welchol, zetia, zocor	
Heart Failure	ICD9	398.91, 402.01, 402.11, 402.91, 404.01, 404.03, 404.11, 404.13, 404.91, 404.93, 428, 428.1, 428.2, 428.21, 428.22, 428.23, 428.3, 428.31, 428.32, 428.33, 428.4, 428.41, 428.42, 428.43, 428.9	Rheumatic heart failure (congestive), Malignant hypertensive heart disease with heart failure, Benign hypertensive heart disease with heart failure, Unspecified hypertensive heart disease with heart failure , Hypertensive heart and chronic kidney disease, malignant, with heart failure and with chronic kidney disease stage I through stage IV, or unspecified, Hypertensive heart and chronic kidney disease, malignant, with heart failure and with chronic kidney disease stage V or end stage renal disease, Hypertensive heart and chronic kidney disease, benign, with heart failure and with chronic kidney disease stage I through stage IV, or unspecified, Hypertensive heart and chronic kidney disease, benign, with heart failure and chronic kidney disease stage V or end stage renal disease, Hypertensive heart and chronic kidney disease, unspecified, with heart failure and with chronic kidney disease stage I through stage IV, or unspecified, Hypertensive heart and chronic kidney disease, unspecified, with heart failure and chronic kidney disease stage V or end stage renal disease, Congestive heart failure, unspecified, Left heart failure, Systolic heart failure, unspecified, Acute systolic heart failure, Chronic systolic heart failure, Acute on chronic systolic heart failure, Diastolic heart failure, unspecified, Acute diastolic heart failure, Chronic diastolic heart failure, Acute on chronic diastolic heart failure, Combined systolic and diastolic heart failure, unspecified, Acute combined systolic and diastolic heart failure, Chronic combined systolic and diastolic heart failure, Acute on chronic combined systolic and diastolic heart failure, Heart failure, unspecified
Heart Failure	ICD10	I09.81, I11.0, I13.0, I13.2, I50.1, I50.20, I50.21, I50.22, I50.23, I50.30, I50.31, I50.32, I50.33, I50.40, I50.41, I50.42, I50.43, I50.9, I97.130, I97.131	Rheumatic Heart Failure, Hypertensive Heart Disease With Heart Failure, Hypertensive Heart And Chronic Kidney Disease With Heart Failure And Stage 1 Through Stage 4 Chronic Kidney Disease, Or Unspecified Chronic Kidney Disease, Hypertensive Heart And Chronic Kidney Disease With Heart Failure And With Stage 5 Chronic Kidney Disease, Or End Stage Renal Disease, Left Ventricular Failure, Unspecified Systolic (Congestive) Heart Failure, Acute Systolic (Congestive) Heart Failure, Chronic Systolic (Congestive) Heart Failure, Acute On Chronic Systolic (Congestive) Heart Failure, Unspecified Diastolic (Congestive) Heart Failure, Acute Diastolic (Congestive) Heart Failure, Chronic Diastolic (Congestive) Heart Failure, Acute On Chronic Diastolic (Congestive) Heart Failure, Unspecified Combined Systolic (Congestive) And Diastolic (Congestive) Heart Failure, Acute Combined Systolic (Congestive) And Diastolic (Congestive) Heart Failure, Chronic Combined Systolic (Congestive) And Diastolic (Congestive) Heart Failure, Acute On Chronic Combined Systolic (Congestive) And Diastolic (Congestive) Heart Failure, Heart Failure, Unspecified, Postprocedural heart failure following cardiac surgery, Postprocedural heart failure following other surgery
Coronary Heart Disease	ICD9	411, 411.1, 411.81, 411.89, 413, 413.9, 414, 414.01, 414.02, 414.03, 414.04, 414.05, 414.06, 414.07, 414.1, 414.11, 414.19, 414.2, 414.3,	Postmyocardial infarction syndrome, Intermediate coronary syndrome, Acute coronary occlusion without myocardial infarction, Other acute and subacute forms of ischemic heart disease, other, Angina decubitus, Other and unspecified angina pectoris, Coronary atherosclerosis of unspecified type of vessel, native or graft, Coronary atherosclerosis of native coronary artery, Coronary atherosclerosis of autologous vein bypass graft, Coronary atherosclerosis of nonautologous biological bypass graft, Coronary atherosclerosis of artery bypass graft, Coronary atherosclerosis of unspecified bypass graft, Coronary atherosclerosis of

		414.4, 414.8, 414.9, 429.2, 996.03, V45.81, V45.82	native coronary artery of transplanted heart, Coronary atherosclerosis of bypass graft (artery) (vein) of transplanted heart, Aneurysm of heart (wall), Aneurysm of coronary vessels, Chronic ischemic heart disease, unspecified, Chronic total occlusion of coronary artery, Coronary atherosclerosis due to lipid rich plaque, Coronary atherosclerosis due to calcified coronary lesion, Other specified forms of chronic ischemic heart disease, Chronic ischemic heart disease, unspecified, Cardiovascular disease, unspecified, Mechanical complication due to coronary bypass graft, Aortocoronary bypass status, Percutaneous transluminal coronary angioplasty status
Coronary Heart Disease	ICD10	I20.0, I20.1, I20.8, I20.9, I21.01, I21.02, I21.09, I21.11, I21.19, I21.21, I21.29, I21.3, I21.4, I22.0, I22.1, I22.2, I22.8, I22.9, I23.0, I23.1, I23.2, I23.3, I23.4, I23.5, I23.6, I23.7, I23.8, I24.0, I24.8, I25.10, I25.110, I25.111, I25.118, I25.119, I25.3, I25.41, I25.42, I25.6, I25.700, I25.701, I25.708, I25.709, I25.710, I25.711, I25.718, I25.719, I25.720, I25.721, I25.728, I25.729, I25.730, I25.731, I25.738, I25.739, I25.790, I25.791, I25.798, I25.799, I25.810, I25.811, I25.812, I25.82, I25.83, I25.84, I25.89, I25.9, Z95.1, Z98.61	Unstable angina, Angina pectoris with documented spasm, Other forms of angina pectoris, Angina pectoris, unspecified, ST elevation (STEMI) myocardial infarction involving left main coronary artery, ST elevation (STEMI) myocardial infarction involving left anterior descending coronary artery, ST elevation (STEMI) myocardial infarction involving other coronary artery of anterior wall, ST elevation (STEMI) myocardial infarction involving right coronary artery, ST elevation (STEMI) myocardial infarction involving other coronary artery of inferior wall, ST elevation (STEMI) myocardial infarction involving left circumflex coronary artery, ST elevation (STEMI) myocardial infarction involving other sites, ST elevation (STEMI) myocardial infarction of unspecified site , Non-ST elevation (NSTEMI) myocardial infarction , Subsequent ST elevation (STEMI) myocardial infarction of anterior wall, Subsequent ST elevation (STEMI) myocardial infarction of inferior wall, Subsequent non-ST elevation (NSTEMI) myocardial infarction , Subsequent ST elevation (STEMI) myocardial infarction of other sites, Subsequent ST elevation (STEMI) myocardial infarction of unspecified site, Hemopericardium as current complication following acute myocardial infarction, Atrial septal defect as current complication following acute myocardial infarction, Ventricular septal defect as current complication following acute myocardial infarction, Rupture of cardiac wall without hemopericardium as current complication following acute myocardial infarction, Rupture of chordae tendineae as current complication following acute myocardial infarction, Rupture of papillary muscle as current complication following acute myocardial infarction, Thrombosis of atrium, auricular appendage, and ventricle as current complications following acute myocardial infarction, Postinfarction angina, Other current complications following acute myocardial infarction, Acute coronary thrombosis not resulting in myocardial infarction, Other forms of acute ischemic heart disease , Atherosclerotic heart disease of native coronary artery without angina pectoris, Atherosclerotic heart disease of native coronary artery with unstable angina pectoris, Atherosclerotic heart disease of native coronary artery with angina pectoris with documented spasm, Atherosclerotic heart disease of native coronary artery with other forms of angina pectoris, Atherosclerotic heart disease of native coronary artery with unspecified angina pectoris, Aneurysm of heart, Coronary artery aneurysm, Coronary artery dissection, Silent myocardial ischemia, Atherosclerosis of coronary artery bypass graft(s), unspecified, with unstable angina pectoris, Atherosclerosis of coronary artery bypass graft(s), unspecified, with angina pectoris with documented spasm, Atherosclerosis of coronary artery bypass graft(s), unspecified, with other forms of angina pectoris, Atherosclerosis of coronary artery bypass graft(s), unspecified, with unspecified angina pectoris, Atherosclerosis of autologous vein coronary artery bypass graft(s) with unstable angina pectoris, Atherosclerosis of autologous vein coronary artery bypass graft(s) with angina pectoris with documented spasm, Atherosclerosis of autologous vein coronary artery

			bypass graft(s) with other forms of angina pectoris, Atherosclerosis of autologous vein coronary artery bypass graft(s) with unspecified angina pectoris, Atherosclerosis of autologous artery coronary artery bypass graft(s) with unstable angina pectoris, Atherosclerosis of autologous artery coronary artery bypass graft(s) with angina pectoris with documented spasm, Atherosclerosis of autologous artery coronary artery bypass graft(s) with other forms of angina pectoris, Atherosclerosis of autologous artery coronary artery bypass graft(s) with unspecified angina pectoris, Atherosclerosis of nonautologous biological coronary artery bypass graft(s) with unstable angina pectoris, Atherosclerosis of nonautologous biological coronary artery bypass graft(s) with angina pectoris with documented spasm, Atherosclerosis of nonautologous biological coronary artery bypass graft(s) with other forms of angina pectoris, Atherosclerosis of nonautologous biological coronary artery bypass graft(s) with unspecified angina pectoris, Atherosclerosis of other coronary artery bypass graft(s) with unstable angina pectoris, Atherosclerosis of other coronary artery bypass graft(s) with angina pectoris with documented spasm, Atherosclerosis of other coronary artery bypass graft(s) with other forms of angina pectoris, Atherosclerosis of other coronary artery bypass graft(s) with unspecified angina pectoris, Atherosclerosis of coronary artery bypass graft(s) without angina pectoris , Atherosclerosis of native coronary artery of transplanted heart without angina pectoris, Atherosclerosis of bypass graft of coronary artery of transplanted heart without angina pectoris, Chronic total occlusion of coronary artery, Coronary atherosclerosis due to lipid rich plaque , Coronary atherosclerosis due to calcified coronary lesion, Other forms of chronic ischemic heart disease, Chronic ischemic heart disease, unspecified, Presence of aortocoronary bypass graft, Coronary angioplasty status
Hypertension	ICD9	401, 401.1, 401.9, 402, 402.01, 402.1, 402.11, 402.9, 402.91, 403, 403.01, 403.1, 403.11, 403.9, 403.91, 404, 404.01, 404.02, 404.03, 404.1, 404.11, 404.12, 404.13, 404.9, 404.91, 404.92, 404.93, 405.01, 405.09, 405.11, 405.19, 405.91, 405.99, 437.2, 796.2	Malignant essential hypertension, Benign essential hypertension, Unspecified essential hypertension, Malignant hypertensive heart disease without heart failure, Malignant hypertensive heart disease with heart failure, Benign hypertensive heart disease without heart failure, Benign hypertensive heart disease with heart failure, Unspecified hypertensive heart disease without heart failure, Unspecified hypertensive heart disease with heart failure, Hypertensive chronic kidney disease, malignant, with chronic kidney disease stage I through stage IV, or unspecified, Hypertensive chronic kidney disease, malignant, with chronic kidney disease stage V or end stage renal disease, Hypertensive chronic kidney disease, benign, with chronic kidney disease stage I through stage IV, or unspecified, Hypertensive chronic kidney disease, benign, with chronic kidney disease stage V or end stage renal disease, Hypertensive chronic kidney disease, unspecified, with chronic kidney disease stage I through stage IV, or unspecified, Hypertensive chronic kidney disease, unspecified, with chronic kidney disease stage V or end stage renal disease, Hypertensive heart and chronic kidney disease, malignant, without heart failure and with chronic kidney disease stage I through stage IV, or unspecified, Hypertensive heart and chronic kidney disease, malignant, with heart failure and with chronic kidney disease stage I through stage IV, or unspecified, Hypertensive heart and chronic kidney disease, malignant, without heart failure and with chronic kidney disease stage V or end stage renal disease, Hypertensive heart and chronic kidney disease, malignant, with heart failure and with chronic kidney disease stage V or end stage renal disease, Hypertensive heart and chronic kidney disease, benign, without heart failure and with chronic kidney disease stage I through stage IV, or unspecified, Hypertensive heart and chronic kidney disease, benign, with heart failure and with chronic

			kidney disease stage I through stage IV, or unspecified, Hypertensive heart and chronic kidney disease, benign, without heart failure and with chronic kidney disease stage V or end stage renal disease, Hypertensive heart and chronic kidney disease, benign, with heart failure and chronic kidney disease stage V or end stage renal disease, Hypertensive heart and chronic kidney disease, unspecified, without heart failure and with chronic kidney disease stage I through stage IV, or unspecified, Hypertensive heart and chronic kidney disease, unspecified, with heart failure and with chronic kidney disease stage I through stage IV, or unspecified, Hypertensive heart and chronic kidney disease, unspecified, without heart failure and with chronic kidney disease stage V or end stage renal disease, Hypertensive heart and chronic kidney disease, unspecified, with heart failure and chronic kidney disease stage V or end stage renal disease, Malignant renovascular hypertension, Other malignant secondary hypertension, Benign renovascular hypertension, Other benign secondary hypertension, Unspecified renovascular hypertension, Other unspecified secondary hypertension, Hypertensive encephalopathy, Elevated blood pressure reading without diagnosis of hypertension
Hypertension	ICD10	I10, I11.0, I11.9, I12.0, I12.9, I13.0, I13.10, I13.11, I13.2, I15.0, I15.1, I15.2, I15.8, I15.9	Essential (Primary) Hypertension, Hypertensive Heart Disease with Heart Failure, Hypertensive Heart Disease without Heart Failure, Hypertensive Chronic Kidney Disease with Stage 5 Chronic Kidney Disease or end stage renal disease, Hypertensive Chronic Kidney Disease with Stage 1 through Stage 4 Chronic Kidney Disease, or unspecified chronic kidney disease, Hypertensive heart and chronic kidney disease with heart failure and stage 1 through stage 4 chronic kidney disease, or unspecified chronic kidney disease , Hypertensive heart and chronic kidney disease without heart failure, with stage 1 through stage 4 chronic kidney disease, or unspecified chronic kidney disease , Hypertensive heart and chronic kidney disease without heart failure, with stage 5 chronic kidney disease, or end stage renal disease, Hypertensive heart and chronic kidney disease with heart failure and with stage 5 chronic kidney disease, or end stage renal disease, Renovascular Hypertension, Hypertension secondary to other renal disorders, Hypertension secondary to endocrine disorders, Other Secondary Hypertension, Secondary hypertension, unspecified
Valvular Disease	ICD9	35.05, 35.06, 35.1, 35.11, 35.12, 35.13, 35.14, 35.2, 35.21, 35.22, 35.23, 35.24, 35.25, 35.26, 35.27, 35.28, 35.96, 394, 394.1, 394.2, 394.9, 396, 396.1, 396.2, 396.3, 396.8, 396.9, V42.2, V43.3	Endovascular replacement of aortic valve, Transapical replacement of aortic valve, Open heart valvuloplasty without replacement, unspecified valve, Open heart valvuloplasty of aortic valve without replacement, Open heart valvuloplasty of mitral valve without replacement, Open heart valvuloplasty of pulmonary valve without replacement, Open heart valvuloplasty of tricuspid valve without replacement, Open and other replacement of unspecified heart valve, Open and other replacement of aortic valve with tissue graft, Open and other replacement of aortic valve, Open and other replacement of mitral valve with tissue graft, Open and other replacement of mitral valve, Open and other replacement of pulmonary valve with tissue graft, Open and other replacement of pulmonary valve, Open and other replacement of tricuspid valve with tissue graft, Open and other replacement of tricuspid valve, Percutaneous balloon valvuloplasty, Mitral stenosis, Rheumatic Mitral Insufficiency, Mitral stenosis with insufficiency, Other unspecified mitral valve disease, Mitral valve stenosis and aortic valve stenosis, Mitral valve stenosis and aortic valve insufficiency, Mitral valve insufficiency and aortic valve stenosis, Mitral valve insufficiency and aortic valve insufficiency, Multiple

			involvement of mitral and aortic valves, Mitral and aortic valve diseases, unspecified, Heart valve replaced by transplant, Heart valve replaced by other means
Valvular Disease	ICD10	I05.0, I05.1, I05.2, I05.8, I05.9, I06.8 , I06.9, I07.8, I07.9, I08.0, I08.1, I08.3, I08.8, I08.9, I09.1, I34.0, I34.1, I34.2, I34.8, I34.9, I35.0, I35.1, I35.2, I35.8, I35.9, I36.0, I36.1, I36.2, I36.8, I36.9, I37.0, I37.1, I37.2, I37.8, I37.9, I38	Rheumatic Mitral Stenosis, Rheumatic Mitral Insufficiency, Rheumatic Mitral Stenosis With Insufficiency, Other Rheumatic Mitral Valve Diseases, Rheumatic mitral valve disease, unspecified, Other rheumatic aortic valve diseases, Rheumatic aortic valve disease, unspecified, Other rheumatic tricuspid valve diseases, Rheumatic tricuspid valve disease, unspecified, Rheumatic Disorders Of Both Mitral And Aortic Valves, Rheumatic Disorders Of Both Mitral And tricuspid Valves, Combined rheumatic disorders of mitral, aortic, and tricuspid valves, Other Rheumatic Multiple Valve Diseases, Rheumatic Multiple Valve Disease, Unspecified, Rheumatic diseases of endocardium, valve unspecified, Nonrheumatic mitral (valve) insufficiency, Nonrheumatic mitral (valve) prolapse, Nonrheumatic mitral (valve) stenosis, Other nonrheumatic mitral valve disorders, Nonrheumatic mitral valve disorder, unspecified, Nonrheumatic aortic (valve) stenosis, Nonrheumatic aortic (valve) insufficiency, Nonrheumatic aortic (valve) stenosis with insufficiency, Other nonrheumatic aortic valve disorders, Nonrheumatic aortic valve disorder, unspecified, Nonrheumatic tricuspid (valve) stenosis, Nonrheumatic tricuspid (valve) insufficiency, Nonrheumatic tricuspid (valve) stenosis with insufficiency, Other nonrheumatic tricuspid valve disorders, Nonrheumatic tricuspid valve disorder, unspecified, Nonrheumatic pulmonary valve stenosis, Nonrheumatic pulmonary valve insufficiency, Nonrheumatic pulmonary valve stenosis with insufficiency, Other nonrheumatic pulmonary valve disorders, Nonrheumatic pulmonary valve disorder, unspecified, Endocarditis, valve unspecified
Valvular Disease	CPT	33400, 33401, 33403, 33405, 33406, 33411, 33412, 33413, 33420, 33422, 33425, 33426, 33427, 33430, 33606, 33611, 33612, 33645, 33665, 33670, 33681, 33684, 33688, 33690	Repair Of Aortic Valve, Valvuloplasty, Open, Valvuloplasty, W/Cp Bypass, Replacement Of Aortic Valve, Replacement Of Aortic Valve, Replacement Of Aortic Valve, Replacement Of Aortic Valve, Replacement Of Aortic Valve, Revision Of Mitral Valve, Revision Of Mitral Valve, Repair Of Mitral Valve, Repair Of Mitral Valve, Repair Of Mitral Valve, Replacement Of Mitral Valve, Anastomosis/artery-aorta, Repair double ventricle , Repair double ventricle , Revision of heart veins , Repair of heart defects, Repair of heart chambers , Repair heart septum defect , Repair heart septum defect , Repair heart septum defect , Reinforce pulmonary artery
Stroke/TIA	ICD9	362.31, 362.32, 362.33, 362.34, 388.02, 430, 431, 432.9, 433.01, 433.11, 433.21, 433.31, 433.81, 433.91, 434, 434.01, 434.1, 434.11, 434.9, 434.91, 435, 435.1, 435.2, 435.3, 435.8, 435.9, 437.1, 437.7, 437.9, 438.1,	Central retinal artery occlusion, Retinal arterial branch occlusion, Partial retinal arterial occlusion, Transient retinal arterial occlusion, Transient ischemic deafness, Subarachnoid hemorrhage, Intracerebral hemorrhage, Unspecified intracranial hemorrhage, Occlusion and stenosis of basilar artery with cerebral infarction, Occlusion and stenosis of carotid artery with cerebral infarction, Occlusion and stenosis of vertebral artery with cerebral infarction, Occlusion and stenosis of multiple and bilateral precerebral arteries with cerebral infarction, Occlusion and stenosis of other specified precerebral artery with cerebral infarction, Occlusion and stenosis of unspecified precerebral artery with cerebral infarction, Cerebral thrombosis without mention of cerebral infarction, Cerebral thrombosis with cerebral infarction, Cerebral embolism without mention of cerebral infarction, Cerebral embolism with cerebral infarction, Cerebral artery occlusion, unspecified without mention of cerebral infarction, Cerebral artery occlusion, unspecified with cerebral

		438.11, 438.12, 438.13, 438.14, 438.2, 438.21, 438.22, 438.81, 438.82, 438.83, 438.89, 438.9, 997.02, V12.54	infarction, Basilar artery syndrome, Vertebral artery syndrome , Subclavian steal syndrome, Vertebrobasilar artery syndrome, Other specified transient cerebral ischemias, Unspecified transient cerebral ischemia, Other generalized ischemic cerebrovascular disease, Transient global amnesia, Unspecified cerebrovascular disease, Late effects of cerebrovascular disease, speech and language deficit, unspecified, Late effects of cerebrovascular disease, aphasia, Late effects of cerebrovascular disease, dysphasia, Late effects of cerebrovascular disease, dysarthria, Late effects of cerebrovascular disease, fluency disorder, Late effects of cerebrovascular disease, hemiplegia affecting unspecified side, Late effects of cerebrovascular disease, hemiplegia affecting dominant side, Late effects of cerebrovascular disease, hemiplegia affecting nondominant side, Other late effects of cerebrovascular disease, apraxia, Other late effects of cerebrovascular disease, dysphagia, Other late effects of cerebrovascular disease, facial weakness, Other late effects of cerebrovascular disease, Unspecified late effects of cerebrovascular disease, Iatrogenic cerebrovascular infarction or hemorrhage, Personal history of transient ischemic attack (TIA), and cerebral infarction without residual deficits
Stroke/TIA	IDC10	G45.0, G45.1, G45.2, G45.3, G45.4, G45.8, G46.3, G46.4, H34.00, H34.01, H34.02, H34.03, H34.10, H34.11, H34.12, H34.13, H34.211, H34.212, H34.213, H34.219, H34.231, H34.232, H34.233, H34.239, H93.099, I60.9, I61.9, I62.9, I63.00, I63.011, I63.012, I63.019, I63.111, I63.112, I63.119, I63.12, I63.131, I63.132, I63.139, I63.19, I63.20, I63.211, I63.212, I63.219, I63.22, I63.231, I63.232, I63.239, I63.29, I63.30, I63.311, I63.312, I63.319, I63.321, I63.322, I63.329, I63.331, I63.332, I63.339, I63.341,	Vertebro-Basilar Artery Syndrome, Carotid Artery Syndrome, Multiple and bilateral precerebral artery syndromes, Amaurosis fugax, Transient Global Amnesia, Other transient cerebral ischemic attacks and related syndromes, Brain stem stroke syndrome, Cerebellar stroke syndrome, Transient Retinal Artery Occlusion, Unspecified Eye, Transient retinal artery occlusion, right eye, Transient retinal artery occlusion, left eye, Transient retinal artery occlusion, bilateral, Central retinal artery occlusion, unspecified eye, Central retinal artery occlusion, right eye, Central retinal artery occlusion, left eye, Central retinal artery occlusion, bilateral, Partial retinal artery occlusion, right eye, Partial retinal artery occlusion, left eye, Partial retinal artery occlusion, bilateral, Partial Retinal Artery Occlusion, Unspecified Eye, Retinal artery branch occlusion, right eye, Retinal artery branch occlusion, left eye, Retinal artery branch occlusion, bilateral, Retinal Artery Branch Occlusion, Unspecified Eye, Unspecified Degenerative and Vascular Disorders of Unspecified Ear, Nontraumatic Subarachnoid Hemorrhage, Unspecified, Nontraumatic intracerebral Hemorrhage, unspecified, Nontraumatic Intracranial Hemorrhage, Unspecified, Cerebral infarction due to thrombosis of unspecified precerebral artery, Cerebral infarction due to thrombosis of right vertebral artery, Cerebral infarction due to thrombosis of left vertebral artery, Cerebral infarction due to thrombosis of unspecified vertebral artery, Cerebral infarction due to embolism of right vertebral artery, Cerebral infarction due to embolism of left vertebral artery, Cerebral infarction due to embolism of unspecified vertebral artery , Cerebral infarction due to embolism of basilar artery, Cerebral infarction due to embolism of right carotid artery, Cerebral infarction due to embolism of left carotid artery, Cerebral infarction due to embolism of unspecified carotid artery, Cerebral infarction due to embolism of other precerebral artery, Cerebral infarction due to unspecified occlusion or stenosis of unspecified precerebral arteries , Cerebral infarction due to unspecified occlusion or stenosis of right vertebral arteries, Cerebral infarction due to unspecified occlusion or stenosis of left vertebral arteries, Cerebral infarction due to unspecified occlusion or stenosis of unspecified vertebral arteries, Cerebral Infraction Due to Unspecified Occlusion or Stenosis of Basilar Arteries, Cerebral infarction due to unspecified occlusion or stenosis of right carotid arteries, Cerebral infarction due to unspecified occlusion or stenosis of left carotid arteries, Cerebral infarction due to

		I63.342, I63.349, I63.40, I63.411, I63.412, I63.419, I63.421, I63.422, I63.429, I63.431, I63.432, I63.439, I63.49, I63.50, I63.511, I63.512, I63.519, I63.521, I63.522, I63.529, I63.531, I63.532, I63.539, I63.541, I63.542, I63.549, I63.59, I63.6, I63.8, I63.9, I66.01, I66.02, I66.03, I66.09, I66.11, I66.12, I66.13, I66.19, I66.21, I66.22, I66.23, I66.29, I66.3, I66.8, I66.9, I67.81, I67.82, I67.841, I67.848, I67.89, I67.9, I69.80, I69.81, I69.820, I69.821, I69.822, I69.823, I69.828, I69.831, I69.832, I69.833, I69.834, I69.839, I69.841, I69.842, I69.843, I69.844, I69.849, I69.851, I69.852, I69.853, I69.854, I69.859, I69.861, I69.862, I69.863, I69.864, I69.865, I69.869, I69.890, I69.891, I69.892, I69.893, I69.898, I69.90, I69.91, I69.920, I69.921, I69.922, I69.923, I69.928, I69.931,	unspecified occlusion or stenosis of unspecified carotid arteries, Cerebral infarction due to unspecified occlusion or stenosis of other precerebral arteries, Cerebral infarction due to thrombosis of unspecified cerebral artery , Cerebral infarction due to thrombosis of right middle cerebral artery, Cerebral infarction due to thrombosis of left middle cerebral artery, Cerebral infarction due to thrombosis of unspecified middle cerebral artery, Cerebral infarction due to thrombosis of right anterior cerebral artery, Cerebral infarction due to thrombosis of left anterior cerebral artery, Cerebral infarction due to thrombosis of unspecified anterior cerebral artery, Cerebral infarction due to thrombosis of right posterior cerebral artery, Cerebral infarction due to thrombosis of left posterior cerebral artery, Cerebral infarction due to thrombosis of unspecified posterior cerebral artery, Cerebral infarction due to thrombosis of right cerebellar artery, Cerebral infarction due to thrombosis of left cerebellar artery, Cerebral infarction due to thrombosis of unspecified cerebellar artery, Cerebral infarction due to embolism of unspecified cerebral artery , Cerebral infarction due to embolism of right middle cerebral artery, Cerebral infarction due to embolism of left middle cerebral artery, Cerebral infarction due to embolism of unspecified middle cerebral artery, Cerebral infarction due to embolism of right anterior cerebral artery, Cerebral infarction due to embolism of left anterior cerebral artery, Cerebral infarction due to embolism of unspecified anterior cerebral artery, Cerebral infarction due to embolism of right posterior cerebral artery, Cerebral infarction due to embolism of left posterior cerebral artery, Cerebral infarction due to embolism of unspecified posterior cerebral artery , Cerebral infarction due to embolism of other cerebral artery, Cerebral infarction due to unspecified occlusion or stenosis of unspecified cerebral artery , Cerebral infarction due to unspecified occlusion or stenosis of right middle cerebral artery, Cerebral infarction due to unspecified occlusion or stenosis of left middle cerebral artery, Cerebral infarction due to unspecified occlusion or stenosis of unspecified middle cerebral artery, Cerebral infarction due to unspecified occlusion or stenosis of right anterior cerebral artery, Cerebral infarction due to unspecified occlusion or stenosis of left anterior cerebral artery, Cerebral infarction due to unspecified occlusion or stenosis of unspecified anterior cerebral artery, Cerebral infarction due to unspecified occlusion or stenosis of right posterior cerebral artery, Cerebral infarction due to unspecified occlusion or stenosis of left posterior cerebral artery, Cerebral infarction due to unspecified occlusion or stenosis of unspecified posterior cerebral artery, Cerebral infarction due to unspecified occlusion or stenosis of right cerebellar artery, Cerebral infarction due to unspecified occlusion or stenosis of left cerebellar artery, Cerebral infarction due to unspecified occlusion or stenosis of unspecified cerebellar artery, Cerebral infarction due to unspecified occlusion or stenosis of other cerebral artery , Cerebral infarction due to cerebral venous thrombosis, nonpyogenic, Other cerebral infarction, Cerebral infarction, unspecified, Occlusion and stenosis of right middle cerebral artery, Occlusion and stenosis of left middle cerebral artery, Occlusion and stenosis of bilateral middle cerebral arteries, Occlusion and stenosis of unspecified middle cerebral artery, Occlusion and stenosis of right anterior cerebral artery, Occlusion and stenosis of left anterior cerebral artery, Occlusion and stenosis of bilateral anterior cerebral arteries, Occlusion and stenosis of unspecified anterior cerebral artery, Occlusion and stenosis of right posterior cerebral artery, Occlusion and stenosis of left posterior cerebral artery, Occlusion and stenosis of bilateral posterior cerebral arteries, Occlusion and stenosis of unspecified posterior cerebral artery , Occlusion and stenosis of cerebellar arteries, Occlusion and stenosis of other cerebral arteries, Occlusion and stenosis of unspecified cerebral artery, Acute

		I69.932, I69.933, I69.934, I69.939, I69.941, I69.942, I69.943, I69.944, I69.949, I69.951, I69.952, I69.953, I69.954, I69.959, I69.961, I69.962, I69.963, I69.964, I69.965, I69.969, I69.990, I69.991, I69.992, I69.993, I69.998, I97.810, I97.811, I97.820, I97.821, Z86.73	cerebrovascular insufficiency, Cerebral ischemia, Reversible cerebrovascular vasoconstriction syndrome, Other cerebrovascular vasospasm and vasoconstriction , Other cerebrovascular disease, Cerebrovascular Disease, unspecified, Unspecified sequelae of other cerebrovascular disease, Cognitive deficits following other cerebrovascular disease, Aphasia following other cerebrovascular disease, Dysphasia following other cerebrovascular disease, Dysarthria following other cerebrovascular disease, Fluency disorder following other cerebrovascular disease, Other speech and language deficits following other cerebrovascular disease, Monoplegia of upper limb following other cerebrovascular disease affecting right dominant side, Monoplegia of upper limb following other cerebrovascular disease affecting left dominant side, Monoplegia of upper limb following other cerebrovascular disease affecting right non-dominant side, Monoplegia of upper limb following other cerebrovascular disease affecting left non-dominant side, Monoplegia of upper limb following other cerebrovascular disease affecting unspecified side, Monoplegia of lower limb following other cerebrovascular disease affecting right dominant side, Monoplegia of lower limb following other cerebrovascular disease affecting left dominant side, Monoplegia of lower limb following other cerebrovascular disease affecting right non-dominant side, Monoplegia of lower limb following other cerebrovascular disease affecting left non-dominant side, Monoplegia of lower limb following other cerebrovascular disease affecting unspecified side, Hemiplegia and hemiparesis following other cerebrovascular disease affecting right dominant side, Hemiplegia and hemiparesis following other cerebrovascular disease affecting left dominant side, Hemiplegia and hemiparesis following other cerebrovascular disease affecting right non-dominant side, Hemiplegia and hemiparesis following other cerebrovascular disease affecting left non-dominant side, Hemiplegia and hemiparesis following other cerebrovascular disease affecting unspecified side, Other paralytic syndrome following other cerebrovascular disease affecting right dominant side, Other paralytic syndrome following other cerebrovascular disease affecting left dominant side, Other paralytic syndrome following other cerebrovascular disease affecting right non-dominant side, Other paralytic syndrome following other cerebrovascular disease affecting left non-dominant side, Other paralytic syndrome following other cerebrovascular disease, bilateral, Other paralytic syndrome following other cerebrovascular disease affecting unspecified side, Apraxia following other cerebrovascular disease, Dysphagia following other cerebrovascular disease, Facial weakness following other cerebrovascular disease, Ataxia following other cerebrovascular disease, Other sequelae of other cerebrovascular disease , Unspecified Sequelae of unspecified cerebrovascular disease, Cognitive deficits following unspecified cerebrovascular disease, Aphasia following unspecified cerebrovascular disease, Dysphasia following unspecified cerebrovascular disease , Dysarthria following unspecified Cerebrovascular disease, Fluency disorder following unspecified cerebrovascular disease , Other speech and language deficits following unspecified cerebrovascular disease, Monoplegia of upper limb following unspecified cerebrovascular disease affecting right dominant side, Monoplegia of upper limb following unspecified cerebrovascular disease affecting left dominant side, Monoplegia of upper limb following unspecified cerebrovascular disease affecting right non-dominant side, Monoplegia of upper limb following unspecified cerebrovascular disease affecting left non-dominant side, Monoplegia of upper limb following unspecified cerebrovascular disease affecting unspecified side, Monoplegia of lower limb following unspecified cerebrovascular disease affecting right dominant side,

			Monoplegia of lower limb following unspecified cerebrovascular disease affecting left dominant side, Monoplegia of lower limb following unspecified cerebrovascular disease affecting right non-dominant side, Monoplegia of lower limb following unspecified cerebrovascular disease affecting left non-dominant side, Monoplegia of lower limb following unspecified cerebrovascular disease affecting unspecified side, Hemiplegia and hemiparesis following unspecified cerebrovascular disease affecting right dominant side , Hemiplegia and hemiparesis following unspecified cerebrovascular disease affecting left dominant side , Hemiplegia and hemiparesis following unspecified cerebrovascular disease affecting right non-dominant side, Hemiplegia and hemiparesis following unspecified cerebrovascular disease affecting left non-dominant side , Hemiplegia and hemiparesis following unspecified cerebrovascular disease affecting unspecified side , Other paralytic syndrome following unspecified cerebrovascular disease affecting right dominant side, Other paralytic syndrome following unspecified cerebrovascular disease affecting left dominant side, Other paralytic syndrome following unspecified cerebrovascular disease affecting right non-dominant side, Other paralytic syndrome following unspecified cerebrovascular disease affecting left non-dominant side, Other paralytic syndrome following unspecified cerebrovascular disease, bilateral, Other paralytic syndrome following unspecified cerebrovascular disease affecting unspecified side, Apraxia following unspecified cerebrovascular disease, Dysphagia following unspecified cerebrovascular disease , Facial weakness following unspecified cerebrovascular disease , Ataxia following unspecified cerebrovascular disease, Other sequelae following unspecified cerebrovascular disease , Intraoperative Cerebrovascular Infarction During cardiac surgery, Intraoperative cerebrovascular infarction during other surgery , Postprocedural cerebrovascular infarction during cardiac surgery, Postprocedural cerebrovascular infarction during other surgery, Personal history of transient ischemic attack (TIA), and cerebral infarction without residual deficits
Myocardial Infarction	ICD9	410, 410.01, 410.02, 410.1, 410.11, 410.12, 410.2, 410.21, 410.22, 410.3, 410.31, 410.32, 410.4, 410.41, 410.42, 410.5, 410.51, 410.52, 410.6, 410.61, 410.62, 410.7, 410.71, 410.72, 410.8, 410.81, 410.82, 410.9, 410.91, 410.92, 412, 429.79	Acute myocardial infarction of anterolateral wall, episode of care unspecified, Acute myocardial infarction of anterolateral wall, initial episode of care, Acute myocardial infarction of anterolateral wall, subsequent episode of care , Acute myocardial infarction of other anterior wall, episode of care unspecified, Acute myocardial infarction of other anterior wall, initial episode of care, Acute myocardial infarction of other anterior wall, subsequent episode of care, Acute myocardial infarction of inferolateral wall, episode of care unspecified, Acute myocardial infarction of inferolateral wall, initial episode of care, Acute myocardial infarction of inferolateral wall, subsequent episode of care, Acute myocardial infarction of inferoposterior wall, episode of care unspecified, Acute myocardial infarction of inferoposterior wall, initial episode of care, Acute myocardial infarction of inferoposterior wall, subsequent episode of care, Acute myocardial infarction of other inferior wall, episode of care unspecified, Acute myocardial infarction of other inferior wall, initial episode of care, Acute myocardial infarction of other inferior wall, subsequent episode of care, Acute myocardial infarction of other lateral wall, episode of care unspecified, Acute myocardial infarction of other lateral wall, initial episode of care, Acute myocardial infarction of other lateral wall, subsequent episode of care, True posterior wall infarction, episode of care unspecified, True posterior wall infarction, initial episode of care, True posterior wall infarction, subsequent episode of care, Subendocardial infarction, episode of care unspecified, Subendocardial infarction, initial episode of care, Subendocardial infarction, subsequent episode of care, Acute myocardial infarction of other specified sites, episode of care unspecified, Acute

			myocardial infarction of other specified sites, initial episode of care, Acute myocardial infarction of other specified sites, subsequent episode of care, Acute myocardial infarction of unspecified site, episode of care unspecified, Acute myocardial infarction of unspecified site, initial episode of care, Acute myocardial infarction of unspecified site, subsequent episode of care, Old myocardial infarction , Certain sequelae of myocardial infarction, not elsewhere classified, other
Myocardial Infarction	ICD10	I21.01, I21.02, I21.09, I21.11, I21.19, I21.21, I21.29, I21.3, I21.4, I22.0, I22.1, I22.2, I22.8, I22.9, I23.0, I23.1, I23.2, I23.3, I23.4, I23.5, I23.6, I23.7, I23.8, I24.1, I25.2	ST elevation (STEMI) myocardial infarction involving left main coronary artery, ST elevation (STEMI) myocardial infarction involving left anterior descending coronary artery, ST elevation (STEMI) myocardial infarction involving other coronary artery of anterior wall, ST elevation (STEMI) myocardial infarction involving right coronary artery, ST elevation (STEMI) myocardial infarction involving other coronary artery of inferior wall, ST elevation (STEMI) myocardial infarction involving left circumflex coronary artery, ST elevation (STEMI) myocardial infarction involving other sites, ST elevation (STEMI) myocardial infarction of unspecified site , Non-ST elevation (NSTEMI) myocardial infarction , Subsequent ST elevation (STEMI) myocardial infarction of anterior wall, Subsequent ST elevation (STEMI) myocardial infarction of inferior wall, Subsequent non-ST elevation (NSTEMI) myocardial infarction , Subsequent ST elevation (STEMI) myocardial infarction of other sites, Subsequent ST elevation (STEMI) myocardial infarction of unspecified site, Hemopericardium as current complication following acute myocardial infarction, Atrial septal defect as current complication following acute myocardial infarction, Ventricular septal defect as current complication following acute myocardial infarction, Rupture of cardiac wall without hemopericardium as current complication following acute myocardial infarction, Rupture of chordae tendineae as current complication following acute myocardial infarction, Rupture of papillary muscle as current complication following acute myocardial infarction, Thrombosis of atrium, auricular appendage, and ventricle as current complications following acute myocardial infarction, Postinfarction angina, Other current complications following acute myocardial infarction, Dressler's syndrome, Old myocardial infarction
Atrial fibrillation (AF) defined as ECG diagnosis of AF, ≥1 inpatient diagnosis code, ≥1 procedural code, or ≥2 codes of any type[6]<br>Myocardial infarction defined as ≥2 codes from any setting[7]<br>Ischemic stroke defined as ≥2 codes from any setting[8]<br>Heart failure defined as ≥1 inpatient diagnosis code			

Supplementary Table 11. Yield of vital sign extractions using Bio+DischargeSummaryBERT trained for two versus five epochs

Training epochs[1]	Vital sign[2]		
	Blood pressure	Weight	Height
Two	2,996	5,508	2,713
Five	8,976	5,563	2,667

[1]Table reflects results of Bio+DischargeSummaryBERT models evaluated on a holdout set of 24,229 reports from 1,000 randomly sampled individuals occurring during 3 years prior to start of follow-up and not included in model training
[2]Reflect vital sign yield after standardized post-processing (see main text)

Supplementary Table 12. Ablation study for post-processing steps performed on NLP vital identifications

Post-processing step	Positive predictive value		
	Blood pressure	Weight	Height
Step 1: Extended model identifications	100%	78%	60%
Step 2 & 3: Harmonizing units and imposing physiological constraints	100%	91%	100%
Step 4: Filtering out "ideal body weight"	100%	100%	100%
Calculated using chart review by a study cardiologist (SK) on 50 randomly selected samples			

Supplementary Figure 1. Distribution of office visits and primary care office visits per person in C3PO versus Convenience Samples

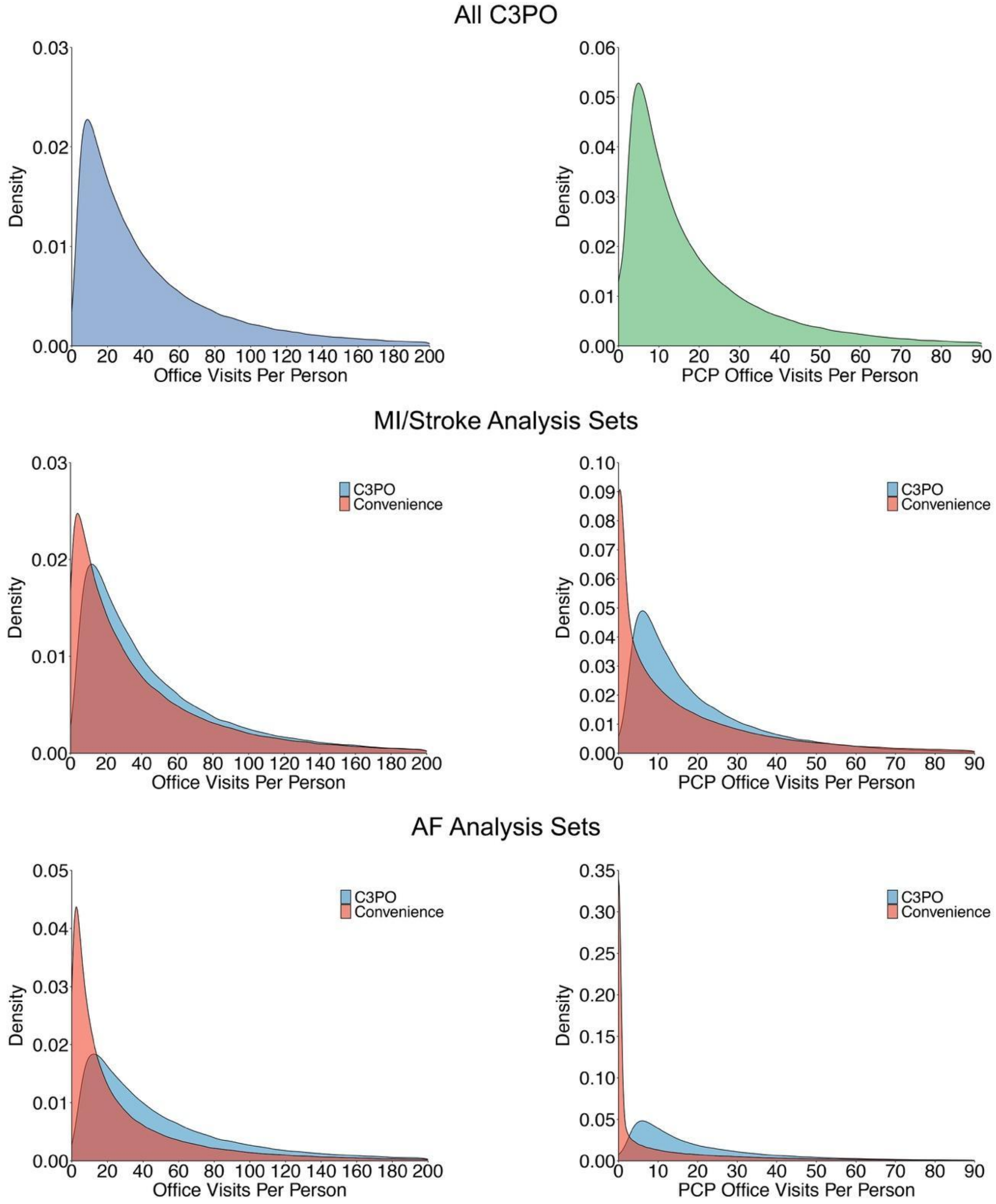

Depicted is the distribution of office visits (left panels) and primary care office visits (right panels) in all of C3PO (top panels), the MI/stroke analysis sets (middle panels), and the AF analysis sets (bottom panels). The MI/stroke and AF analysis sets depict distributions both for C3PO (blue) and the respective Convenience Samples (red).

Supplementary Figure 2. Example clinical note with NLP extracted vital signs

File type: Outpatient History and Physical
Height Identified: 61|.|5|inches
Weight Identified: 121|pounds
Systolic Identified: 125.0
Diastolic Identified: 66.0

****
SUBJECT: Pulmonary consult.

This was a first visit with me for this [removed_age] -year-old woman, who was referred for evaluation of lung nodules, multiple lung cysts, recent spontaneous pneumothorax. She had a spontaneous pneumothorax this past February.

PAST MEDICAL HISTORY: Head trauma, breast lesion, palpitations, pulmonary nodules, benign positional vertigo, depression, cervical dysplasia, HPV and genital herpes. Low back pain. Thyroid, thyroidectomy for papillary thyroid cancer.

REVIEW OF SYSTEMS: Negative for fevers, chills, sweats, weight gain or weight loss.

MEDICATIONS: Synthroid 125 mcg daily, and metoprolol 12.5 mg b.i.d.

FAMILY HISTORY: Her mother has diabetes, mini strokes, COPD, and asthma but was a nonsmoker. Her father died when he was 45 from leukemia. She has a half sister (same mother) who has had two blood clots. Her half brother has hypertension. She has no children.

SOCIAL HISTORY: She attended high school, but not college. She has worked in retail and in gymnasia.

On physical exam she was well-appearing and walked without difficulty from the waiting area to the office. **Her blood pressure was 125/66** in the right arm (she reports usually runs [removed_phi_number]). Temperature 98.2, pulse, respiratory rate 16 unlabored, oxygen saturation 96% on room air, **height 61.5 inches**, **weight 121 pounds**, BMI 22.5, pain level zero. Her chest was resonant to percussion and clear on auscultation. Heart had a regular rate and rhythm without click, rub, gallop, or murmur. Her extremities had no clubbing, cyanosis or edema.

Lung imaging studies-- She had a chest CT on [removed_date] for evaluation of pulmonary nodule that was seen on a coronary CTA. This chest CT showed an irregular 9 mm nodule in the right lower lobe, 4 mm nodule in the left upper lobe, 4 mm subpleural nodule in the right middle lobe, 4 mm nodule in the right middle lobe. Several thin-walled cyst in the lung.

In summary, this is a [removed_age]-year-old woman who had a spontaneous pneumothorax this past February. It spontaneously resolved without intervention. She also has had some known small bilateral pulmonary nodules and a few bilateral thin walled pulmonary cysts (10 to 15). The differential diagnosis for the combination of nodules and thin-walled cysts in spontaneous pneumothorax includes emphysema, lymphangioleiomyomatosis, tuberous sclerosis, hypersensitivity pneumonitis, and Birt-Hogg-Dube syndrome (FLCN gene mutation). I will add on testing for the FLCN gene. We will also schedule her for a repeat CT this July.

Depicted is an example outpatient note with NLP-extracted vital signs depicted at the top, and shown in bold within the note text. The note has been edited for brevity and to remove potentially identifying information.

Supplementary Figure 3. Agreement between tabular and natural language processing-extracted vital signs by year of extraction.

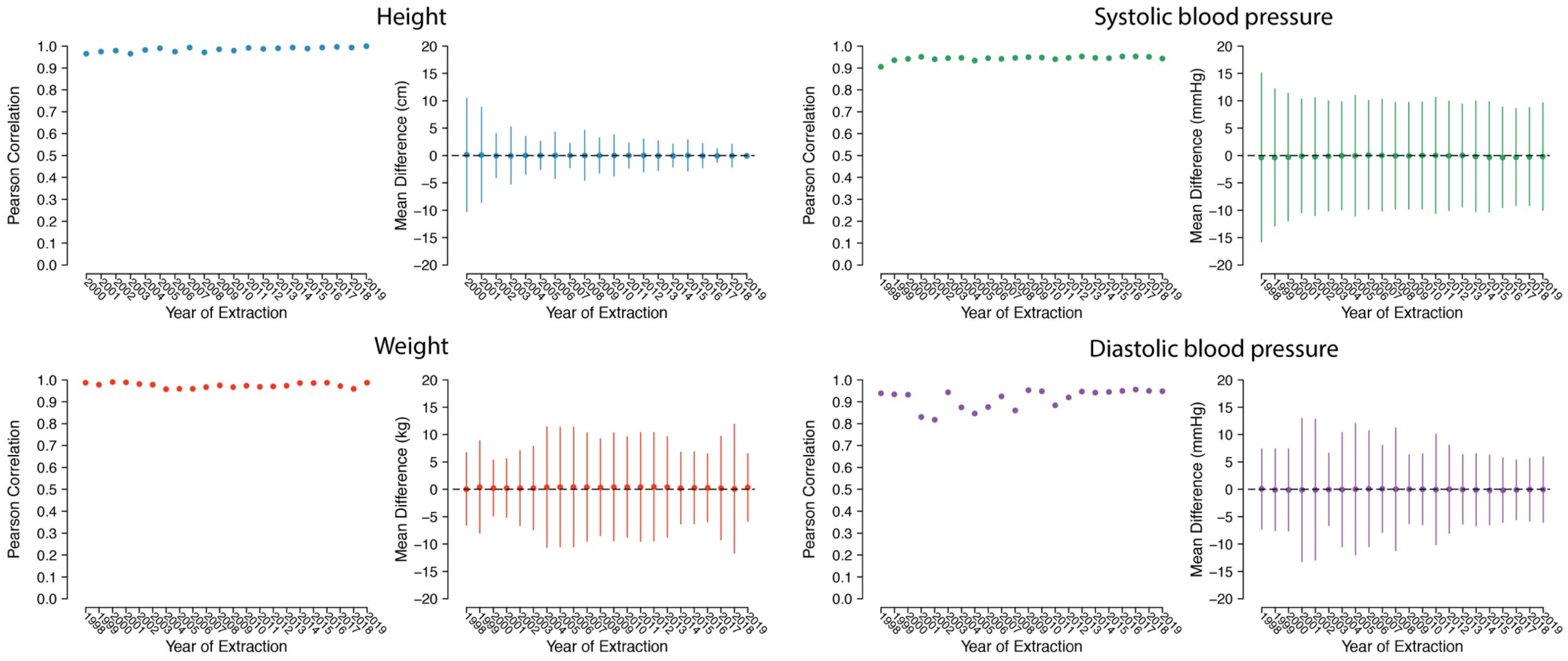

Depicted is agreement between vital signs obtained from tabular data and those obtained from our NLP model among individuals with values obtained on the same day, stratified by year of extraction (x-axis). Within each vital sign, left panels depict the Pearson correlation between tabular and NLP values, and right panels depict the difference between the paired values, where positive values denote tabular values greater than corresponding NLP values and negative values denote tabular values lower than corresponding NLP values. In the right plots, error bars depict 1.96 standard deviations above and below the observed mean difference.

Supplementary Figure 4. Flow diagrams for C3PO PCE and CHARGE-AF samples

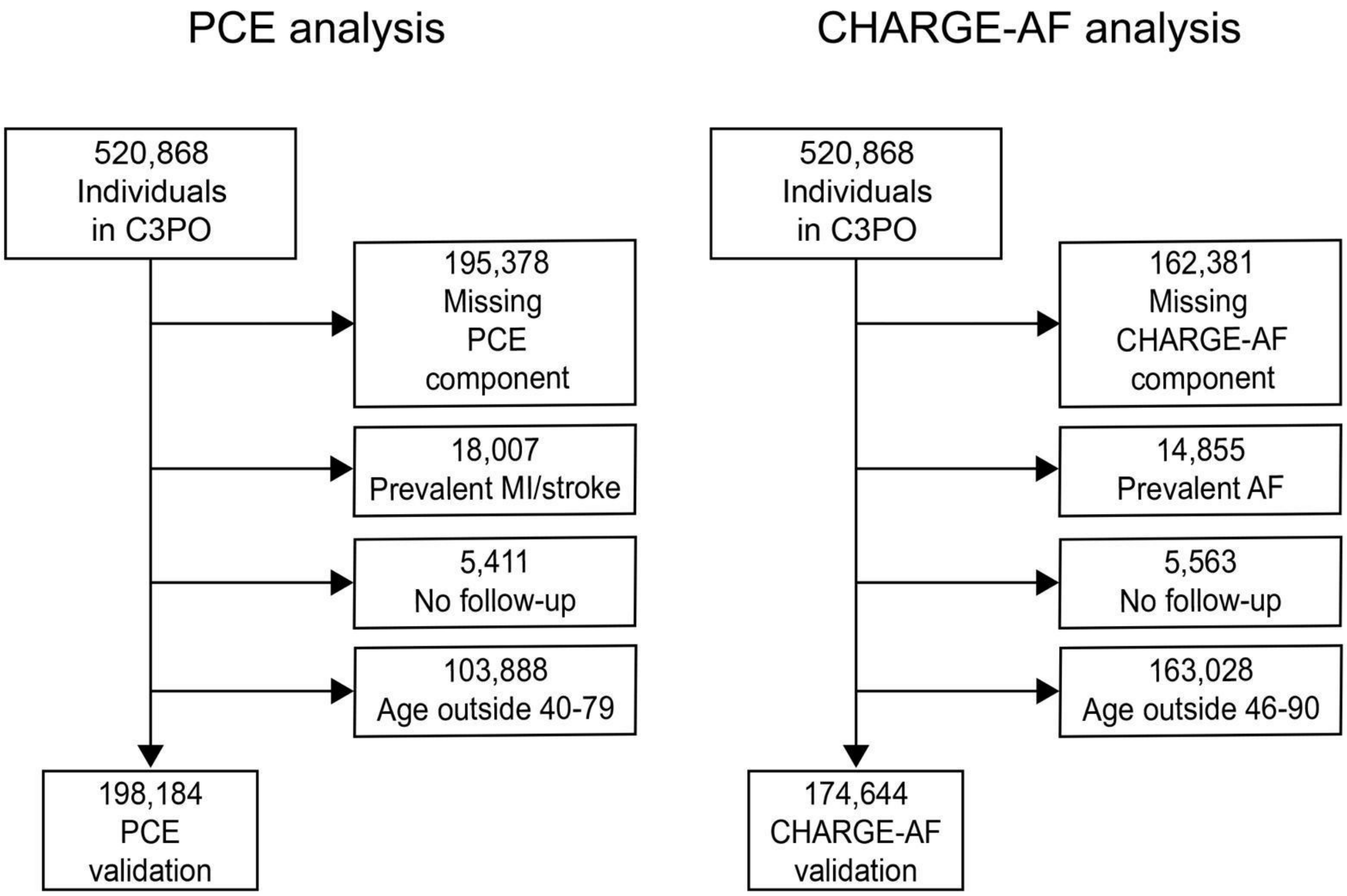

Depicted is the construction of the C3PO-based CHARGE-AF and PCE analysis samples, including relevant exclusions.

Supplementary Figure 5. Predicted event risk in C3PO CHARGE-AF and PCE analysis sets

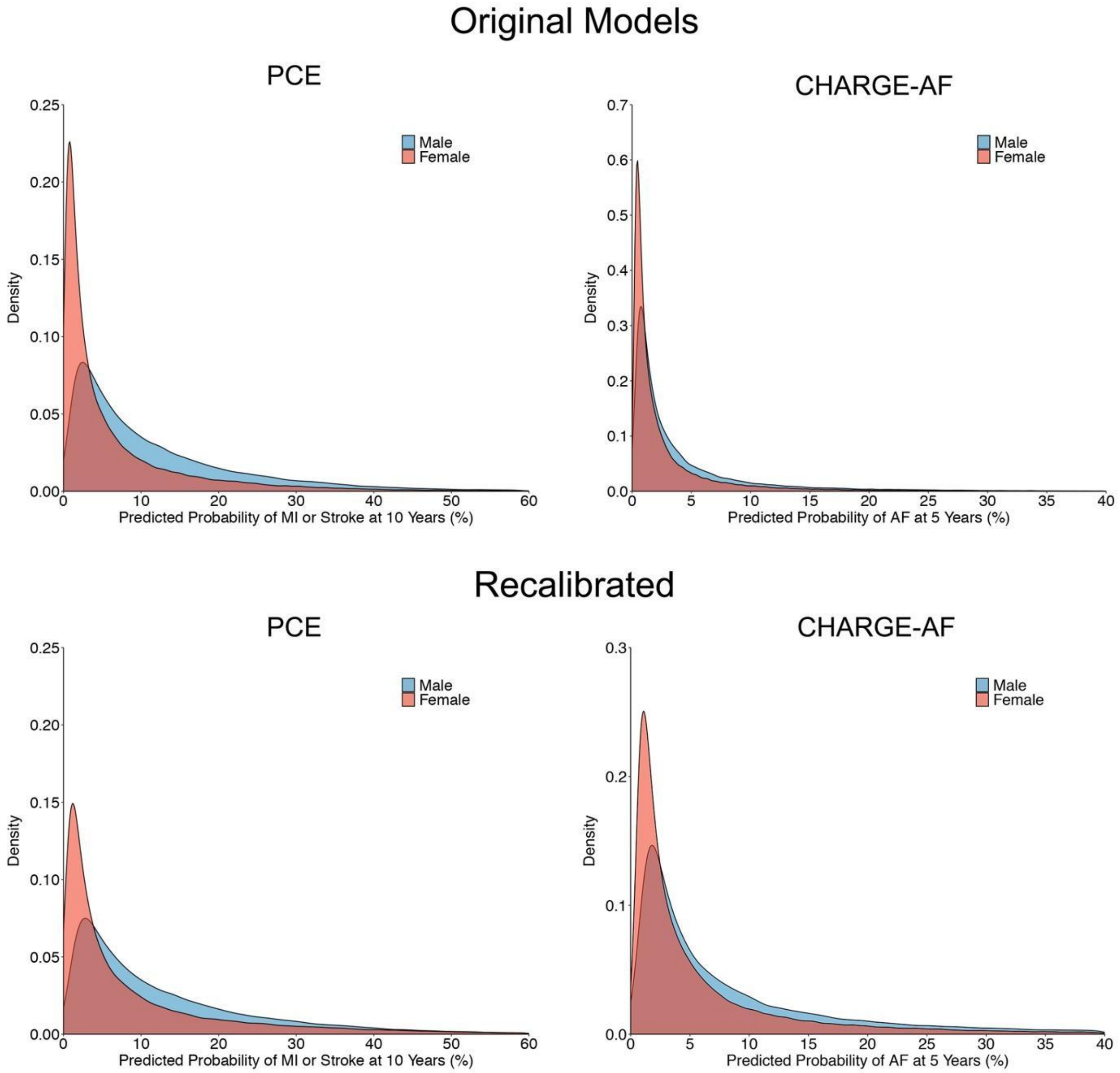

Depicted is sex-stratified the distribution of predicted 10-year MI/stroke risk according to PCE models (left panels) and the 5-year predicted AF risk according to the CHARGE-AF score (right panels). Females are depicted in red and males in blue. Top panels reflect predicted risks obtained using the original published equations. Bottom panels reflect predicted risks after recalibration to the C3PO baseline hazard.

Supplementary Figure 6. Cumulative risk of events stratified by predicted risk

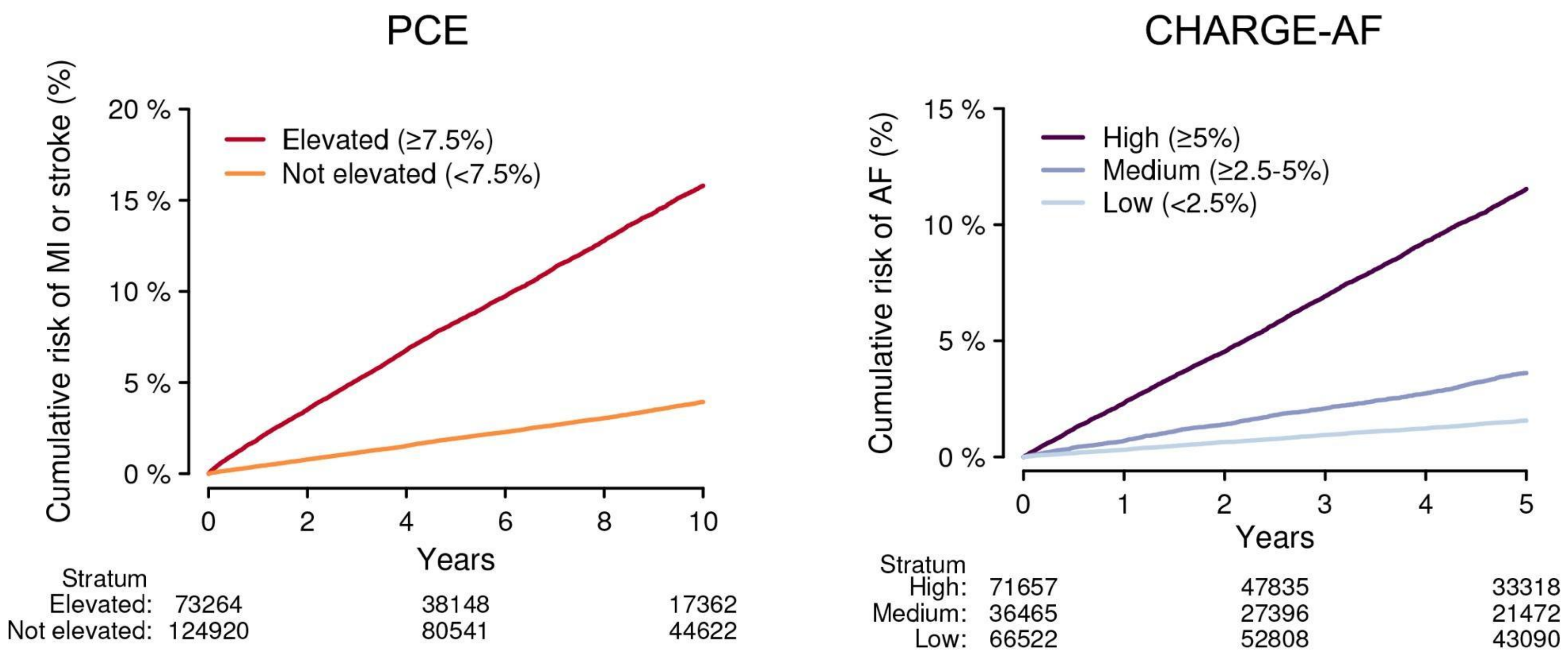

Depicted are the cumulative risks MI/stroke (left) and AF (right), stratified by level of predicted risk according to the Pooled Cohort Equations (PCE, left) and the CHARGE-AF score (right). Given substantial miscalibration using the original CHARGE-AF score, the model recalibrated to the baseline AF hazard of the sample was used for stratification. The risk levels depicted are those defining elevated atherosclerotic disease risk in the 2019 American Heart Association/American College of Cardiology primary prevention guidelines[9] (Elevated: ≥7.5%, Not elevated: <7.5%), and those previously proposed in the original CHARGE-AF derivation study[10] (High: ≥7.5%, Medium: ≥2.5-5%, Low <2.5%), respectively. The number of individuals remaining at risk is depicted below each plot. The x-axis depicts the number of years since start of follow-up, corresponding to the relevant prediction window for each score.

Supplementary Figure 7. Calibration of original models in C3PO and Convenience Samples

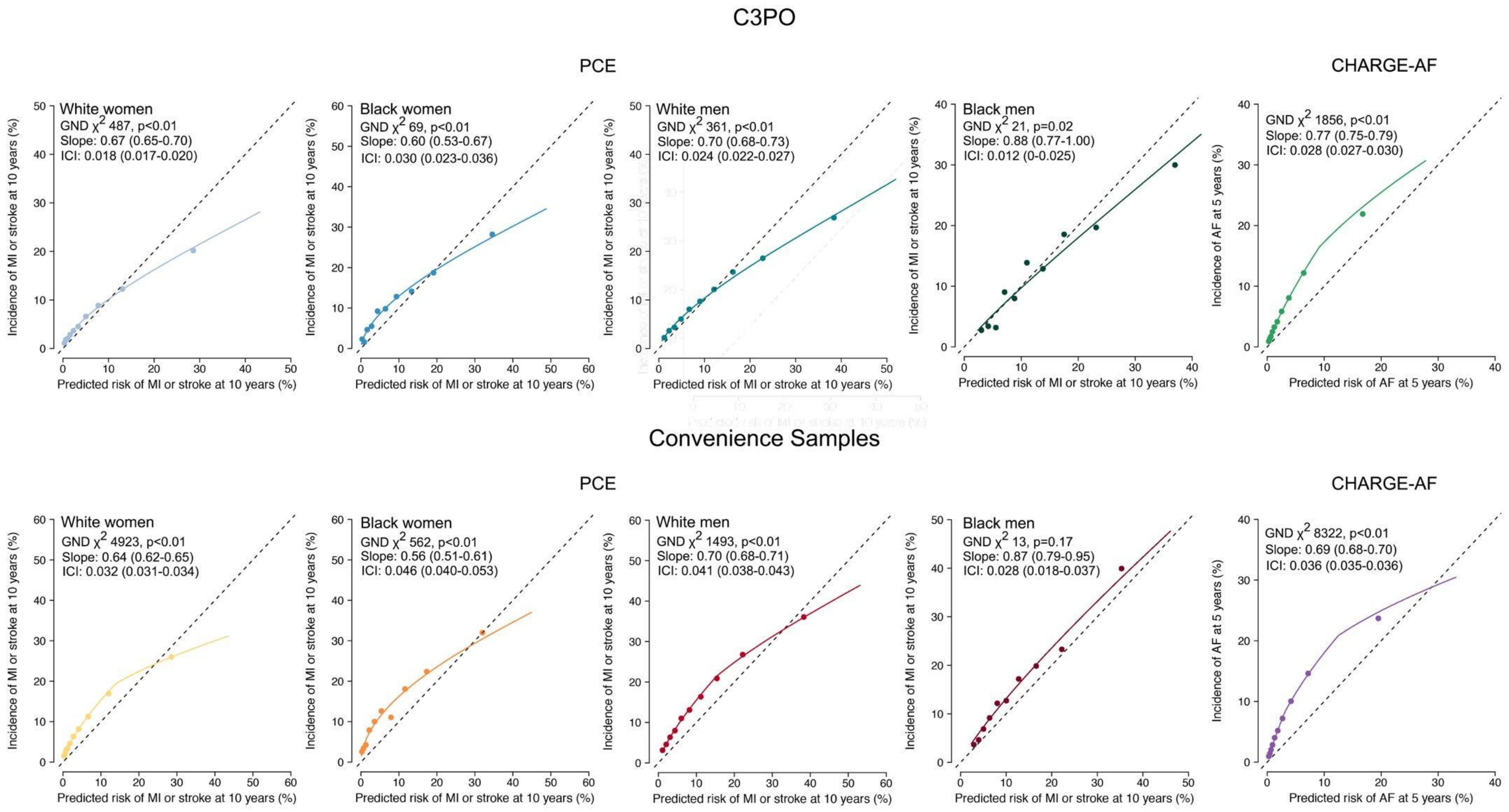

Depicted are calibration plots demonstrating agreement between predicted event risk (x-axis) and observed cumulative event incidence (y-axis) for the Pooled Cohort Equations (PCE, left four panels) and the CHARGE-AF score (right panels) in C3PO (upper panels) versus the Convenience Samples (bottom panels). In each plot, each point represents a decile of predicted risk. Events correspond to the prediction target of each score (i.e., 10-year MI or stroke for the PCE and 5-year incident AF for CHARGE-AF). Perfect calibration is indicated by the hashed diagonal line, denoting perfect correspondence between predicted and observed risk. The Greenwood-Nam-D'Agostino test of calibration is shown for each plot (where a lower chi-squared value indicates better fit), the calibration slope (where a calibration slope of one is optimal), and integrated calibration index (ICI, a measure of prediction error in which lower values indicate more accurate predictions) is shown for each model. Since the PCE score comprises four separate models stratified on the basis of sex and race, the curve for each score is represented separately (see legend). Each plot also depicts a fitted calibration curve obtained using adaptive hazard regression[3] relating predicted risk and observed event risk.

Supplementary Figure 8. Calibration of recalibrated models in C3PO and Convenience Samples

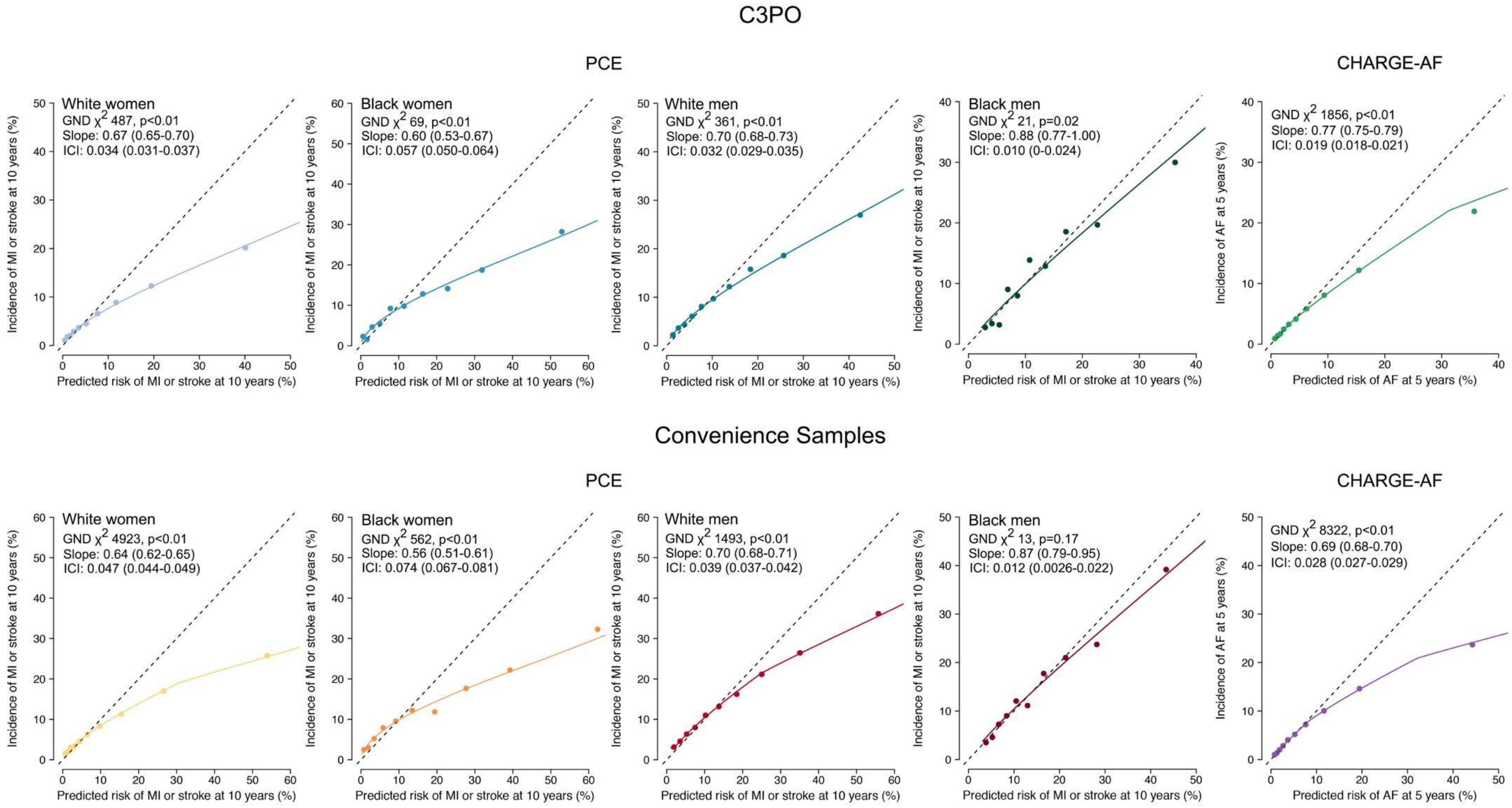

Depicted are calibration plots demonstrating agreement between predicted event risk (x-axis) and observed cumulative event incidence (y-axis) for the Pooled Cohort Equations (PCE, left four panels) and the CHARGE-AF score (right panels) in C3PO (upper panels) versus the Convenience Samples (bottom panels). In each plot, each point represents a decile of predicted risk. Events correspond to the prediction target of each score (i.e., 10-year MI or stroke for the PCE and 5-year incident AF for CHARGE-AF). Perfect calibration is indicated by the hashed diagonal line, denoting perfect correspondence between predicted and observed risk. The Greenwood-Nam-D'Agostino test of calibration is shown for each plot (where a lower chi-squared value indicates better fit), the calibration slope (where a calibration slope of one is optimal), and integrated calibration index (ICI, a measure of prediction error in which lower values indicate more accurate predictions) is shown for each model. Since the PCE score comprises four separate models stratified on the basis of sex and race, the curve for each score is represented separately (see legend). Each plot also depicts a fitted calibration curve obtained using adaptive hazard regression[3] relating predicted risk and observed event risk.

Supplementary Figure 9. Longitudinal structure of C3PO

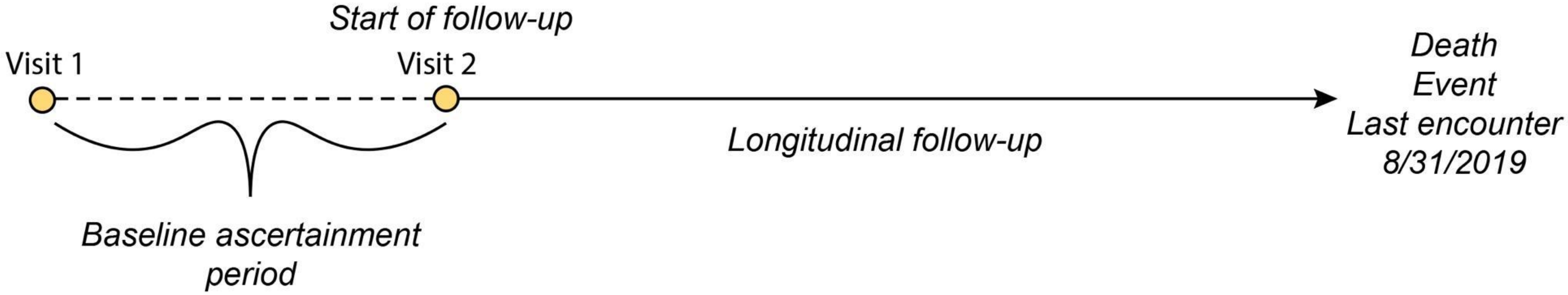

Depicted is an overview of the longitudinal analysis design of C3PO. All individuals meeting inclusion in C3PO had at least one pair of primary care office visits occurring between 1-3 years apart. The start of follow-up for longitudinal analyses for each individual was defined as the second visit of the earliest qualifying pair (i.e., Visit 2 in the diagram). The period between Visit 1 and Visit 2 was included to increase the likelihood that baseline characteristics (e.g., physical measurements, diagnoses) would be appropriately documented in the EHR prior to longitudinal modeling. Longitudinal follow-up then continued until the earliest of death, last encounter in the EHR, or August 31, 2019 (the administrative censoring date of C3PO).

Supplementary Figure 10. Overlap between C3PO and MGH primary care registry

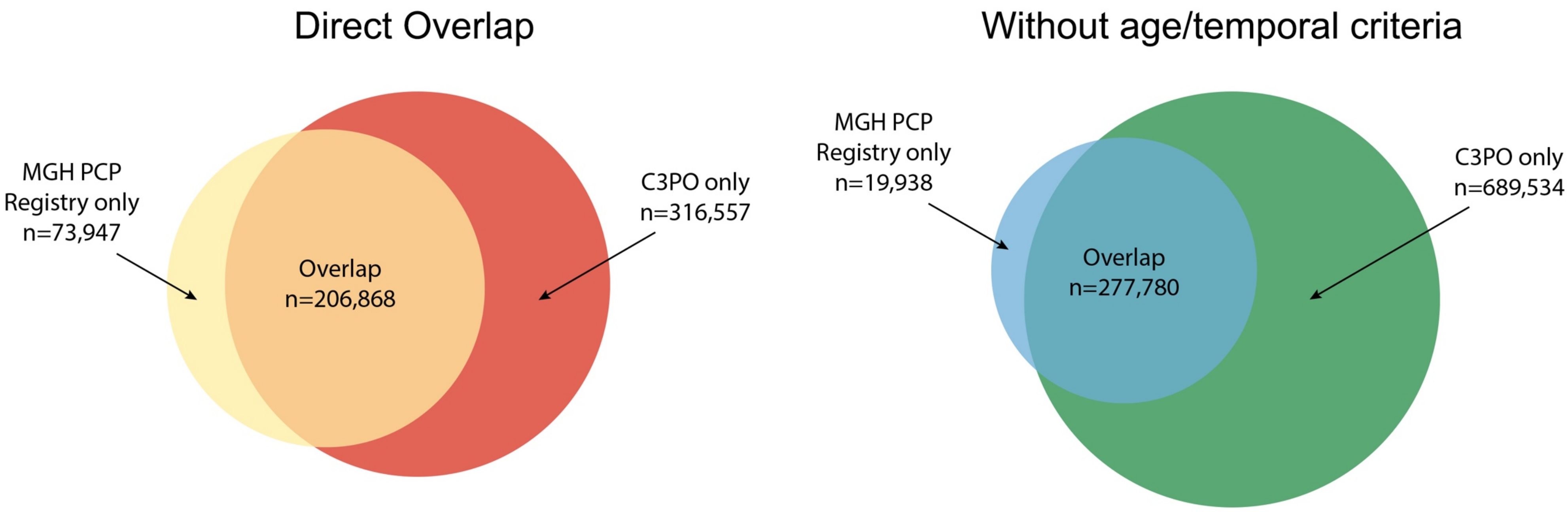

Depicted are Venn diagrams demonstrating overlap between the candidate C3PO cohort and an existing MGH-based primary care registry, to which we applied analogous selection methods. Of 280,815 individuals in the MGH registry meeting the specified temporal selection criteria, the substantial majority (n=206,868; 73.7%) were represented in the candidate C3PO cohort (left panel). The remaining discrepancy was attributed to differences in application of temporal selection criteria (1-3 year windows based on exact dates in C3PO, versus only calendar year data available in MGH registry), as well as exclusion of individuals under age <18 at the start of follow-up in C3PO. Without application of temporal or age selection criteria, 277,780 out of 297,718 (93.3%) of the MGH registry was represented in C3PO (right panel).

Supplementary Figure 11. MI/stroke and AF Convenience Sample construction

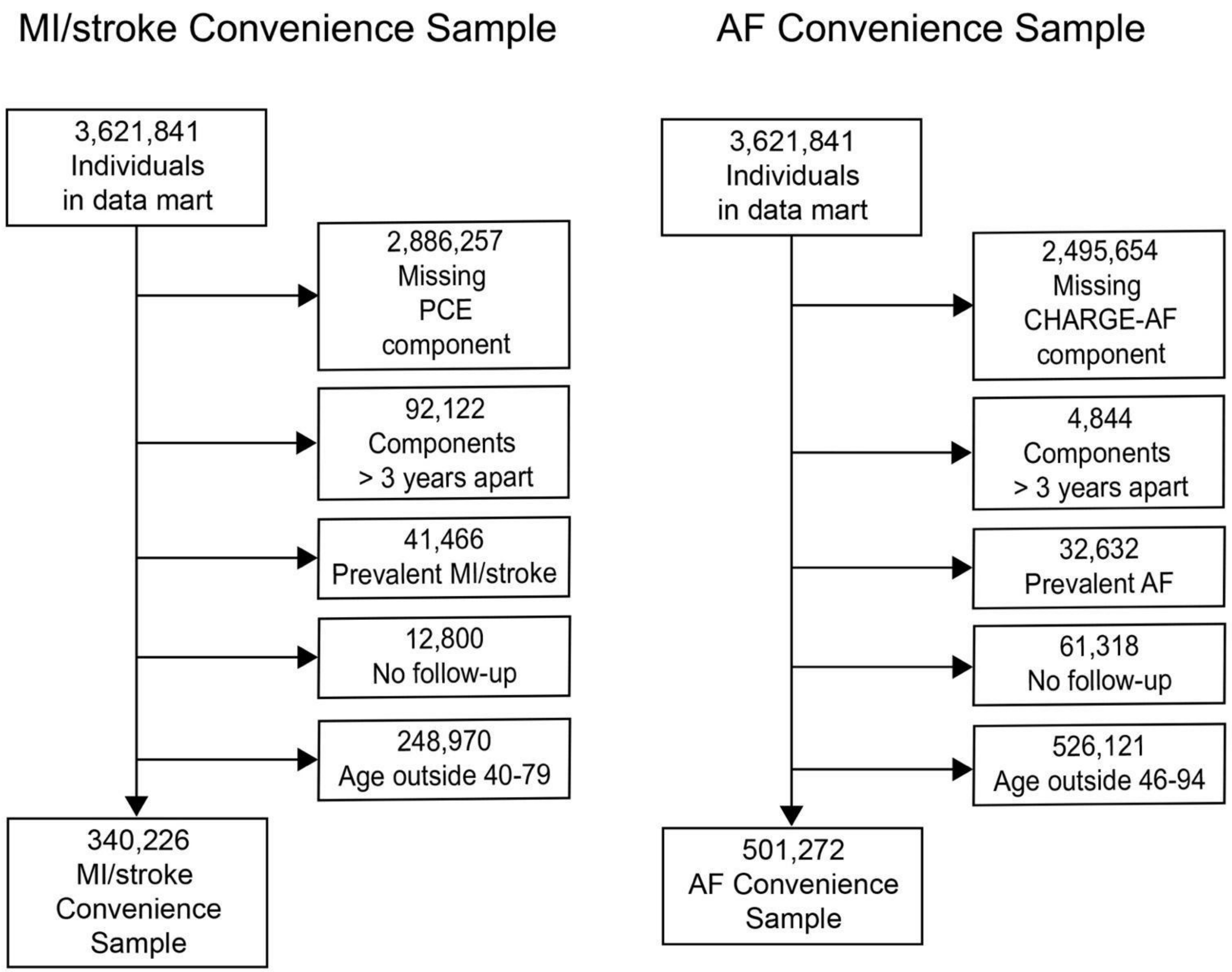

Depicted is an overview of the construction of the AF and MI/stroke Convenience Samples (see Methods in main text).

Supplementary Figure 12. Training and evaluation curves for fine-tuning Bio+Discharge Summary BERT

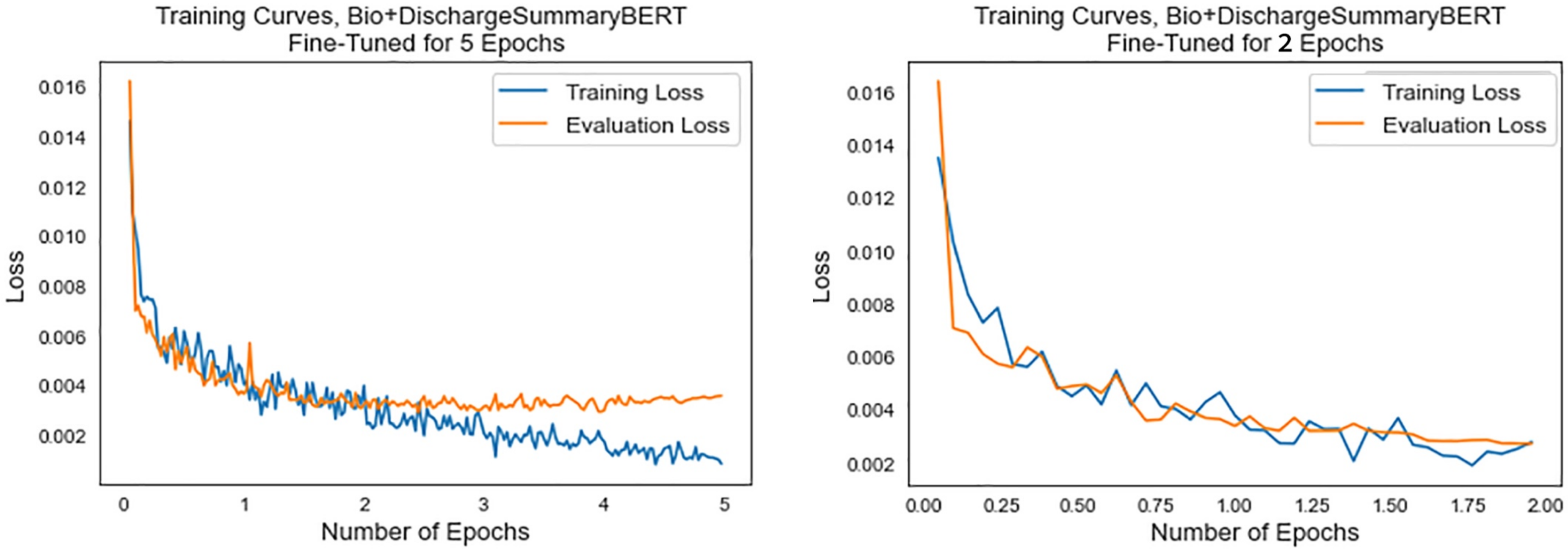

Depicted are training curves for the Bio+DischargeSummaryBERT natural language processing model graphing the training (blue) and evaluation (orange) loss (y-axis) across increasing training epoch (x-axis). The left plot shows Bio+DischargeSummaryBERT trained for 5 epochs (primary model) and the right plot shows Bio+DischargeSummaryBERT trained for 2 epochs. The model trained for 2 epochs exhibited evidence of underfitting resulting in substantially fewer blood pressure extractions (see Methods in main text).

Supplementary Figure 13. Training and evaluation curves for Bio+Discharge Summary BERT models across different sizes of training data.

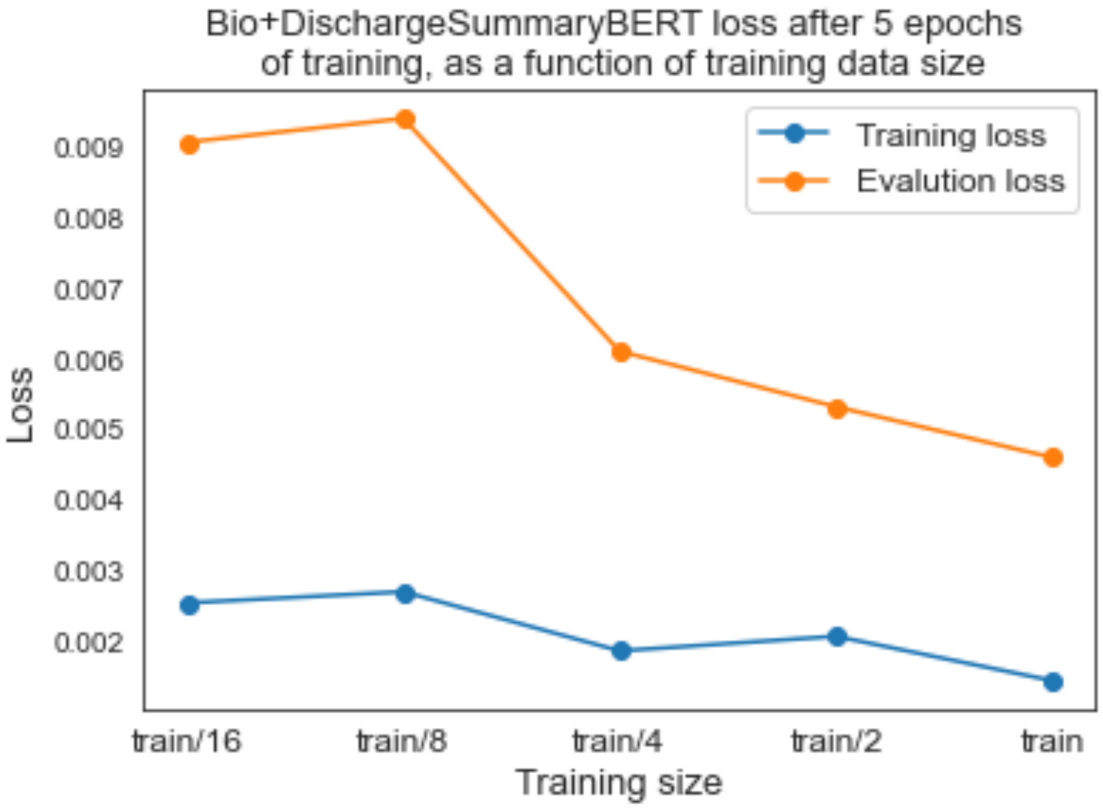

Depicted are training curves for the Bio+DischargeSummaryBERT natural language processing model with varying training set size. Each point depicts the training (blue) and evaluation (orange) loss (y-axis) after 5 epochs across increasing training set size (x-axis). The full training set contains 34,310 notes and 116,644 instances of vital signs, and each subset of training data halves the number of notes contained.

Supplementary References

1. Uno, H., Tian, L., Cai, T., Kohane, I. S. & Wei, L. J. A unified inference procedure for a class of measures to assess improvement in risk prediction systems with survival data. *Stat Med* 32, 2430–2442 (2013).
2. Demler, O. V., Paynter, N. P. & Cook, N. R. Tests of calibration and goodness-of-fit in the survival setting. *Stat Med* 34, 1659–1680 (2015).
3. Austin, P. C., Harrell, F. E. & Klaveren, D. Graphical calibration curves and the integrated calibration index (ICI) for survival models. *Statistics in Medicine* 39, 2714–2742 (2020).
4. Stevens, R. J. & Poppe, K. K. Validation of clinical prediction models: what does the 'calibration slope' really measure? *J Clin Epidemiol* 118, 93–99 (2020).
5. Steyerberg, E. W. & Vergouwe, Y. Towards better clinical prediction models: seven steps for development and an ABCD for validation. *Eur. Heart J.* 35, 1925–1931 (2014).
6. Khurshid, S., Keaney, J., Ellinor, P. T. & Lubitz, S. A. A Simple and Portable Algorithm for Identifying Atrial Fibrillation in the Electronic Medical Record. *Am. J. Cardiol.* 117, 221–225 (2016).
7. Wang, E. Y. *et al.* Initial Precipitants and Recurrence of Atrial Fibrillation. *Circ Arrhythm Electrophysiol* 13, e007716 (2020).
8. Hulme, O. L. *et al.* Development and Validation of a Prediction Model for Atrial Fibrillation Using Electronic Health Records. *JACC Clin Electrophysiol* 5, 1331–1341 (2019).
9. Arnett, D. K. *et al.* 2019 ACC/AHA Guideline on the Primary Prevention of Cardiovascular Disease: A Report of the American College of Cardiology/American Heart Association Task Force on Clinical Practice Guidelines. *Circulation* 140, e596–e646 (2019).
10. Alonso, A. *et al.* Simple risk model predicts incidence of atrial fibrillation in a racially and geographically diverse population: the CHARGE-AF consortium. *J Am Heart Assoc* 2, e000102 (2013).
