# Supplementary material for: Cohort design and natural language processing to reduce bias in electronic health records research
Source: NPJ Digit Med. 2022 Apr 8;5:47. doi: 10.1038/s41746-022-00590-0 (PMC8993873; doi:10.1038/s41746-022-00590-0)
Supplement: Supplementary file 2 — Reporting Summary Checklist [file 41746_2022_590_MOESM2_ESM.pdf]

nature portfolio

Corresponding author(s): Steven A. Lubitz

Last updated by author(s): Dec 31, 2021

Reporting Summary

Nature Portfolio wishes to improve the reproducibility of the work that we publish. This form provides structure for consistency and transparency in reporting. For further information on Nature Portfolio policies, see our Editorial Policies and the Editorial Policy Checklist.

Statistics

For all statistical analyses, confirm that the following items are present in the figure legend, table legend, main text, or Methods section.

n/a	Confirmed	
☐	☒	The exact sample size (n) for each experimental group/condition, given as a discrete number and unit of measurement
☐	☒	A statement on whether measurements were taken from distinct samples or whether the same sample was measured repeatedly
☐	☒	The statistical test(s) used AND whether they are one- or two-sided<br>*Only common tests should be described solely by name; describe more complex techniques in the Methods section.*
☐	☒	A description of all covariates tested
☐	☒	A description of any assumptions or corrections, such as tests of normality and adjustment for multiple comparisons
☐	☒	A full description of the statistical parameters including central tendency (e.g. means) or other basic estimates (e.g. regression coefficient) AND variation (e.g. standard deviation) or associated estimates of uncertainty (e.g. confidence intervals)
☐	☒	For null hypothesis testing, the test statistic (e.g. F, t, r) with confidence intervals, effect sizes, degrees of freedom and P value noted<br>*Give P values as exact values whenever suitable.*
☒	☐	For Bayesian analysis, information on the choice of priors and Markov chain Monte Carlo settings
☒	☐	For hierarchical and complex designs, identification of the appropriate level for tests and full reporting of outcomes
☐	☒	Estimates of effect sizes (e.g. Cohen's d, Pearson's r), indicating how they were calculated

Our web collection on statistics for biologists contains articles on many of the points above.

Software and code

Policy information about availability of computer code

Data collection	The JEDI data processing pipeline underlying C3PO, as well as data processing scripts underlying the current analyses, are publicly available under a BSD-3 Clause license at https://github.com/broadinstitute/jedi-public.
Data analysis	Analyses were performed using Python v3.833 and R v4.0.34 Two-sided p-values < 0.05 were considered statistically significant.

For manuscripts utilizing custom algorithms or software that are central to the research but not yet described in published literature, software must be made available to editors and reviewers. We strongly encourage code deposition in a community repository (e.g. GitHub). See the Nature Portfolio guidelines for submitting code & software for further information.

Data

Policy information about availability of data

All manuscripts must include a data availability statement. This statement should provide the following information, where applicable:

- Accession codes, unique identifiers, or web links for publicly available datasets
- A description of any restrictions on data availability
- For clinical datasets or third party data, please ensure that the statement adheres to our policy

MGB source data contain potentially identifying information and cannot be shared publicly. The JEDI data processing pipeline underlying C3PO, as well as data processing scripts underlying the current analyses, are publicly available under a BSD-3 Clause license at https://github.com/broadinstitute/jedi-public.

Field-specific reporting

Please select the one below that is the best fit for your research. If you are not sure, read the appropriate sections before making your selection.

☒ Life sciences ☐ Behavioural & social sciences ☐ Ecological, evolutionary & environmental sciences

For a reference copy of the document with all sections, see nature.com/documents/nr-reporting-summary-flat.pdf

Life sciences study design

All studies must disclose on these points even when the disclosure is negative.

Sample size	Study participants were initially identified using an MGB-based data mart containing tabular EHR data for >3.6 million individuals with at least one ambulatory visit between 2000-2018. Given our intent to identify individuals receiving primary care within the MGB system, we developed, validated, and applied rule-based heuristics to identify primary care office visits using Current Procedural Terminology (CPT) codes (Supplemental Table 1) and a manually curated list of 431 primary care clinic locations. To increase the probability that individuals received longitudinal primary care within MGB, we restricted the cohort to individuals with at least one pair of primary care visits occurring between 1-3 years apart. To allow for ascertainment of baseline clinical factors, we defined the start of follow-up for each individual as the second primary care visit of that individual's earliest qualifying pair (Supplemental Figure 1). In total, C3PO comprised 520,868 individuals (mean age 48 years, 61% women) with a median follow-up time of 7.2 years (quartile-1: 2.6, quartile-3: 12.9).
Data exclusions	C3PO comprises the electronic health record (EHR) data of 520,868 individuals aged 18-90 at the start of sample follow-up, selected from an ambulatory EHR database on the basis of receiving periodic primary care (i.e., ≥2 visits within 1-3 consecutive years, see text). Outlined in Figure 1
Replication	We acknowledge that the performance of our NLP model in other datasets remains unknown, although we anticipate that our overall approach of utilizing pre-trained language models with fine-tuning in the same or similar samples as those in which implementation is intended is likely to result in good performance across datasets.
Randomization	N/A
Blinding	N/A

Reporting for specific materials, systems and methods

We require information from authors about some types of materials, experimental systems and methods used in many studies. Here, indicate whether each material, system or method listed is relevant to your study. If you are not sure if a list item applies to your research, read the appropriate section before selecting a response.

Materials & experimental systems

n/a	Involved in the study	
☒	☐	Antibodies
☒	☐	Eukaryotic cell lines
☒	☐	Palaeontology and archaeology
☒	☐	Animals and other organisms
☐	☒	Human research participants
☒	☐	Clinical data
☒	☐	Dual use research of concern

Methods

n/a	Involved in the study	
☒	☐	ChIP-seq
☒	☐	Flow cytometry
☒	☐	MRI-based neuroimaging

Human research participants

Policy information about studies involving human research participants

Population characteristics	Detailed characteristics of individuals included in C3PO and each Convenience Sample are shown in Table 1.
Recruitment	Study participants were initially identified using an MGB-based data mart containing tabular EHR data for >3.6 million individuals with at least one ambulatory visit between 2000-2018. Given our intent to identify individuals receiving primary care within the MGB system, we developed, validated, and applied rule-based heuristics to identify primary care office visits using Current Procedural Terminology (CPT) codes (Supplemental Table 1) and a manually curated list of 431 primary care clinic locations. To increase the probability that individuals received longitudinal primary care within MGB, we restricted the cohort to individuals with at least one pair of primary care visits occurring between 1-3 years apart. To allow for ascertainment of baseline clinical factors, we defined the start of follow-up for each individual as the second primary care visit of that individual's earliest qualifying pair (Supplemental Figure 1). In total, C3PO comprised 520,868 individuals (mean age 48 years, 61% women) with a median follow-up time of 7.2 years (quartile-1: 2.6, quartile-3: 12.9).

Ethics oversight	Study protocols complied with the tenets of the Declaration of Helsinki and were approved by the Mass General Brigham Institutional Review Board.

Note that full information on the approval of the study protocol must also be provided in the manuscript.
